# Single-cell transcriptome landscape of circulating CD4^+^ T cell populations in autoimmune diseases

**DOI:** 10.1016/j.xgen.2023.100473

**Published:** 2024-01-03

**Authors:** Yoshiaki Yasumizu, Daiki Takeuchi, Reo Morimoto, Yusuke Takeshima, Tatsusada Okuno, Makoto Kinoshita, Takayoshi Morita, Yasuhiro Kato, Min Wang, Daisuke Motooka, Daisuke Okuzaki, Yamami Nakamura, Norihisa Mikami, Masaya Arai, Xuan Zhang, Atsushi Kumanogoh, Hideki Mochizuki, Naganari Ohkura, Shimon Sakaguchi

**Affiliations:** 1 Department of Experimental Immunology, Immunology Frontier Research Center, Osaka University, Osaka, Japan; 2 Department of Neurology, Graduate School of Medicine, Osaka University, Osaka, Japan; 3 Integrated Frontier Research for Medical Science Division, Institute for Open and Transdisciplinary Research Initiatives (OTRI), Osaka University, Osaka, Japan; 4 Faculty of Medicine, Osaka University, Osaka, Japan; 5 Department of Respiratory Medicine and Clinical Immunology, Osaka University Graduate School of Medicine, Osaka, Japan; 6 Department of Immunopathology, Immunology Frontier Research Center, Osaka University, Osaka, Japan; 7 Clinical Immunology Center, State Key Laboratory of Complex Severe and Rare Diseases, Peking Union Medical College Hospital, Chinese Academy of Medical Sciences and Peking Union Medical College, Beijing, China; 8 Department of Rheumatology, Beijing Hospital, National Center of Gerontology, Institute of Geriatric Medicine, Chinese Academy of Medical Sciences, Beijing, China; 9 Genome Information Research Center, Research Institute for Microbial Diseases, Osaka University, Osaka, Japan; 10 Center for Infectious Diseases for Education and Research, Osaka University, Osaka, Japan; 11 Department of Frontier Research in Tumor Immunology, Graduate School of Medicine, Osaka University, Osaka, Japan; 12 Department of Experimental Immunology, Institute for Life and Medical Sciences, Kyoto University, Kyoto, Japan

**Keywords:** CD4^+^ T cells, autoimmune diseases, single-cell RNA-seq, GWAS, immunogenomics

## Abstract

CD4^+^ T cells are key mediators of various autoimmune diseases; however, their role in disease progression remains unclear due to cellular heterogeneity. Here, we evaluated CD4^+^ T cell subpopulations using decomposition-based transcriptome characterization and canonical clustering strategies. This approach identified 12 independent gene programs governing whole CD4^+^ T cell heterogeneity, which can explain the ambiguity of canonical clustering. In addition, we performed a meta-analysis using public single-cell datasets of over 1.8 million peripheral CD4^+^ T cells from 953 individuals by projecting cells onto the reference and cataloging cell frequency and qualitative alterations of the populations in 20 diseases. The analyses revealed that the 12 transcriptional programs were useful in characterizing each autoimmune disease and predicting its clinical status. Moreover, genetic variants associated with autoimmune diseases showed disease-specific enrichment within the 12 gene programs. The results collectively provide a landscape of single-cell transcriptomes of CD4^+^ T cell subpopulations involved in autoimmune disease.

## Highlights

• Identified 18 cell types and 12 gene programs from blood CD4^+^ T cells
• Meta-analysis enrolling 1.8M cells outlines CD4^+^ T alterations in autoimmune diseases
• Observed disease-specific heritability enrichment within CD4^+^ T cell gene programs

## Graphical abstract

**Figure:**
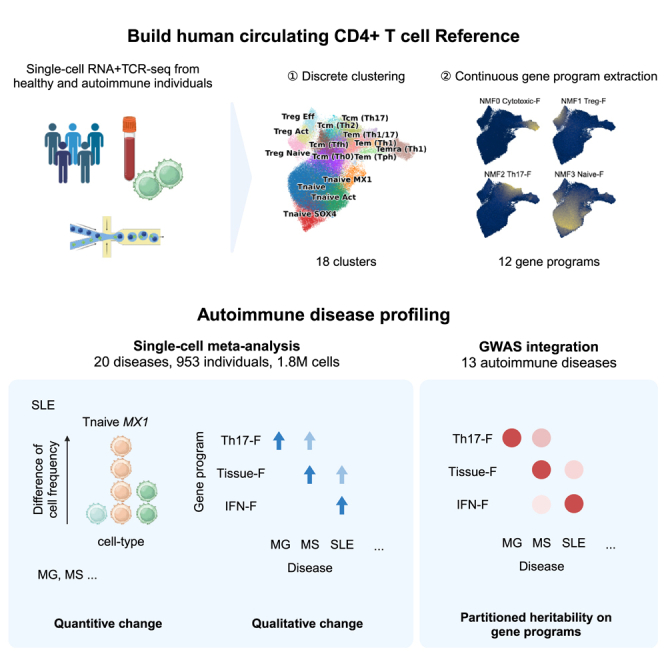

## Teaser

Yasumizu, Takeuchi, Morimoto, et al. compile a comprehensive reference of circulating CD4^+^ T cells, encompassing 12 gene programs and 18 clusters. They reveal disease-specific cellular characteristics through the integration of genome-wide association studies and single-cell meta-analysis. This work provides insights for precision medicine.

## Introduction

Autoimmune disorders encompass various conditions where the immune cells display abnormal reactivity toward normal tissues. These diseases are multifactorial, polygenic, and prevalent, affecting 3%–5% of the population.^1^^,^^2^ Numerous studies have highlighted the role of CD4^+^ T cells in the onset or exacerbation of these diseases.^2^^,^^3^

CD4^+^ T cells exhibit a variety of states (e.g., naive, memory), polarizations (e.g., Th1, Th2, Th17, T follicular helper or Tfh), and also include a distinct subpopulation engaged in the maintenance of self-tolerance and homeostasis (regulatory T cells or Tregs).^4^^,^^5^ While a great deal of effort has been devoted to the detailed classification of CD4^+^ T cells, the complete picture of heterogeneity and its relationship to diseases is still controversial. Furthermore, making consistent assessments across reports is challenging since these reports were based on inconsistent cellular classifications.

The recent emergence of single-cell analysis has greatly contributed to the elucidation of cellular diversities through unbiased profiling.^6^^,^^7^^,^^8^^,^^9^^,^^10^^,^^11^ In addition, single-cell RNA sequencing (scRNA-seq) is suitable for robust cross-dataset data integration, allowing large-scale investigations.^12^^,^^13^^,^^14^^,^^15^ Furthermore, in the field of T cell research, a number of tools have enabled precise T cell profiling across various contexts such as chronic infection and tumor-infiltrating T cells.^16^^,^^17^ On the other hand, conventional clustering and marker gene detection strategies for single-cell analysis possess the following weaknesses: (1) cell fraction definition requires arbitrary boundaries, (2) marker genes for clusters can be occupied by redundant genes or uninterpretable genes, such as long noncoding or ribosomal genes, due to the influence of larger cell population structures, and (3) pairwise differentially expressed gene detection cannot capture global gene variation across multiple clusters. Several studies have attempted to tackle these issues.^18^^,^^19^ Especially, in some previous reports, non-negative matrix factorization (NMF)^20^ has been utilized to extract both cell-type identity and cellular activity programs, thereby addressing the profiling of complex cell populations.^21^^,^^22^ However, it still harbored complexity in determining the parameters and in the flexible integration between different datasets.

Here, we construct a consensus reference for CD4^+^ T cells in peripheral blood from autoimmune and healthy individuals covering various inflammatory conditions. The reference consists of 18 cell types defined by a conventional clustering strategy and 12 transcriptomic gene programs extracted by conducting decomposition using NMF without boundaries, which overcame the weakness of existing clustering-based single-cell analyses. The results show that diverse CD4^+^ T cell features were formed by a combination of 12 independent gene programs. We also illustrate that the gene features obtained by NMF could be projected to other bulk/scRNA-seq data to help interpret various datasets. Using these frameworks to examine the genetic contribution and subsequent changes of CD4^+^ T cells in autoimmunity, we performed a meta-analysis that enrolled over 1.8 million CD4^+^ T cells using published single-cell data of 20 diseases and integrated genome-wide association study (GWAS) statistics for 180 traits with our dataset. These analyses provided a full picture of CD4^+^ T cells in autoimmune diseases from the perspective of phenotypes and genetics.

## Results

### Single-cell profiling of peripheral CD4^+^ T cells from healthy and autoimmune donors

**Figure 1:**
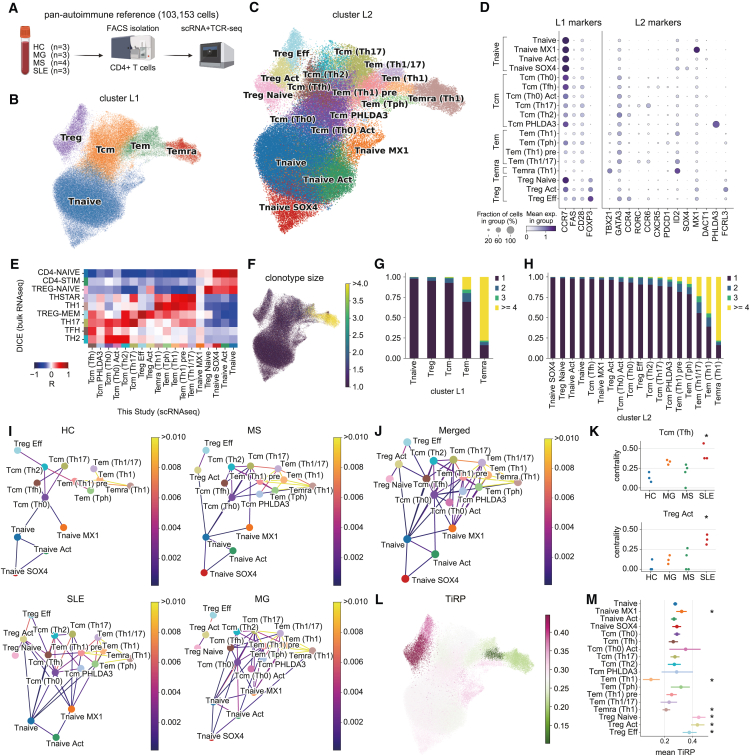
Global profiling of CD4^+^ T cells (A) Sample collection strategy. HC, healthy control; MG, myasthenia gravis; MS, multiple sclerosis; SLE, systemic lupus erythematosus. (B and C) Cluster layer 1 (L1) and layer 2 (L2) on Uniform Manifold Approximation and Projection (UMAP) embeddings. (D) Dot plot depicting signature genes' mean expression levels and percentage of cells expressing them across clusters. Marker genes for the plot were manually selected. See also Figure S1C for automatically extracted marker genes. (E) Expression correlation of clusters with bulk RNA-seq for sorted CD4^+^ T cell fractions generated by the DICE project. (F) UMAP plot showing clonotype size. The color indicates the number of cells sharing the same T cell receptor (TCR) sequences. (G and H) Clonotype size distributions across clusters. (I and J) TCR similarity networks in autoimmune patients, healthy donors (I), and all donors (J). TCR similarity was calculated for each sample, and only edges where overlapping clonotypes were detected in ≥2 (I) or ≥4 (J) samples are depicted as robust overlaps. The edge color indicates the average TCR similarity of all samples. (K) Degree centrality of TCR networks. Significance across clusters was calculated by one-way analysis of variance and, after multiple test corrections by false discovery rate, Tcm (Tfh) and Treg Act were retained as significant cell types. Then, pairwise Tukey-HSD post hoc tests were performed. ∗p_adj_ < 0.05 in comparison with HCs. (L) TCR-intrinsic regulatory potential (TiRP) score distributions on UMAP plot. Mean scores for each cluster L2 were shown. (M) TiRP score distribution across cluster L2. The dot shows the mean, and the confidence interval (CI) shows 95% CI of the bootstrap distribution of means (n = 1,000). Adjusted p values of significant clusters, Tnaive *MX1* 1.29 × 10^−2^, Tem (Th1) 5.82 × 10^−11^, Temra (Th1) 4.84 × 10^−22^, Treg Naive 2.14 × 10^−13^, Treg Act 2.14 × 10^−13^, Treg Eff 3.82 × 10^−5^ (two-sided Mann-Whitney U test was performed for one cluster vs. the other clusters iteratively).

To characterize CD4^+^ T cells in various autoimmune properties, we performed scRNA-seq and T cell receptor (TCR)-seq using droplet-based single-cell isolation technology and profiled CD4^+^ T cells, which were collected from three healthy donors, three myasthenia gravis (MG) patients, four multiple sclerosis (MS) patients, and three systemic lupus erythematosus (SLE) patients (Figures 1A–1D; Table S1). After quality control (QC), 103,153 cells were retained and used for the downstream analyses. As the primary layer of clustering (cluster L1), we identified a dynamic differentiation from a naive state via an effector state to a terminally differentiated state. In cluster L1, CD4^+^ naive T cells (Tnaive) (*CCR7*^+^ *FAS*^−^), CD4^+^ central memory T cells (Tcm) (*CCR7*^+^ *FAS*^+^), CD4^+^ effector memory T cells (Tem) (*CCR7*^−^ *FAS*^+^), and CD4^+^ terminally differentiated effector memory T cells (Temra) (*FAS*^+^ *CD28*^−^) were observed with distinct gene expression patterns (Figures 1B, 1D, and S1A; Table S2). Tregs were also observed as a distinct cluster with the expression of the master regulator *FOXP3*. Next, we further divided the cells into 18 clusters as the secondary layer, cluster L2 (Figures 1C and S1B–S1D; Table S3). For example, we broke down cluster L1 cells into several T cell subclusters according to well-known transcription factors and chemokine receptors such as Tcm cells into Tfh (Tfh; *CXCR5*, *PDCD1*), Th2 (*GATA3*, *CCR4*), Th17 (*RORC*, *CCR6*); Tem cells into Th1/17 (*TBX21*/Tbet, *RORC*), Th1 (*TBX21*/Tbet); Temra cells into Th1 (Figures 1C, 1D, and S1A–S1D). Treg cells were divided into three clusters; Treg Naive (*CCR7*), Treg Activated (*ID2*), and Treg Effector (*CCR4*) (Figures 1C, 1D, and S1A–S1D). In addition, several minor clusters were found, such as Tnaive *MX1*, which preferentially expresses interferon signature genes (Figures 1C, 1D, S1A, S1C, and S1D). Transcriptome profiles of each cluster were concordant with bulk RNA-seq data from fluorescence-activated cell sorting-sorted CD4^+^ T cell fractions provided by the Database of Immune Cell Expression, Expression quantitative trait loci and Epigenomics (DICE) project^23^ (Figure 1E). Furthermore, based on the consistent expression of marker genes across all samples, we confirmed that the batch correction by Harmony was performed appropriately (Figures S1E and S1F). We found a *CXCR5*^−^ *PDCD1*^+^ cluster occupying 1% in CD4^+^ T cells whose marker genes corresponded to the canonical marker for T peripheral helper (Tph) cells^24^^,^^25^^,^^26^ in Tem (Figures S1A and S1G). The population was annotated as circulating Tph, although a few cells with the expression *CXCR5*^−^ *PDCD1*^+^ were also observed in broader populations such as Tcm and Temra (Figures S1A, S1C, and S1H). Overall, we identified cell populations of peripheral CD4^+^ T cells from healthy and autoimmune states using scRNA-seq.

### TCR features across CD4^+^ T cells reflect cellular properties

Because TCR responses shape T cell functions and differentiation, TCR diversities and overlaps provide useful information for the properties and relationships of populations. Therefore, we analyzed single-cell TCR features sequenced along with gene expression. Clonotype sizes and diversity across cluster L1 populations revealed that Temra was most clonally expanded, followed by Tem, Tcm, Treg, and Tnaive (Figures 1F and 1G). Similarly, in cluster L2 populations, Temra (Th1), Tem (Th1), and Tem (Th1/17) possessed a limited number of clonotypes, whereas Tnaive and Treg Naive maintained diverse clonotype pools (Figure 1H). The TCR similarity network showed a repertoire sharing between neighboring clusters, Tnaive and Tcm, Tcm and Tem, and Tem and Temra, while the distal connection such as from Tnaive to Temra was not observed, suggesting stepwise development from Tnaive to Tcm, Tem, and Temra (Figures 1I, 1J, and S2B). The repertoires were also mutually shared within Tcm cell populations, suggesting the plasticity of T cell polarization against the same epitopes. In addition, no substantial overlap was observed between the repertoires of Treg Naive and conventional T cells (Tconvs), whereas Treg Act and Treg Eff demonstrated shared repertoires with Tcm populations (Figures 1I, 1J, and S2B). We also measured the centrality of TCR networks for each cell type to evaluate the differentiation potential of each cluster. The centrality of Tcm (Th0), Tem (Th1) pre, and Tnaive were consistently high, suggesting that these cells possess the possibility to differentiate into a variety of cell types (Figure S2C). In addition, TCR networks differed depending on the disease states (Figure 1I). Especially the centrality of Tcm (Tfh) and Treg Act were higher in SLE (Figures 1K and S2D). These results indicated that the kinetics of CD4^+^ T cell differentiation varied depending on the disease state.

Previous studies have shown that T cells with stronger TCR stimulation within the thymus are more likely to differentiate into Tregs than Tconvs.^27^ Therefore, Tregs have specific TCR properties, such as hydrophobicity in complementary determining region 3 regions.^28^ We measured the Tregness of TCRβ chains (TCR-intrinsic regulatory potential [TiRP] score^28^) and found that the mean TiRP score was higher in Treg cells compared with Tconv cells (Figures 1L and 1M). On the contrary, Tem (Th1) showed a low TiRP score, indicating that Tem (Th1) has experienced stimulation with non-self antigens. Among Treg cells, Treg Naive and Treg Act showed higher TiRP scores than Treg Eff. It has been thought that naive Tregs contain predominantly thymic differentiated Tregs (tTregs), while effector Tregs are compensated by peripherally differentiated Tregs in addition to tTregs.^29^ This notion was concordant with our observations that naive Tregs had the strongest Treg characteristics in the TCRs and that Treg Act and Eff shared TCRs with Tconvs (Figures 1I, 1J, and S2E). Furthermore, in MS patients, the TiRP scores of the Treg Act were significantly low, reflecting disease-dependent Treg compensation by Tconvs (Figure S2F). Overall, TCR repertoires provided valuable insights into T cell characteristics and relationships during the differentiation.

### Decomposition of cellular programs using NMF

**Figure 2:**
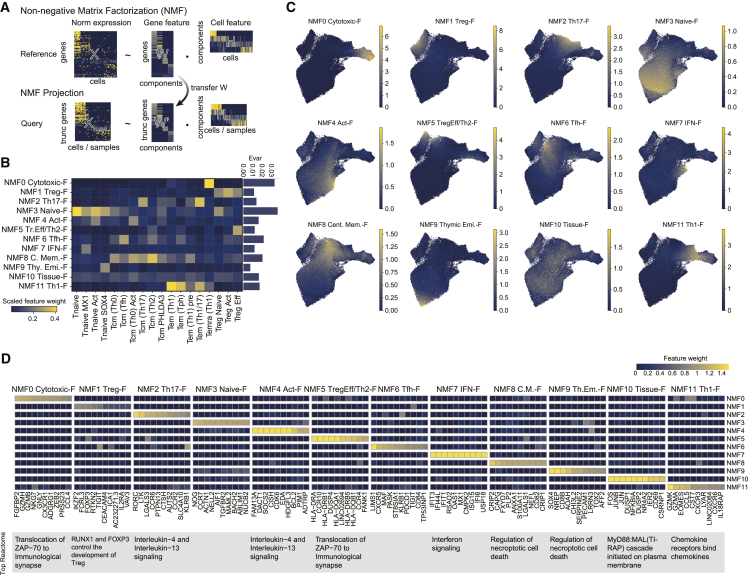
NMF captured 12 CD4^+^ T cell features (A) Schematic view of NMF and NMF projection (NMFproj). (B) Matrixplot showing the mean scaled NMF feature weight for each cluster L2 population. The explained variance (Evar) is also shown on the right. The NMF feature weight is scaled by the maximum value for each feature for visualization. (C) NMF cell feature value on UMAP plots. (D) Gene features for each component. The top 10 genes for each feature were selected. The 12 gene features are annotated using top genes and previous reports as NMF0 Cytotoxic-Feature (F), NMF1 Treg-F, NMF2 Th17-F, NMF3 Naive-F, NMF4 Activation-F (Act-F), NMF5 TregEff/Th2-F, NMF6 Tfh-F, NMF7 IFN-F, NMF8 Central Memory-F, NMF9 Thymic emigrant-F, NMF10 Tissue-F, and NMF11 Th1-F. The Reactome pathway with the smallest p value for each gene feature is shown below the heatmap (Figure S3B; Table S5).

Next, we attempted to identify cellular programs within and across cell types. We noticed that conventional clustering and marker gene detections could fail to capture meaningful clusters and genes. For example, differentially expressed genes in our reference included overlapping genes among Th1 cell populations and nonsense genes in Tnaive cells, suggesting that the conventional marker gene detection is insufficient for CD4^+^ T cells (Figure S1C). We suspected that artificially delineating in the clustering process is unsuitable for a gradual population such as CD4^+^ T cells. In addition, because marker gene detections are performed by pairwise comparison, global representations across cell types cannot be detected. To overcome these limitations, we applied non-negative matrix factorization^20^ to normalized gene expression of our scRNA-seq data. NMF and its derivative method, consensus NMF (cNMF),^22^ have been widely used in single-cell analysis as a technique to extract both cell-type identity and cellular activity as gene programs. NMF unbiasedly dissected gene expression profiles into a gene feature matrix ***W*** and a cell feature matrix ***H*** (Figure 2A). To determine the number of components, we assessed the explained variances and maximum inter-component correlations and selected 12 for the number of components as they kept sufficient information and were not redundant (Figure S3A; STAR Methods). Based on the gene feature profiles and the enriched pathways, we annotated the NMF components (Figures 2B–2D and S3B; Tables S4 and S5). Several factors were related to T cell polarization, such as Treg-Feature (Treg-F) (NMF1; genes with high weights; *IKZF2*, *FOXP3*), Th17-F (NMF 2; *RORC*, *CCR6*), TregEff/Th2-F (NMF5; HLA class II genes, *CCR10*, *CCR4*), Tfh-F (NMF6; *TIGIT*, *CXCR5*), Th1-F (NMF11; *GZMK*, *EOMES*, *CXCR3*), and differentiations such as Naive-F (NMF3; *CCR7*, *TCF7*), Central Memory-F (NMF8; *S100A8*, *ANXA1*), and Cytotoxic-F (NMF0; *GZMB*, *NKG7*). NMF5 was enriched in both Th2 and Treg Eff, suggesting that effector Treg cells and Th2 cells may be controlled by the shared program as previously suggested^30^ (Figure 2B). NMF6 (Tfh-F) also demonstrated moderate activity in Treg Act, suggesting an overlap between Treg Act and T-follicular regulatory (Tfr) cells^31^ (Figure 2B). NMF11^high^ cells were enriched in Tem (Tph), Tem (Th1), and Tem (Th1/17) cells, showing a wide range of Th1ness gene usage across these subtypes. Moreover, NMF7 was a type I interferon signature gene component enriched in Tnaive *MX1* (Figures 1C and S3B). Intriguingly, NMF10 captured a global feature across cell types consisting of AP-1 family genes (*JUNB*, *FOS*), *NFKBIA*, *CD69*, and *CXCR4* (Figure 2D). This feature was concordant with tissue-homing T cells observed in the thymoma of MG patients^7^ and the central nervous system of neurodegenerative disease patients,^32^ and was labeled as Tissue-F. NMF4 (Act-F) was related to *IL7R* signaling, which is an essential survival and differentiation signal.^33^ The proportion of explained variance (Evar) showed the most drastic variations in the peripheral CD4^+^ T cells were differentiation from Tnaive to Tcm, Tem, and Temra, and the polarizations were relatively smaller changes and independent of the differentiation programs (Figure 2B). Altogether, NMF succeeded in the decomposition of peripheral CD4^*+*^ T cell gene programs into 12 components and showed that complex CD4^*+*^ T cell populations were represented by a simple combination of the 12 components.

### NMF projection enables fast interpretation of various CD4^+^ T transcriptome datasets

One of the biggest challenges in single-cell analysis is the integration of datasets. To achieve a simple integration, we expanded the NMF framework to allow the projection of the pre-computed gene feature matrix onto other datasets by developing a bioinformatics tool, NMFproj (Figure 2A, https://github.com/yyoshiaki/NMFprojection). Recognizing the prevalent use of cNMF, we also designed NMFproj to be directly compatible with cNMF outputs. To measure how the NMF features explain the variance of the query dataset, we introduced a QC metric named the proportion of overlapped highly variable genes (POH) and Evar for all factors (Evar all) (Figure S3C). A low POH indicates that the query dataset has much variability other than the NMF features evaluated by NMFproj. We applied NMFproj to various datasets to validate the scalability (supplemental note). Analysis of bulk RNA-seq of sorted peripheral CD4^+^ T cells provided by the DICE project^23^ demonstrated that each fraction was well represented by the 12 NMF gene features (POH: 0.272; Evar all: 0.839, Figure S3D). Miyara classification^34^ categorizes CD4^+^ T cells into six distinct fractions (Fr. I to Fr. VI) based on the expression of CD45RA and CD25. This classification enables the distinction between Tregs and Tconvs, as well as naive and activated cell states. However, the identity of borderline populations, such as Fr. III (CD45RA^−^ CD25^int^), remains less understood. Using NMFproj, we re-evaluated this classification and found that Fr. III possesses Th17 type characteristics in line with the original report^34^ (POH: 0.542; Evar all: 0.823, Figure S3E; supplemental note). In addition, profiling of circulating Tph cells^24^ revealed that Tph cells possessed both NMF6 (Tfh-F) and NMF11 (Th1-F) in concordance with Tem (Tph) we defined as cluster L2 (POH: 0.134; Evar all: 0.816, Figure S3F; supplemental note). We also attempted to utilize NMFproj for the QC of *in vitro* induced Treg (iTreg) cells of mouse^35^ and found that iTreg^+^ cells induced in optimized conditions for enhancing Treg functionality showed higher NMF1 (Treg-F) values than conventional iTreg cells (POH: 0.182; Evar all: 0.844, Figure S3G; supplemental note). We also applied NMFproj to scRNA-seq datasets of cross-tissue immune cells^36^ (Figure S6, POH: 0.560; Evar all: 0.332 in CD4^+^ T cells, supplemental note), pan-cancer tumor-infiltrating CD4^+^ T cells^15^ (Figure S4A, POH: 0.530; Evar all: 0.338, supplemental note), and mouse splenocytes^37^ (Figure S4B, POH: 0.394; Evar all: 0.146, supplemental note). CD4^+^ T programs defined by circulating CD4^+^ T cells were adequately projected to various conditions, with permissive POH values and lower Evar all. Given the variance observed in Evar all between circulating and tissue-derived datasets, we sought to explain this by comparing the *ab initio* NMF in a pan-cancer dataset with the NMF defined in circulating CD4^+^ T cells. We found that the majority of circulating CD4^+^ T factors had correlating factors in the NMF defined by the CD4^+^ tumor-infiltrating lymphocyte (TIL) data (Figures S5A–S5D). For example, NMF7 (IFN-F) in circulating CD4^+^ T corresponded to NMF15 in *ab initio* NMF defined by the pan-cancer dataset (Figures S4A and S5D). However, NMF8 (Cent. Mem.-F) and NMF4 (Act-F), which were broadly elevated or almost inactive within the tumor, were not included in the NMF defined for TILs. In addition, some factors, including NMF17, which contains *CXCL13* and *IL21*, were only included in NMF components defined by TILs (Figure S5D). From these results, it seems that the NMF defined in circulating CD4^+^ T cells can be correctly projected by NMFproj even to TILs, but some tissue-specific factors may need to be carefully evaluated. Furthermore, cell-specific qualitative changes have been reported in autoimmune diseases, such as Treg dysfunction in SLE,^38^ and we hypothesized that NMFproj could be used to detect these changes in individual cell populations. To test this, we applied NMFproj to bulk RNA-seq data of sorted peripheral CD4^+^ T cell fractions from various autoimmune patients.^39^ NMFproj detected a subset-specific gene program robustly even in a variety of autoimmune disease conditions (Figure S7A; supplemental note). The results showed cell-type-wide enhancement of NMF7 (IFN-F) in SLE and mixed connective tissue disease and hampered NMF1 (Treg-F) in Fr.I nTregs (CD45RA^+^ CD25^+^) in SLE patients as previously reported^38^ (Figure S7B). These results indicated that NMFproj could robustly assess the qualities of CD4^+^ T cells in various tissues and disease states, regardless of bulk/single cell or human/mouse. We were also able to perform analyses while maintaining interpretability in a large dataset spanning multiple diseases and cell types.

### Meta-analysis of CD4^+^ T cells in various autoimmune diseases

**Figure 3:**
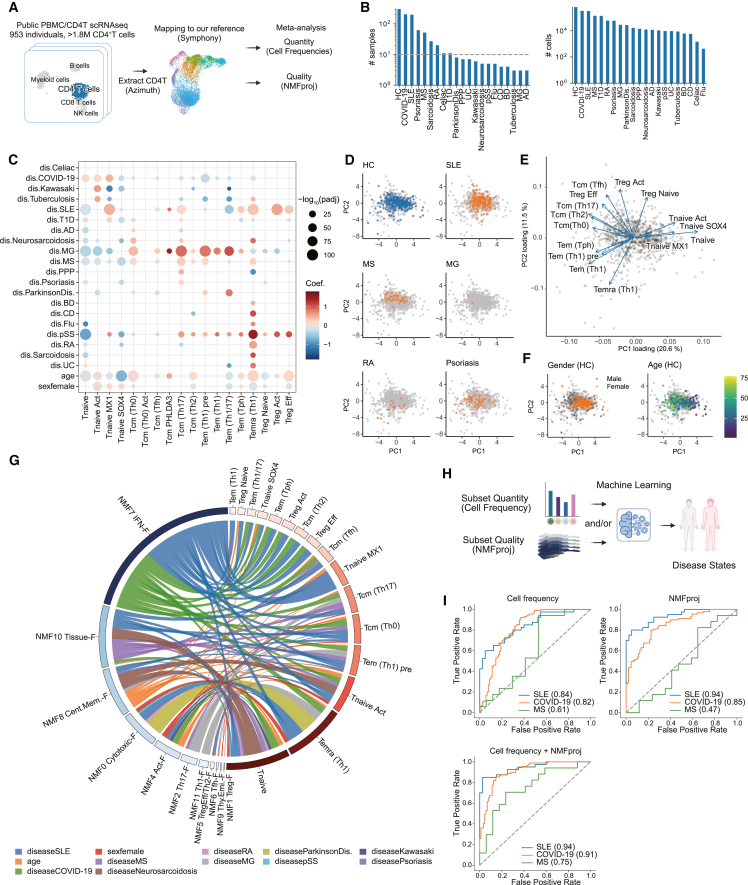
Pan-autoimmune meta-analysis of peripheral CD4^+^ T cells (A) Strategy for meta-analysis of peripheral CD4^+^ T cells across diseases. First, CD4^+^ T cells were extracted from peripheral blood mononuclear cell (PBMC) scRNA-seq datasets using Azimuth.^62^ Extracted CD4^+^ T cells were mapped on our reference using Symphony^14^ with a batch correction. Mapped cells were used to assess cell frequency and NMF cell features for each cluster. (B) Bar plots showing the number of samples (left) and the number of CD4^+^ T cells (right) enrolled in the meta-analysis. The dashed line in the left plot indicates a sample size of 10. Celiac, celiac disease; Kawasaki, Kawasaki disease; T1D, type 1 diabetes; AD, Alzheimer’s disease; PPP, palmoplantar pustulosis; ParkinsonDis., Parkinson’s disease; BD, Behçet’s disease; CD, Crohn’s disease; Flu, influenza; pSS, primary Sjögren syndrome; RA, rheumatoid arthritis; UC, ulcerative colitis. (C) Dot plot showing changes in cell frequency at cluster L2 resolution. Dot colors show coefficients, and sizes show the significance of the generalized linear model (GLM) (STAR Methods). Detailed statistics can be found in Table S8. Only significant dots (p_adj_ < 0.05) are shown. (D–F) Principal-component analysis (PCA) plots of samples based on cell frequencies. Sample distributions for each disease state (D), loading vectors for each cell type (E), and sample characteristics in healthy donors (F) are shown. (G) Chord diagram showing the top 100 significant associations with positive coefficients between NMF features and cells in each condition, calculated by GLM (STAR Methods). Detailed statistics are shown in Table S9. The thickness of edges indicates the coefficient of GLM, and colors indicate conditions such as diseases, gender, and age. (H) Strategy for predicting autoimmune states from CD4^+^ T cell profiles using machine learning framework. As the input parameters, one model took only cell frequency, age, and gender (without NMFproj), while the other took cell frequency, NMF cell features in Tcm (Th0) and Tnaive, age, and gender (with NMFproj). (I) Receiver operating characteristic curves of logistic regression models trained by cell frequencies (top left), by NMFproj values in Tnaive and Tcm (Th0) (top right), and both cell frequencies and NMFproj values (bottom). SLE, COVID-19, and MS patients were trained on 159, 116, and 35 patients with the same number of healthy subjects, and evaluated on 40, 89, and 17 patients and the same number of healthy subjects from independent datasets. Numbers in parentheses indicate the area under the curve (AUC).

To extend CD4^+^ T cell profiling to various autoimmune and infectious diseases, we performed a meta-analysis using publicly available single-cell data.^6^^,^^8^^,^^40^^,^^41^^,^^42^^,^^43^^,^^44^^,^^45^^,^^46^^,^^47^^,^^48^^,^^49^^,^^50^^,^^51^^,^^52^^,^^53^^,^^54^^,^^55^^,^^56^^,^^57^^,^^58^^,^^59^^,^^60^^,^^61^ We integrated publicly available datasets with two strategies: (1) quantitative evaluation of cell frequencies by mapping to our reference and (2) evaluation of qualitative changes per cell type using NMFproj. We extracted CD4^+^ T cells from peripheral blood mononuclear cells (PBMCs) using Azimuth^62^ and then mapped them to our reference using Symphony^14^ (Figure 3A, the pipeline is available at https://github.com/yyoshiaki/screfmapping). Reference mapping using Symphony achieved accurate label transfer at both cluster L1 and L2 resolutions (Figures S8A–S8D, accuracy = 0.923 and 0.781, adjusted Rand index = 0.825 and 0.631 in cluster L1 and L2, respectively), and the result was concordant with those by other tools, CellTypist^36^ and ProjecTILs^16^ (Figures S8E and S8F). The suitability of Symphony mapping for conditions such as infectious diseases was also verified using silhouette scores (Figure S8G). We collected 1,809,668 CD4^+^ T cells collected from 647 cases and 306 controls from 25 projects (Figures 3B and S9A; Table S6). For quality assurance, only datasets in which both HC and patients were present and at least three cases were included were used. As a prominent change, Tnaive decreased and Temra increased in various autoimmune diseases (Figure S9B; Table S7). It has been reported that Temra increased in the peripheral blood of rheumatoid arthritis (RA), MS, ulcerative colitis (UC), and Crohn’s disease (CD) patients,^63^^,^^64^^,^^65^^,^^66^ which was consistent with the present data. Kawasaki disease and type 1 diabetes (T1D) were exceptions among autoimmune diseases, with a slight increase in Tnaive and no significant change in Tcm, Tem, and Temra (Figure S9B), as reported previously.^67^^,^^68^ At cluster L2 resolution, we found that Tnaive *MX1* increased in COVID-19, SLE, T1D, and primary Sjögren syndrome (pSS) patients (Figure 3C). The type I IFN response is essential for viral elimination and has been reported to be associated with COVID-19 pathology^69^ and also known to be associated with SLE,^70^ pSS,^71^ and T1D.^72^ Our meta-analysis could detect these effects as the increase of Tnaive *MX1*. Moreover, in our meta-analysis, Tcm (Th17) was increased in various diseases, including previously reported diseases such as MG,^73^ MS,^74^^,^^75^ and psoriasis.^76^ Regarding Tregs, we reported that Fr. II (CD45RA^−^ CD25^+^) is increased in sarcoidosis, while Fr. I and III are increased in active SLE.^34^ Another group reported an increase of Tregs in pSS.^77^ In concordance with these observations, Treg increased in SLE, neurosarcoidosis, sarcoidosis, and pSS, especially for Treg Eff in neurosarcoidosis and for Treg Act and Treg Eff in SLE patients (Figures 3C and S9B). Interestingly, the acute infection response to COVID-19 showed an increase in Tnaive, whereas influenza infection showed an increase in Temra. We also found age-dependent Tnaive decrease and Temra, Treg Eff increases concordant with previous reports^78^ (Figure 3C). Sex differences in immunity are critical, especially for autoimmune diseases, because 80% of autoimmune disease occurs in females.^79^ Previous reports have addressed several changes in females, such as the increase of recent thymic emigrants^80^ and greater activation responses by *in vitro* stimulation.^81^ As for gender differences, we observed a decrease in Tcm (Th2), Tem (Th1/17), Temra (Th1), and Treg Eff and an increase in Tnaive Act, Tnaive *MX1*, Tnaive *SOX4*, Tem (Tph), and Treg Naive in females. These alterations depending on disease, gender, and age were also observed as specific distributions on the principal-component analysis plot (Figures 3D–3F, S9C, and S3D). Overall, we profiled numerical features of CD4^+^ T cells in broad autoimmune status, age, and gender.

Next, to measure the quality changes in autoimmune diseases, we applied NMFproj to the datasets and investigated NMF cell feature changes in each cluster L2 population (Figures 3G, S10A, and S11; Table S9). To add measurements for the biological significance, we also calculated a scaled intercept as the baseline gene program activity (Figure S11). The strongest skews were enriched in NMF7 (INF-F) in SLE and COVID-19 patients in a cell-type-wide manner. We also found that even neutral populations such as Tnaive, Tnaive (Act), and Tcm (Th0) showed disease-specific propensities. For example, NMF0 (Cytotoxic-F) increased in RA, MS, and pSS, NMF10 (Tissue-F) was increased in MS, COVID-19, SLE, and neurosarcoidosis, while NMF3 (Naive-F) decreased in a broad range of autoimmune diseases. In Treg cells, NMF1 (Treg-F) decreased in T1D, MG, and MS, indicating the dysfunction of Treg in these diseases independently of the number of Treg cells. In particular, for T1D and MS, where the sample size was large, we investigated the differentially expressed genes in each Treg subtype in each diseases and examined the genes with high feature values for NMF1 (Treg-F) (Figure S10B). Variations in the NMF1 (Treg-F)-related genes were most pronounced in Treg Eff, with T1D showing a decrease in functional molecules such as *IL2RA*, *TNFRSF1B*, and Treg regulators such as *FOXP3*, *IKZF2*, while MS showed a decrease in *IL2RA*, *IL10RA*, *IKZF4*, along with an increase in *CTLA4*, which is one of the prominent Treg effector molecules. Age-dependent increases of NMF8 (Cent. Mem.-F) and NMF4 (Act-F) were also observed. In females, NMF4 (Act-F) was enhanced broadly. Other findings, such as the upregulation of an activation molecule, CD69, which was the feature gene of NMF10 (Tissue-F), in MS and SLE,^82^ and the reduction of effector Tregs and their decreased function in T1D,^83^ are also consistent with our meta-analysis. In conclusion, by meta-analysis, in addition to quantitative changes, we identified qualitative changes depending on the disease, sex, and age at a resolution that is difficult to observe in existing methodologies.

### Predictive potential of peripheral CD4^+^ T profiles for autoimmune diseases

Given that each autoimmune disease disorder possessed a characteristic CD4^+^ T cell profile, we hypothesized that disease status might be predicted solely from CD4^+^ T profiles by utilizing machine learning techniques and our autoimmune-wide scRNA-seq dataset (Figure S12A). To confirm this, we created three models for the prediction of disease status. The first model took the frequency of each cell population in cluster L2, the second model took the cellular features of the NMF in subsets, and the last model took both as input parameters (Figure 3H). We note that, for NMFproj features, only Tnaive and Tcm (Th0) cell features, which were affected by various conditions as discussed previously (Figures 3G and S10A), were used to avoid over-fitting. First, binary classification models were constructed for SLE, COVID-19, and MS to distinguish between certain diseases and healthy individuals using logistic regression where equal numbers of diseased and healthy subjects were used for the training, and the models were evaluated using samples from independent projects from these used for the training (Figure 3I). SLE and COVID-19 yielded relatively good predictions from cell frequencies alone (area under the curve [AUC] = 0.84, 0.82 in SLE and COVID-19, respectively). NMF cell features alone also could predict SLE and COVID-19 well (AUC -= 0.94, 0.85 in SLE and COVID-19, respectively), and the perdition using both cell frequencies and NMFproj values further improved accuracy (AUC = 0.94, 0.91 in SLE and COVID-19 respectively). For MS, for which hematological biomarkers have not yet been well established, prediction from cell frequencies or from NMFproj values alone was not successful (AUC = 0.61, 0.47 in cell frequency and NMFproj, respectively), while both cell frequencies and NMFproj values resulted in better predictions (AUC = 0.75) than previous plasma based methods.^84^ Next, we built multiclass classification models that more closely resemble real-world clinical practice. The multiclass classification was assumed to be a more difficult task due to the similarities among autoimmune diseases and the imbalance of training sample sizes. We trained a gradient boosting model with 5-fold cross-validation on data from 714 samples from 8 diseases or healthy for which at least 10 samples were available for training and then evaluated the model using the data from the independent dataset (Figures S12C and S12D). The model trained only by cell frequencies or by NMFproj values could predict COVID-19, SLE, HC, and MS (area under the precision-recall curve [PR-AUC] = 0.72, 0.68, 0.48, 0.12 in the cell frequency model and 0.91, 0.75, 0.47, 0.35 in the NMFproj model, for COVID-19, SLE, HC, and MS, respectively), and the model trained by both cell frequencies and NMFproj values in Tnaive and Tcm (Th0) was marked with superior accuracy (PR-AUC = 0.92, 0.69, 050, 0.33 for COVID-19, SLE, HC, and MS, respectively). The combined cell frequency and NMF model also achieved the highest accuracy (the cell frequency model: 0.413, the NMF model: 0.484, and the cell frequency + NMF model: 0.527). These results highlight that the disease-specific changes in CD4^+^ T cells, both in terms of quantitative and qualitative alterations, contribute to the prediction of disease status.

### Partitioned heritability of autoimmune diseases on CD4^+^ T cell NMF features

**Figure 4:**
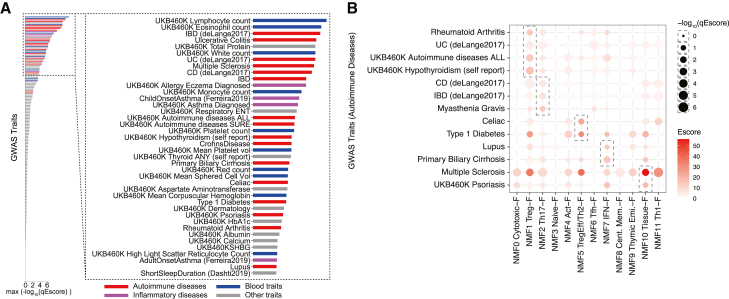
Partitioned heritability of autoimmune diseases by CD4^+^ T cell features (A) Bar plot showing maximum –log_10_(q_ *E* score) among NMF gene features. Partitioned heritability of GWASs was measured using the sc-linker framework. Enrichment of each category is the following: autoimmune diseases, p = 8.51 × 10^−12^; inflammatory traits, p = 0.131; and blood cell count, p = 7.59 × 10^−8^ (two-sided Mann-Whitney U test). (B) Dot plot showing enrichment of partitioned heritability of autoimmune diseases across NMF gene features. The dashed boxes indicate the factor with the highest *E* score for each disease. Duplicated traits were removed for the visualization. Full statistics are shown in Table S11.

We examined the association between CD4^+^ T characteristics and genetic factors for each disease and trait. Studies using GWAS statistics have reported associations between autoimmune diseases and immune cells.^2^^,^^85^ In particular, stratified linkage disequilibrium score regression (S-LDSC) has revealed the association between CD4^+^ T cells and autoimmune diseases by stratifying the heritability of polygenic autoimmune diseases by genetic features.^86^^,^^87^ Since we captured elaborate CD4^+^ T cell features, we investigated which of these features was associated with each trait using the S-LDSC framework. In addition to the cell-type-specific genes of cluster L2, 12-dimensional features extracted by NMF were used as genetic features. The Roadmap enhancer-gene linking (Roadmap) and activity-by-contact strategies introduced in the sc-linker framework^88^ were used for linking genes and SNPs. We note that as some gene programs were shared with other immune cells (Figure S6G), some associations below may potentially be influenced a broader range of cell types. Among 180 traits, autoimmune diseases showed significantly high enrichment in NMF features (p = 8.51 × 10^−12^), suggesting autoimmunity is closely associated with CD4^+^ T cells (Figure 4A). Cross-sectional disease association revealed that many diseases, such as inflammatory bowel disease (IBD), RA, and MG, have an enrichment of heritability on NMF1 (Treg-F) (Figure 4B). By focusing on the most accumulated factors for each disease, we found that autoimmune diseases can be divided into several groups. For each disease, the most enriched gene features were observed as NMF1 (Treg-F): RA, UC (deLange), hypothyroidism; NMF2 (Th17-F): CD (deLange), IBD (deLange), MG; NMF5 (TregEff/Th2-F): celiac disease, T1D; NMF7 (IFN-F): SLE, primary biliary cirrhosis; NMF10 (Tissue-F): MS, psoriasis. In MS, accumulation was observed in various features, including NMF2 (Th17-F) and NMF11 (Th1-F). The heritability of each autoimmune disease was accumulated in several factors, suggesting that autoimmune diseases have multiple susceptibilities. In other traits, weak accumulation on NMF1 (Treg-F), NMF2 (Th17-F), and NMF11 (Th1-F) was common in COVID-19 in both severe symptoms and infection, while NMF7 (IFN-F) was infection specific (Figure S13A). Lymphocyte counts were also susceptible to NMF4 (Act-F) (Figure S13A). We also examined enrichment in cell-type-specific genes. Similar enrichment patterns, such as Treg Naive in most autoimmune diseases and Tnaive *MX1* in SLE, were observed, while caution should be taken as marker gene detection is not optimal for CD4^+^ T cells as in the previous section (Figure S13B). Taken together, we comprehensively profiled heritability enrichment on CD4^+^ T cell gene features across autoimmune diseases.

### Partitioned heritability is associated with qualitative and/or quantitative changes in CD4^+^ T in a disease-specific manner

**Figure 5:**
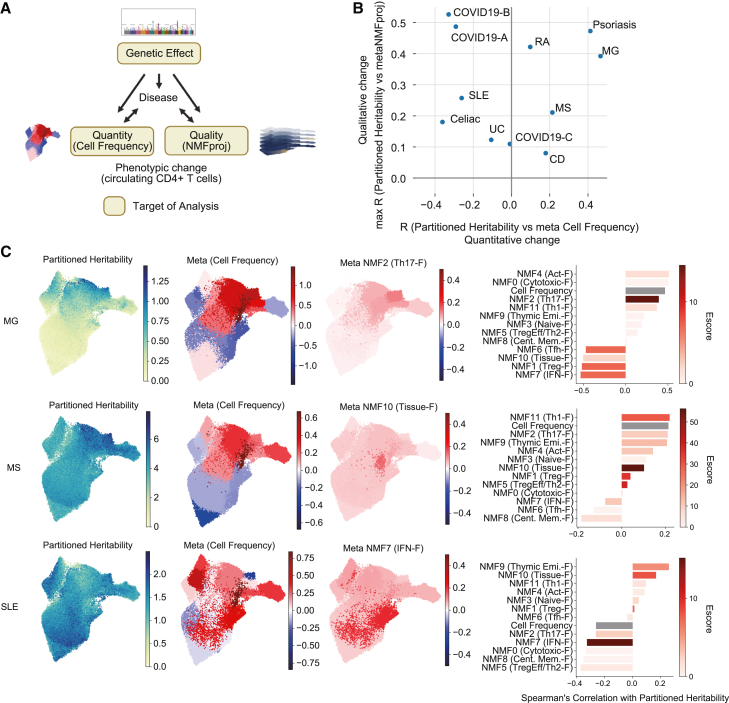
Relationship between genetic factors and phenotypic changes in CD4^+^ T cells (A) Model of genetic effect on phenotypic changes in CD4^+^ T cells. CD4^+^ T cell changes are observed as qualitative (NMFproj cell features) and quantitative (cell-type frequencies) changes. (B) Scatterplot showing the genetic effect on cell frequencies (x axis) and NMF features (y axis). Sc-linker weight per cell was calculated by dot products of sc-linker outcome (NMF) and NMF cell features. For cell frequencies and NMF cell features, coefficients of GLM output for each cluster L2 population were used. Spearman’s correlation of sc-linker weight and cell frequency/NMF cell feature changes were calculated. For the correlation with sc-linker and NMF cell feature changes, we used the maximum R among NMF features for the visualization. COVID19-A, very severe respiratory symptom; COVID19-B, hospitalized; COVID19-C, SARS-CoV-2 infection. (C) Individual sc-linker weights, cell frequency changes (coefficient for each cluster L2), and NMF cell feature changes in the factor with the highest magnitude (*E* score) (coefficient for each cluster L2) of MS, MG, and SLE were visualized on the UMAP embeddings (left panel). For the coefficient of the NMF cell feature changes, only one representative factor with the highest *E* score for each disease is shown. The bar plot of Spearman’s correlation of cell frequency and NMFproj changes with partitioned heritability is shown in the right panel. The colors of the bars, except for cell frequency, indicate the *E* score calculated using sc-linker.

Finally, we compared partitioned heritability and observed changes in CD4^+^ T in terms of quantity (cell frequency) and quality (NMFproj) to assess the genetic effect on phenotypic changes (Figure 5A). We first investigated the correlation between enriched heritability and changes in cell frequency and NMF features for diseases that were enrolled in our analysis for both GWASs and meta-analysis. We found several patterns depending on the disease (Figure 5B). First, in MG and psoriasis, both changes in cell frequency and NMF feature correlated with heritability accumulation. In severe COVID-19 (COVID19-A, COVID19-B) and RA, the correlation was mainly observed with changes in quality only. SLE, celiac disease, UC, and SARS-CoV-2 infection (COVID19-C) showed poor correlation with cell frequency.

Next, we examined MS, MG, and SLE, whose samples were collected in this study, in more detail (Figure 5C). In MG, NMF2 (Th17-F), which accumulated heritability most significantly, was increased in Tcm (Th17) and Tem (Th1/17) in correlation with genetic factor accumulation, and the cell frequency was also correlated with genetic factors (Figures 5C and S13). On the other hand, NMF1 (Treg-F), which showed the next highest accumulation of heritability, was negatively correlated with the genetic effect and lower in Treg cells (Figure S13), indicating that the dysfunction of Treg cells might be enhanced by the genetic effect. In MS, the highest heritability accumulation was observed in NMF10 (Tissue-F). This factor was increased in all cell populations without cell specificity, resulting in a low correlation with the heritability. SLE susceptibility was most accumulated in NMF7 (IFN-F). In our meta-analysis, an enhancement of NMF7 (IFN-F) was observed in all cell populations, especially in Tnaive *MX1*. Overall, our study cataloged heritability enrichment and phenotypic changes across autoimmune diseases, enabling elucidation of the disease-specific effect of underlying genetic factors on CD4^+^ T cell phenotypes.

## Discussion

The classifications and characterizations of CD4^+^ T cells have been challenging, with cellular heterogeneity being a major obstacle.^4^ In this study, by performing single-cell analysis on CD4^+^ T cells from autoimmune and healthy subjects, we succeeded in mutually exclusive and collectively exhaustive subtype identifications of peripheral CD4^+^ T cells. Moreover, in contrast to the conventional dualistic comparisons, such as Th1 vs. Th2 and Treg vs. Th17, the NMF-based decomposition revealed that CD4^+^ T cells are formed by a combination of 12 features rather than simple contradistinction. While qualitative profiling by NMF was not suitable for numerical evaluation, it allowed for a more robust assessment of gradual cell populations. Moreover, our results can also be extrapolated for other single-cell and bulk RNA-seq studies by using a label transfer and the projection of NMF features.

These analytical frameworks also allowed us to perform autoimmune-wide single-cell meta-analyses and integration of CD4^+^ T cell features with GWASs. As a result, we comprehensively cataloged CD4^+^ T cell alterations in 20 diseases, providing a valuable resource for a broad range of disease research. The assessment of qualitative changes through NMFproj enabled us to explore biological insights, such as Treg functional abnormalities, that were previously unattainable using cytometry. Furthermore, the decomposition of gene programs using NMF was beneficial not only for T cell profiling but also for interpreting GWAS results. We found that genetic factors can have both disease-specific and cross-disease impacts on autoimmune conditions. The accumulation of heritability on Tregs across diseases and the disease specificity of other features may be potential clues for future therapeutic development.

By examining genetic factors, CD4^+^ T cell changes, and TCR characteristics in a disease-specific manner, we gained valuable insights into various diseases. For example, MG is caused by autoantibodies against the neuromuscular junction, with germinal center responses involving Tfh cells and B cells within the thymus.^7^^,^^89^ While Th17 function enhancement has been reported in MG,^73^^,^^90^ we also observed a heritability enrichment in NMF2 (Th17-F), suggesting that tissue damage by Th17 cells may contribute to symptom completion and persistence. In MS, we observed an increase in NMF10 (Tissue-F) and heritability enrichment in addition to the previously known Th17 and Th1 increase and functional enhancement.^74^^,^^75^^,^^91^ These results suggest that a strong tissue inflammatory response is involved in MS and emphasizes that the tissue-specific gene program centered on the AP-1 family may be a novel MS-specific therapeutic target.^92^ In addition, in MS, while Treg Eff slightly increased, the quality of Treg cells in terms of transcriptome and TCR was low, indicating that the compensation of Tregs from Tconvs is an explanation for Treg dysfunction in MS.^93^ In SLE, our analysis of heritability enrichment and qualitative alterations supports the traditional belief that type I IFN is central to the disease.^94^ Type I IFN drives differentiation into Tregs and Th1 cells,^95^^,^^96^ and our result suggests that the pleiotropic effect of type I IFN contributed to the complicated cell frequency changes observed in this study. Furthermore, TCR overlaps between Tcm (Tfh) and Treg Act were observed specifically in SLE, suggesting potential Treg-Tfh plasticity in SLE similar to reported Tfh-Treg plasticity under certain inflammatory conditions.^97^^,^^98^ We identified distinct CD4^+^ T cell responses between COVID-19 and influenza infection, with an increase in Tnaive cells in COVID-19 and Temra cells in flu. This divergence may reflect differences between pre-trained immunity to influenza and initial responses to SARS-CoV-2, as most COVID-19 samples were collected before the vaccine rollout. In addition, our meta-analysis revealed sex- and age-related CD4^+^ T cell changes, with new observations such as increased Tnaive *MX1* and Tem (Tph) in females, potentially contributing to gender differences in autoimmune disease incidence. Thus, our study highlights the CD4^+^ T cell features of each disease and condition, providing new insights for consideration.

In addition, this study created a comprehensive single-cell circulating CD4^+^ T cell catalog across various diseases, providing the opportunity to tackle the challenging task of assessing whether disease prediction is feasible using CD4^+^ T cell profiles. Our framework offers an approach to measuring CD4^+^ T profiles from limited cell counts or sparse gene expression data through the pre-defined reference and NMFproj. However, the predictive accuracy of the machine learning model utilizing CD4^+^ T profiles in this study was still limited, which could be attributed to factors such as limited sample sizes, sample imbalance, and variations between cohorts. Nevertheless, this underscores the potential for predicting disease status using these profiles. Moreover, the distinct variations in CD4^+^ T profiles across diseases emphasize the necessity for individualized treatments tailored to specific conditions.

In summary, we constructed the frameworks for extracting the CD4^+^ T cell programs, enabling a comprehensive interpretation of CD4^+^ T cells. Moreover, the landscape of disease-specific CD4^+^ T cell alterations and genetic effects provides biological insights for potential precision medicine.

### Limitations of the study

There are several limitations worth noting. Firstly, since our meta-analysis contained data from not only whole PBMCs but also pre-enriched CD4^+^ T cell samples, we could not consider the proportional alterations of CD4^+^ T cells in overall PBMCs. Furthermore, our study focused on peripheral blood and did not evaluate tissue-specific alterations, such as barrier tissue-specific programs.^99^ This might be the reason that we could not capture some known vital phenomena for CD4^+^ T cells, such as anergy and exhaustion. For example, when we compared with *ab initio* NMF for TILs, while the factors defined in blood were largely preserved CD4^+^ T cells in tissues, it is worth noting that certain factors, including those encompassing *CXCL13* and *IL21*, were defined only in TILs, emphasizing the importance of analyses using tissue samples in the future.

In addition, while our study provides insights into the relationship between gene programs in CD4^+^ T cells and autoimmune diseases, further experimental validation such as interventions in genes and environments *in vitro* could offer more precise gene targeting and therapeutic applications in real-world contexts. Integrating these methodologies with our findings and using NMFproj for transcriptome evaluations might accelerate research for autoimmune diseases and cellular manipulations.

## STAR★Methods

### Key resources table

**Table:** 

REAGENT or RESOURCE	SOURCE	IDENTIFIER
**Antibodies**		

Fc Receptor Binding Inhibitor Polyclonal Antibody, Functional Grade, eBioscience™	Thermo Fisher Scientific	Cat#16-9161-73; RRID: AB_469272
BD Pharmingen™ FITC Mouse Anti-Human CD3	BD Bioscience	Cat#561806; RRID: AB_11154397
A CD4 Monoclonal Antibody (RPA-T4), APC, eBioscience™	Thermo Fisher Scientific	Cat#17-0049-42; RRID: AB_1272048
PE anti-human CD19 Antibody	BioLegend	Cat#302207; RRID: AB_314237

**Chemicals, peptides, and recombinant proteins**		

Ficoll-Paque Plus	Cytiva	Cat#17144003
LIVE/DEAD Fixable Near-IR Dead Cell Stain Kit	Thermo Fisher Scientific	Cat#L34975

**Critical commercial assays**		

Chromium Next GEM Chip K	10x Genomics	PN-1000286
Chromium Next GEM Single Cell 5′ Kit v2	10x Genomics	PN-1000263
Chromium Single Cell Human TCR Amplification Kit	10x Genomics	PN-1000252

**Deposited data**		

scRNA-seq data	This paper	JGA: JGAS000578 https://singlecell.broadinstitute.org/single_cell/study/SCP1963
scRNA-seq data for MG	Yasumizu et al.^7^	JGA: JGAS000482
ImmuNexUT	Ota et al.^39^	GEA: E-GEAD-397
European LD scores	Jagadeesh et al.^88^	https://storage.googleapis.com/broad-alkesgroup-public/LDSCORE/Dey_Enhancer_MasterReg/processed_data,
Roadmap_U_ABC for blood	Jagadeesh et al.^88^	https://storage.googleapis.com/broad-alkesgroup-public/LDSCORE/DeepLearning/Dey_DeepBoost_Imperio/data_extra/AllPredictions.AvgHiC.ABC0.015.minus150.withcolnames.ForABCPaper.txt.gz
scCITE-seq data	Hao et al.^62^	GEO: GSE164378
public scRNA-seq data	See Table S6	See Table S6

**Software and algorithms**		

Code and algorithms for analysis	This paper	https://github.com/yyoshiaki/autoimmune10x_CD4T_Manuscript_2023 (https://doi.org/10.5281/zenodo.10154467) https://github.com/yyoshiaki/sclinker-skg (https://doi.org/10.5281/zenodo.10200443)
NMFproj	This paper	https://github.com/yyoshiaki/NMFprojection (https://doi.org/10.5281/zenodo.10200434)
Pipeline for single-cell reference mapping used in meta-analysis	This paper	https://github.com/yyoshiaki/screfmapping (https://doi.org/10.5281/zenodo.10200424)
Cell Ranger	10x Genomics	v4.0.0
Scanpy	Wolf et al.^100^	https://scanpy.readthedocs.io/en/stable/
Harmony	Korsunsky et al.^13^	https://github.com/immunogenomics/harmony
scikit-learn	Pedregosa et al.^101^	https://zenodo.org/records/10039710
ikra	Hiraoka et al.^102^	https://zenodo.org/records/3352573
clusterProfiler	Yu et al.^103^	https://github.com/YuLab-SMU/clusterProfiler
ReactomePA	Yu et al.^104^	https://github.com/YuLab-SMU/ReactomePA
scirpy	Sturm et al.^105^	https://github.com/scverse/scirpy
TiRP	Lagattuta et al.^28^	https://github.com/immunogenomics/TiRP
S-LDSC	Bulik-Sullivan et al.^106^	https://github.com/bulik/ldsc
CellTypist	Domínguez Conde et al.^36^	https://github.com/Teichlab/celltypist
projecTILs	Andreatta et al.^16^	https://github.com/carmonalab/ProjecTILs
Azimuth	Hao et al.^62^	https://azimuth.hubmapconsortium.org/
Seurat	Hao et al.^62^	https://github.com/satijalab/seurat
Symphony	Kang et al.^14^	https://github.com/immunogenomics/symphony
pyCirclize	N/A	https://github.com/moshi4/pyCirclize
imbalanced-learn	Lemaitre et al.^107^	https://github.com/scikit-learn-contrib/imbalanced-learn
LightGBM	Ke et al.^108^	https://github.com/Microsoft/LightGBM
statsmodels	Seabold et al.^109^	https://github.com/statsmodels/statsmodels

### Resource availability

#### Lead contact

Further information and requests for resources and reagents should be directed to and will be fulfilled by the lead contacts, Naganari Ohkura (nohkura@ifrec.osaka-u.ac.jp).

#### Materials availability

This study did not generate new unique reagents.

#### Data and code availability

• The raw sequence data for single-cell RNA-seq analysis have been deposited at Japanese Genotype-phenotype Archive (JGA) under accession number JGAS000482 (https://ddbj.nig.ac.jp/resource/jga-study/JGAS000482) and JGAS000578 (https://ddbj.nig.ac.jp/resource/jga-study/JGAS000578). Single-cell data can be explored interactively and downloaded in SingleCellPortal (https://singlecell.broadinstitute.org/single_cell/study/SCP1963). Detailed results of the meta-analysis and sc-linker were deposited at https://yyoshiaki.github.io/autoimmune_scRNAseq/Tcells.html and https://doi.org/10.6084/m9.figshare.23983038.
• All original code is deposited at https://doi.org/10.5281/zenodo.10154467 and in the GitHub repository https://github.com/yyoshiaki/autoimmune10x_CD4T_Manuscript_2023 (https://doi.org/10.5281/zenodo.10154467) and https://github.com/yyoshiaki/sclinker-skg (https://doi.org/10.5281/zenodo.10200443). NMFproj is provided at https://github.com/yyoshiaki/NMFprojection (https://doi.org/10.5281/zenodo.10200434). The pipeline for reference mapping used in meta-analysis is provided at https://github.com/yyoshiaki/screfmapping (https://doi.org/10.5281/zenodo.10200424)
• Any additional information required to reanalyze the data reported in this paper is available from the lead contacts upon request.

### Experimental model and study participant details

#### Human samples

The study using human samples was reviewed and approved by the Research Ethics Committee of Osaka University and carried out in accordance with the guidelines and regulations. Human samples were collected under approved Osaka University’s review board protocols: ID 708-10. Written informed consent was obtained from all donors. Patient information is provided as a supplementary table (Table S1).

### Method details

#### Cell preparation and sequencing of scRNA-seq

From blood collected using heparin-coated tubes, we first collected PBMCs using Ficoll-Paque Plus (Cytiva). PBMCs were washed, blocked with Fc Receptor Binding Inhibitor Polyclonal Antibody, Functional Grade, eBioscience (Thermo Fisher Scientific), and stained with FITC-labeled anti-CD3 mAb (dilution: 1/100, UCHT1, BD Bioscience), APC-labeled anti-CD4 mAb (dilution: 1/100, RPA-T4, Thermo Fisher Scientific), PE-labeled anti-CD19 mAb (HIB19, BioLegend), Live/Dead (Thermo Fisher Scientific). Live-CD3^+^CD4^+^CD19^−^ cells were isolated using BD Biosciences FACS Aria II or BD Biosciences FACS Aria III. CD4^+^ T cells and B cells were mixed in equal numbers in some samples.

The sorted cells were loaded to Chromium Next GEM Chip K (10x Genomics) on Chromium Controller (10x Genomics) for barcoding and cDNA synthesis. The library construction was performed using Chromium Next GEM Single Cell 5′ Kit v2 and Chromium Single Cell Human TCR Amplification Kit (10x Genomics) for 5′ according to the manufacturer’s protocol. The libraries were sequenced on NovaSeq6000 (Illumina).

#### Preprocess of scRNA-seq data

Sequenced reads were processed using Cell Ranger (v4.0.0) with pre-built reference refdata-gex-GRCh38-2020-A and refdata-cellranger-vdj-GRCh38-alts-ensembl-4.0.0 downloaded at 10x GENOMICS' Website. Quantified expressions were preprocessed and visualized using Scanpy 1.8.1^100^ and Python 3.8.0. For CD4^+^ T cell and B cell mixed samples, we extracted only CD4^+^ T cells as following procedures. Briefly, we normalized (sc.pp.normalize_total) gene expression, log-transformed it (sc.pp.log1p), extracted highly variable genes (HVGs) (sc.pp.highly_variable_genes with min_mean = 0.0125, max_mean = 3, min_disp = 0.5), computed PCA (sc.tl.pca) and neighbors (sc.pl.neighbors with n_neighbors = 10, n_pcs = 40), computed clusters using Leiden algorithm (sc.tl.leiden), and embedded using UMAP algorithm (sc.tl.umap). CD3E-positive and MS4A1-negative clusters were extracted as CD4^+^ T cells and used for the analysis. Cells with mitochondrial genes were higher than 10%, detected genes less than 200, or annotated as multichain by scirpy were filtered out. Variable genes of TCR alpha and beta were removed for the clustering and embedding to remove the effect of clonal expansion. Gene expressions were preprocessed by sc.pp.normalize_per_cell with counts_per_cell_after = 1e4, sc.pp.log1pp, retained HVGs. The inference of the cell cycle was performed using the sc.tl.score_genes_cell_cycle function following the tutorial (https://nbviewer.jupyter.org/github/theislab/scanpy_usage/blob/master/180209_cell_cycle/cell_cycle.ipynb). Total counts of UMI, % mitochondrial genes, S score, G2M score were regressed out using sc.tl.regress_out and scaled using sc.tl.scale. Then, principal components were computed using sc.tl.pca. The batch effect of samples was removed by the Harmony algorithm.^13^ Cells were embedded by UMAP using sc.tl.umap (spread = 1.5), and clustered using sc.tl.leiden (resolution = 1.2). Re-clustering and embedding were performed using sc.tl.umap (spread = 1.5), clustered using sc.tl.leiden (resolution = 1.7) after removing clusters containing doublets with B cells, monocyte lineages, etc. We defined cluster L1 as a large classification using the leiden clusters. Next, for some clusters, concatenation or re-clustering was performed with sc.tl.leiden (resolution 0.3–1) to divide clusters at the minimum resolution with distinct marker genes. We defined cluster L2 as a smaller classification. Marker genes were determined using sc.tl.rank_genes_groups with method = ’t-test_overestim_var’. The silhouette scores were calculated using the silhouette_samples function from scikit-learn (v0.24.2) with the "euclidean" metric.

#### Integration with bulk RNA-seq dataset

Fastq files were processed using an RNA-seq integrative pipeline, ikra (v2.0.1),^102^ composed of Trim Galore! 0.6.7,^110^ Salmon 1.4.0,^111^ tximport 1.6.0^112^ with the reference Gencode M26 for mice and 37 for humans. Datasets for which the TPM matrix was provided were downloaded and used directly for analyses. For the ImmuNexUT (E-GEAD-397) dataset, the downloaded count matrix was converted to TPM.

For the correlations between bulk RNA-seq and scRNA-seq datasets, TPM or scaledTPM expression matrix of bulk RNA-seq were normalized using sc.pp.normalize_per_cell (counts_per_cell_after = 1e4), sc.pp.log1p, sc.pp.scale (max_value = 10), concatenated to scRNA-seq object, and calculated the correlations using sc.tl.dendrogram with the default parameters.

#### Gene expression decomposition using NMF

To decompose cellular processes, we applied NMF implemented in scikit-learn (v0.24.2) to normalize gene expression of HVGs. Using the non-negative matrix factorization (NMF) method, the normalized gene expression matrix X, with elements represented as $x_{ij}$, was decomposed into a gene feature matrix W (with elements represented as $w_{ic}$) and a cell feature matrix H (with elements represented as $h_{cj}$). In this context, i refers to the gene index, j represents the cell index, and c denotes the component. The decomposition is given by:

X=W⋅H

For our analysis, the number of components was determined to be 12 based on two criteria: i) it exceeded the elbow in the distribution of explained variance and ii) it was just before a sharp increase in the maximum inter-components Spearman’s correlation. The explained variance for the given number of components c is denoted as ${Evar}_{c}$ and is derived using the residual sum of squares (RSS) for component c, represented as ${RSS}_{c}$ :

$${RSS}_{c}=\sum_{ij}{\left(x_{ij}-w_{ic}h_{cj}\right)}^{2}$$

The explained variance ${Evar}_{c}$ is then:

$${Evar}_{c}=1-\frac{{RSS}_{c}}{\sum_{ij}x_{ij}^{2}}$$

Additionally, ${Evar}_{all}$, which represents the overall explained variance across all components, is computed from the residuals of X and W⋅H, denoted as ${RSS}_{all}$:

$${RSS}_{all}=\sum_{ij}{\left(x_{ij}-w_{i\cdot }h_{\cdot j}\right)}^{2}$$

$${Evar}_{all}=1-\frac{{RSS}_{all}}{\sum_{ij}x_{ij}^{2}}$$

For the pathway enrichment analysis, we extracted the top 100 genes with the highest feature value for each component and converted gene symbols into Entrezid using the bitr function provided by clusterProfiler (3.16.1)^103^ and computed enriched Reactome pathways using the compareCluster function of clusterProfiler with the enrichPathway function in ReactomePA (1.32.0). For the projection of gene features defined by NMF, we performed NMF with pre-computed W using scikit-learn. The matrix W was converted to mouse genes using a list of human and mouse homologs provided at http://www.informatics.jax.org/homology.shtml. For genes with multiple homologs, one of the genes was retained. For the NMF calculation, only overlapped genes were used. To examine whether the selected HVGs of fixed W can capture HVGs in a query dataset, we calculated the proportion of the number of HVGs included in fixed W against the number of HVGs in the query dataset as POH. In the CD4^+^ T dataset, we determined that if the POH is below 0.1, which is the conservative threshold from the distribution of the null hypothesis (Figure S3C), there is a variance that cannot be represented by the NMFproj. sc.pp.highly_variable_genes in scanpy with the following parameters; min_mean = 0.0125, max_mean = 3, min_disp = 0.1 was used for the calculation of HVGs of the query datasets and selected top 500 genes regarding normalized dispersion^113^ with the exclusion of VDJ genes of TCR and IG. A framework for NMF projection is available at https://github.com/yyoshiaki/NMFprojection.

For the analysis of ImmuNexUT, the count data was downloaded at (https://humandbs.biosciencedbc.jp/en/hum0214-v3) and converted to TPM. We removed BD, AAV, AOSD, and SjS samples from the analysis because these samples are processed in a different procedure from other samples. TPM matrix was decomposed with the pre-computed gene feature matrix using NMFproj. Extracted NMF feature H was tested using a multiple linear regression provided as the formula.api.ols function by a python package statsmodels (0.12.0) with a model, NMF_i ∼ Disease + Age + Gender +1.

#### TCR analysis

For TCR analysis, we used the standard pipeline of scirpy 0.10.1^105^ according to the official tutorial (https://scverse.org/scirpy/latest/tutorials/tutorial_3k_tcr.html). Briefly, clones were defined by clonotypes using scirpy.tl.define_clonotypes with parameters; receptor_arms = "all", dual_ir = "primary_only". Repertoire similarities were measured using the function scirpy.tl.repatoire_overlap. TiRP score was calculated according to the instruction in the repository (https://github.com/immunogenomics/TiRP.git).

#### Heritability partitioning

To assess the contribution of each cell-type-specific gene expression and the NMF component, we applied S-LDSC with Roadmap ABC-immune enhancer-gene linking strategy implemented using an sc-linker pipeline^88^ with slight modifications (https://github.com/yyoshiaki/sclinker-skg). We only used HVGs defined by the preprocessing section in the analysis. We used min-max scaled gene scores for the cell-type gene programs as the gene weights. For NMF components, we used the gene feature matrix W with the min-max scaling for the gene weights. Using the gene weights, LD scores for each category were calculated with European LD scores used in the article^88^ and Roadmap_U_ABC for blood (https://storage.googleapis.com/broad-alkesgroup-public/LDSCORE/Dey_Enhancer_MasterReg/processed_data, https://storage.googleapis.com/broad-alkesgroup-public/LDSCORE/DeepLearning/Dey_DeepBoost_Imperio/data_extra/AllPredictions.AvgHiC.ABC0.015.minus150.withcolnames.ForABCPaper.txt.gz). In addition to the sumstats files provided in gs://broad-alkesgroup-public/LDSCORE/all_sumstats, we used several additional sumstats by processing using munge_sumstats.py in LDSC v1.0.1 (Table S10). S-LDSC was performed with the baseline-LD model v2.1 (https://storage.googleapis.com/broad-alkesgroup-public/LDSCORE/1000G_Phase3_baselineLD_v2.1_ldscores.tgz). The Enrichment score (*E*-score) was calculated as the difference between the enrichment for annotation in a particular program against an SNP annotation for all protein-coding genes with a predicted enhancer-gene link in the blood. We also used FDR calculated from the p value of Enrichment outputted by S-LDSC.

#### Comparison of our ref-mapping classification performance with other classification tools

To compare the performance of our ref-mapping with other tools, we applied various cell classifiers to the same dataset. For this purpose, we utilized the pre-annotated scCITE-seq data generated by Hao et al.^62^ on PBMC and extracted circulating CD4^+^ T cells. The procedures for the reference mapping by Symphony using our reference were described in the subsequent meta-analysis section. For CellTypist,^36^ following the tutorial (https://www.celltypist.org/tutorials), we normalized gene expression (total count = 10000) and log-transformed it and then applied CellTypist. For the CellTypist model, we used the model built on the human PBMC data (COVID-19_HumanChallenge_Blood). Regarding projecTILs,^16^ the dataset was normalized gene expression count (total count = 10000), log-transformed, and performed labal transfer following the tutorial (https://github.com/carmonalab/ProjecTILs). For the transfer, we used references built with human tumor-infiltrating CD4^+^ T cells (CD4T_human_ref_v1.rds).

#### Meta-analysis of CD4^+^ T cells from public datasets

We collected scRNA-seq data from PBMC generated by 10x platforms, Seq-Well or SPLiT-seq (Parse Biosciences WT Mega)^6^^,^^8^^,^^40^^,^^41^^,^^42^^,^^43^^,^^44^^,^^45^^,^^46^^,^^47^^,^^48^^,^^49^^,^^50^^,^^51^^,^^52^^,^^53^^,^^54^^,^^55^^,^^56^^,^^57^^,^^58^^,^^59^^,^^60^^,^^61^ (Table S6). If the count matrix was available, we used the quantified matrix. Otherwise, we quantified the expression using Cell Ranger with pre-built reference refdata-gex-GRCh38-2020-A. As the sample QC, samples with XIST mean expression (count) > 0.05 were inferred as female. If the inferred gender and metadata differed, we removed the sample from the analysis. We extracted CD4^+^ T cells from published data of PBMCs using Azimuth 0.4.4.^62^ We created Seurat Object using the CreateSeuratObject function implemented in Seurat 4.1.0^62^ with parameters min.cells = 3, min.features = 200. We also filtered out the cells that express≧10% mitochondrial genes in their total gene expression. We normalized the expression using SCTransform with parameters method = "glmGamPoi", ncells = 2000, n_genes = 2000, do.correct.umi = FALSE. In this procedure, we used Azimuth reference data v1.0.0 human_pbmc loaded from the website (https://seurat.nygenome.org/azimuth/references/v1.0.0/human_pbmc). We found anchors between query data and Azimuth reference data (FindTransferAnchors with parameters k.filter = NA, normalization.method = "SCT", dims = 1:50, n.trees = 20, mapping.score.k = 100), transferred cell type labels (TransferData with parameters dims = 1:50, n.trees = 20) and calculated the embeddings on the reference supervised PCA (IntegrateEmbeddings with the default options) and neighbors (FindNeighbors with parameter l2.norm = TRUE). We transformed an NN index (NNTransform with the default parameters) and projected the query data to the reference UMAP (RunUMAP with the default parameters). We visualized query data by DimPlot, DotPlot, and FeaturePlot.

Next, we mapped extracted cells on our reference using symphony 0.1.0^14^ following the vignettes (https://github.com/immunogenomics/symphony/blob/main/vignettes/Seurat.ipynb). First, we created a symphony reference using our dataset. Our scanpy object saved as an h5ad file was converted to h5Seurat using SeuratDisk and loaded as a Seurat object. We used only HVGs for symphony reference to reduce batch effect strictly. Then, the object was preprocessed as follows; SCTransform (method = "glmGamPoi"), ScaleData, RunPCA, RunHarmony.Seurat (group.by = "sample"), FindNeighbors (dims = 1:30), RunUMAP2, and buildReferenceFromSeurat. For query mapping, extracted CD4^+^ T cells were normalized (SCTransform with parameter method = "glmGamPoi") and mapped (mapQuery with parameter do_normalize = FALSE, vars = "batch") with batch correction against each sample. The cluster L1 and L2 assignments were performed using the knnPredict.Seurat function. We visualized the mapping results by DimPlot and FeaturePlot as the quality control. We are providing the label transfer pipeline at https://github.com/yyoshiaki/screfmapping.

In the analysis, we performed a generalized linear model (GLM) independently for each subtype in cluster L2. For each cell population in a given donor, we define *n_cat* as the number of cells in that population, and *n_total* as the total number of cells in that donor. The response variable for the binomial regression is thus composed of *n_cat* and *n_total* - *n_cat*, representing the number of cells in the specific population and the number not in the population, respectively. The *Disease* variable was one-hot encoded where healthy was set as the baseline. The *Age* variable was scaled by dividing by 25 to match the scale of the other variables in the model. Therefore, the binomial regression was performed with the formula: (*n_cat*, *n_total* - *n_cat*) ∼ *Disease* + *Age*/25 + *Gender* + *Project* using the R glm function. This model allows us to assess the effects of disease, age, and gender on the frequency of each cell subtype within a given donor. Similarly, for the analysis of enriched NMF components, we first calculated cell profiles using NMFproj with raw counts, and linear regression was performed for each NMF component in each cell type respectively with the formula; *NMF_i* ∼ *Disease* + *Age*/25 + *Gender* + *Project* using the R glm function. The Chord diagrams were created using pyCirclize (0.1.3). For the PCA plot of individuals, principal components were calculated using R prcomp function (scale = TRUE) against cell frequencies of each sample. PC loadings for cell subsets were visualized using R biplot function.

#### Machine learning for the prediction of autoimmune states

To address potential study-specific effects, we ensured a clear division in our datasets: projects used as the training set, while independent projects, with no overlaps, were assigned as the test set. This guarantees the classifier’s evaluation on genuinely unseen data from a separate study. Cell frequencies and/or NMFproj values in Tcm (Th0) and Tnaive were scaled using StandardScaler (scikit-learn 1.0.2). Note that Tnaive and Tcm (Th0), which have a high degree of nodes in the network, were selected for the NMFproj results to keep the number of parameters low. NMF values were imputed using SimpleImputer (scikit-learn 1.0.2) with parameter strategy = 'most_frequent' trained by training datasets. For the binary classification, we used the LogisticRegression in scikit-learn with the default parameters. For the multiclass classification, the label imbalance was corrected using SMOTE (imbalanced-learn 0.9.1) with parameter sampling_strategy = 'all'. Then, LightGBM 3.3.2^108^ was used for the model with parameters, 'objective' = 'multiclass' and 'early_stopping_rounds' = 10.

### Quantification and statistical analysis

All statistical analyses were performed in R (4.0.3 or 4.1.2) and Python (3.8.0). FDR was obtained by the Benjamini-Hochberg procedure implemented by a Python package statsmodels (0.12.0). All other statistical analyses are detailed in the respective sections of the article.

## Supplemental information

Document S1. Figures S1–S13 and Table S1

Table S2. Top 100 marker genes for cluster L1, related to Figure 1

Table S3. Top 100 marker genes for cluster L2, related to Figure 1

Table S4. Top 100 feature genes for each NMF factor, related to Figure 2

Table S5. Top 50 enriched Reactome pathways for each NMF factor, related to Figure 2

Table S6. Publication list for single-cell meta-analysis, related to Figure 3

Table S7. The associations of traits and cluster L1 cell frequencies, related to Figure 3

Table S8. The associations of traits and cluster L2 cell frequencies, related to Figure 3

Table S9. The associations of traits and NMF features, related to Figure 3

Table S10. GWAS list used for sc-linker analysis, related to Figure 4

Table S11. The detailed results of sc-linker for NMF features, related to Figure 4

Table S12. The detailed results of sc-linker for cell-type-specific genes, related to Figure 4

Document S2. Supplementary Note

Document S3. Article plus supplemental information
