# Supplementary material for: Single-cell transcriptome landscape of circulating CD4+ T cell populations in autoimmune diseases
Source: Cell Genom. 2024 Jan 3;4(2):100473. doi: 10.1016/j.xgen.2023.100473 (PMC10879034; doi:10.1016/j.xgen.2023.100473)
Supplement: Document S1. Figures S1–S13 and Table S1 [file mmc1.pdf]

Cell Genomics, Volume *4*

Supplemental information

Single-cell transcriptome landscape of circulating $CD4^+$ T cell populations in autoimmune diseases

Yoshiaki Yasumizu, Daiki Takeuchi, Reo Morimoto, Yusuke Takeshima, Tatsusada Okuno, Makoto Kinoshita, Takayoshi Morita, Yasuhiro Kato, Min Wang, Daisuke Motooka, Daisuke Okuzaki, Yamami Nakamura, Norihisa Mikami, Masaya Arai, Xuan Zhang, Atsushi Kumanogoh, Hideki Mochizuki, Naganari Ohkura, and Shimon Sakaguchi

Figure S1

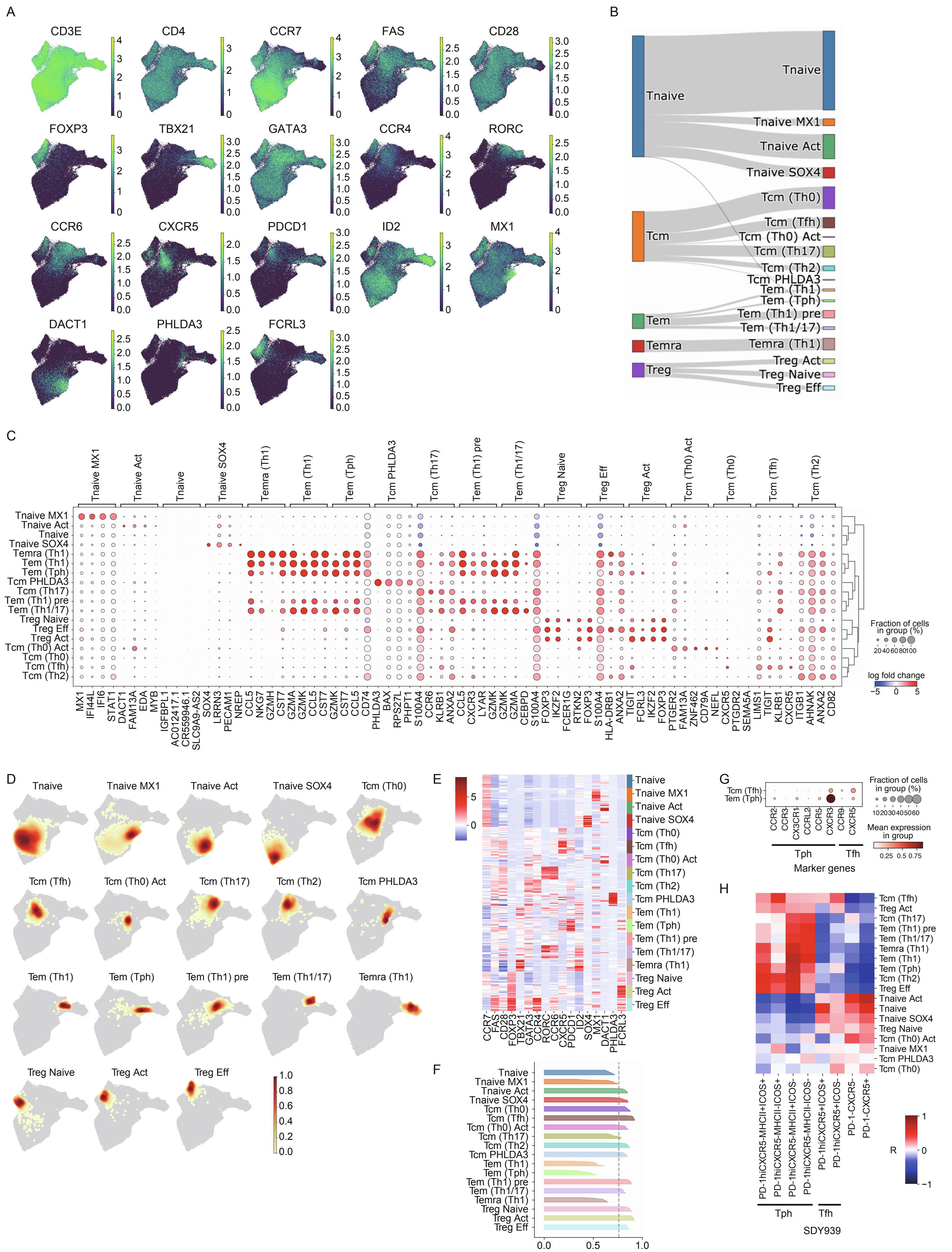

A
CD3E
CD4
CCR7
FAS
CD28
FOXP3
TBX21
GATA3
CCR4
RORC
CCR6
CXCR5
PDCD1
ID2
MX1
DACT1
PHLDA3
FCRL3
B
Tnaive
Tcm
Tem
Temra
Treg
Tnaive MX1
Tnaive Act
Tnaive SOX4
Tcm (Th0)
Tcm (Tfh)
Tcm (Th0) Act
Tcm (Th17)
Tcm (Th2)
Tcm PHLDA3
Tem (Th1)
Tem (Tph)
Tem (Th1) pre
Tem (Th1/17)
Temra (Th1)
Treg Act
Treg Naive
Treg Eff
C
Fraction of cells in group (%)
20 40 60 80 100
log fold change
−5 0 5
MX1
IFI44L
IFI6
STAT1
DACT1
FAM13A
EDA
MYB
IGFBPL1
AC012417.1
CR559946.1
SLC9A9-AS2
SOX4
LRRN3
PECAM1
NREP
CCL5
NKG7
GZMH
CST7
GZMA
GZMK
CCL5
CST7
GZMK
CST7
CCL5
CD74
PHLDA3
BAX
RPS27L
PHPT1
S100A4
CCR6
KLRB1
ANXA2
CCL5
CXCR3
LYAR
GZMK
GZMK
GZMA
CEBPD
S100A4
FOXP3
IKZF2
FCER1G
RTKN2
FOXP3
S100A4
HLA-DRB1
ANXA2
TIGIT
FCRL3
IKZF2
FOXP3
PTGER2
FAM13A
ZNF462
CD79A
NEFL
CXCR5
PTGDR2
SEMA5A
LIMS1
TIGIT
KLRB1
CXCR5
ITGB1
AHNAK
ANXA2
CD82
D
Tnaive
Tnaive MX1
Tnaive Act
Tnaive SOX4
Tcm (Th0)
Tcm (Tfh)
Tcm (Th0) Act
Tcm (Th17)
Tcm (Th2)
Tcm PHLDA3
Tem (Th1)
Tem (Tph)
Tem (Th1) pre
Tem (Th1/17)
Temra (Th1)
Treg Naive
Treg Act
Treg Eff
1.0
0.8
0.6
0.4
0.2
0.0
E
5
0
CCR7
FAS
CD28
FOXP3
TBX21
GATA3
CCR4
RORC
CCR6
CXCR5
PDCD1
ID2
SOX4
MX1
DACT1
PHLDA3
FCRL3
F
0.0
0.5
1.0
The silhouette coefficient
G
Fraction of cells in group (%)
10 20 30 40 50 60
Mean expression in group
0.25 0.5 0.75
CCR2
CCR3
CX3CR1
CCRL2
CCR5
CXCR3
CCR9
CXCR5
Tph
Tfh
Marker genes
H
PD-1hiCXCR5-MHCII+ICOS+
PD-1hiCXCR5-MHCII-ICOS+
PD-1hiCXCR5-MHCII+ICOS-
PD-1hiCXCR5-MHCII-ICOS-
PD-1hiCXCR5+ICOS+
PD-1hiCXCR5+ICOS-
PD-1-CXCR5-
PD-1-CXCR5+
R
1
0
−1
SDY939

Figure S1. Global characterization of $CD4^+$ T cells, related to Figure 1

(A) UMAP plots depicting gene expressions of marker genes. (B) Sankey diagrams showing cluster assignment of cells in clusters L1 and L2. (C) Dot plot depicting signature genes' mean expression levels and percentage of cells expressing them across clusters. Marker genes for the plot were calculated by pairwise comparison with a group and the other groups iteratively using scanpy.tl.rank_genes_groups function. (D) Density plot of cell distributions for cluster L2 populations. (E) Heatmaps of marker genes' mean expression for each subtype in each sample. (F) Silhouette scores for each cluster for each sample based on Harmonized PCA. This demonstrates the distinctiveness of each cluster in every sample. (G) Dot plot depicting T peripheral helper (Tph) and T follicular helper (Tfh) marker genes' mean expression levels and percentage of cells expressing them in Tcm (Tfh) and Tem (Tph). (H) Pearson's correlation of transcriptome profiles between sorted T cell fractions, including Tph (SDY939) and our scRNA-seq (cluster L2).

Figure S2

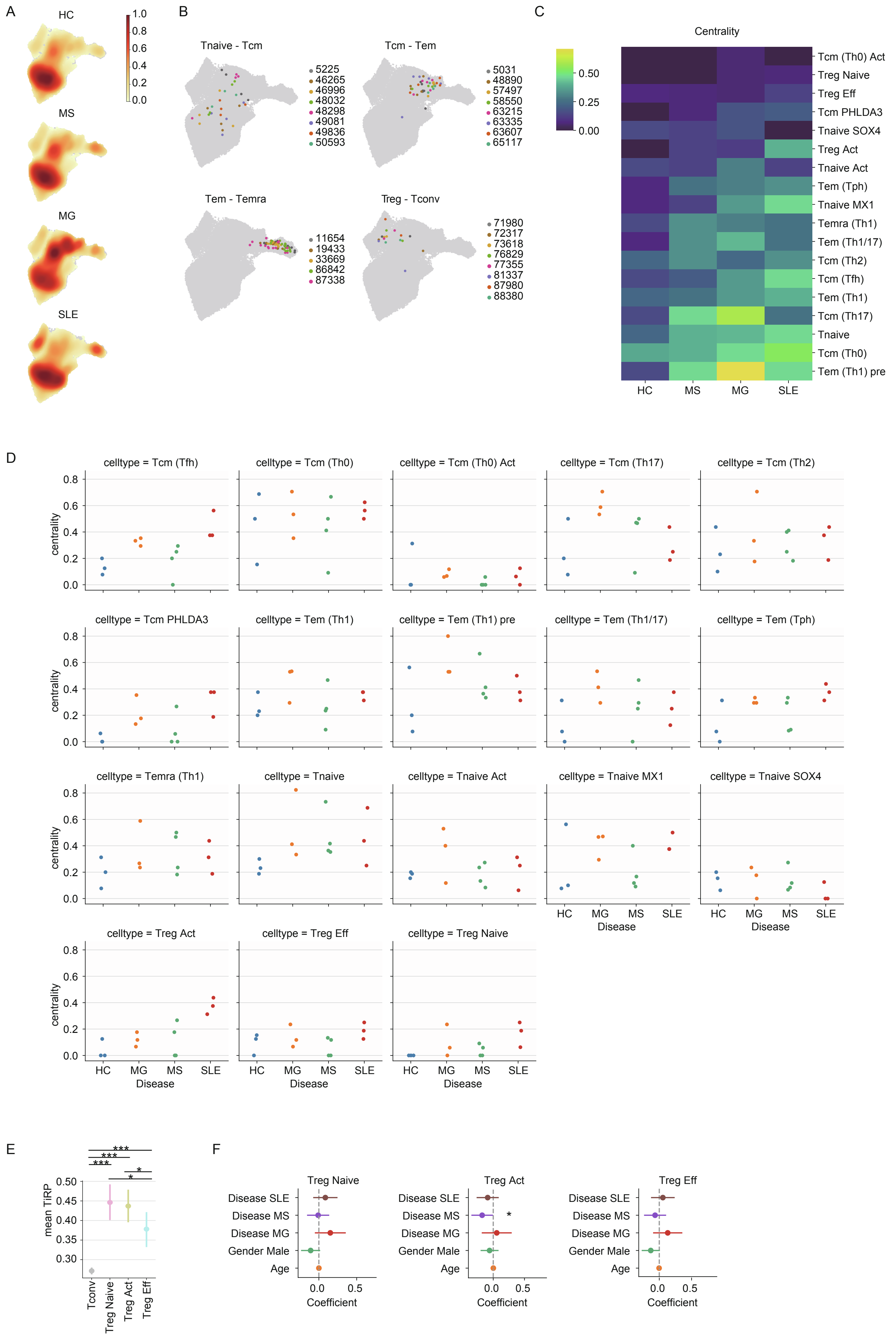
A
HC
MS
MG
SLE
1.0
0.8
0.6
0.4
0.2
0.0
B
Tnaive - Tcm
5225
46265
46996
48032
48298
49081
49836
50593
Tcm - Tem
5031
48890
57497
58550
63215
63335
63607
65117
Tem - Temra
11654
19433
33669
86842
87338
Treg - Tconv
71980
72317
73618
76829
77355
81337
87980
88380
C
Centrality
0.50
0.25
0.00
Tcm (Th0) Act
Treg Naive
Treg Eff
Tcm PHLDA3
Tnaive SOX4
Treg Act
Tnaive Act
Tem (Tph)
Tnaive MX1
Temra (Th1)
Tem (Th1/17)
Tcm (Th2)
Tcm (Tfh)
Tem (Th1)
Tcm (Th17)
Tnaive
Tcm (Th0)
Tem (Th1) pre
HC
MS
MG
SLE
D
celltype = Tcm (Tfh)
celltype = Tcm (Th0)
celltype = Tcm (Th0) Act
celltype = Tcm (Th17)
celltype = Tcm (Th2)
celltype = Tcm PHLDA3
celltype = Tem (Th1)
celltype = Tem (Th1) pre
celltype = Tem (Th1/17)
celltype = Tem (Tph)
celltype = Temra (Th1)
celltype = Tnaive
celltype = Tnaive Act
celltype = Tnaive MX1
celltype = Tnaive SOX4
celltype = Treg Act
celltype = Treg Eff
celltype = Treg Naive
centrality
0.8
0.6
0.4
0.2
0.0
HC
MG
MS
SLE
Disease
E
mean TiRP
0.50
0.45
0.40
0.35
0.30
***
***
***
*
*
Tconv
Treg Naive
Treg Act
Treg Eff
F
Treg Naive
Treg Act
Treg Eff
Disease SLE
Disease MS
Disease MG
Gender Male
Age
*
0.0
0.5
Coefficient

Figure S2. Centralities of TCR networks vary depending on the diseases, related to Figure 1

(A) Density plot of cell distributions for each disease. (B) Representative clones spanning across clusters. (C) Degree centrality of TCR networks for cluster L2. The average of each disease was shown. (D) Individual value of degree centrality of TCR networks. (E) Distribution of mean TiRP scores across Treg clusters. Pairwise Tukey-HSD posthoc tests. The multiple test correction was performed using a two-stage FDR strategy. *: $p_{adj} < 0.05$, **: $p_{adj} < 0.01$, ***: $p_{adj} < 0.001$. (F) Changes in TiRP scores in Treg clusters associated with disease states, age, and sex. The estimated coefficients and the 95 percentiles by multiple linear regression were plotted.

Figure S3

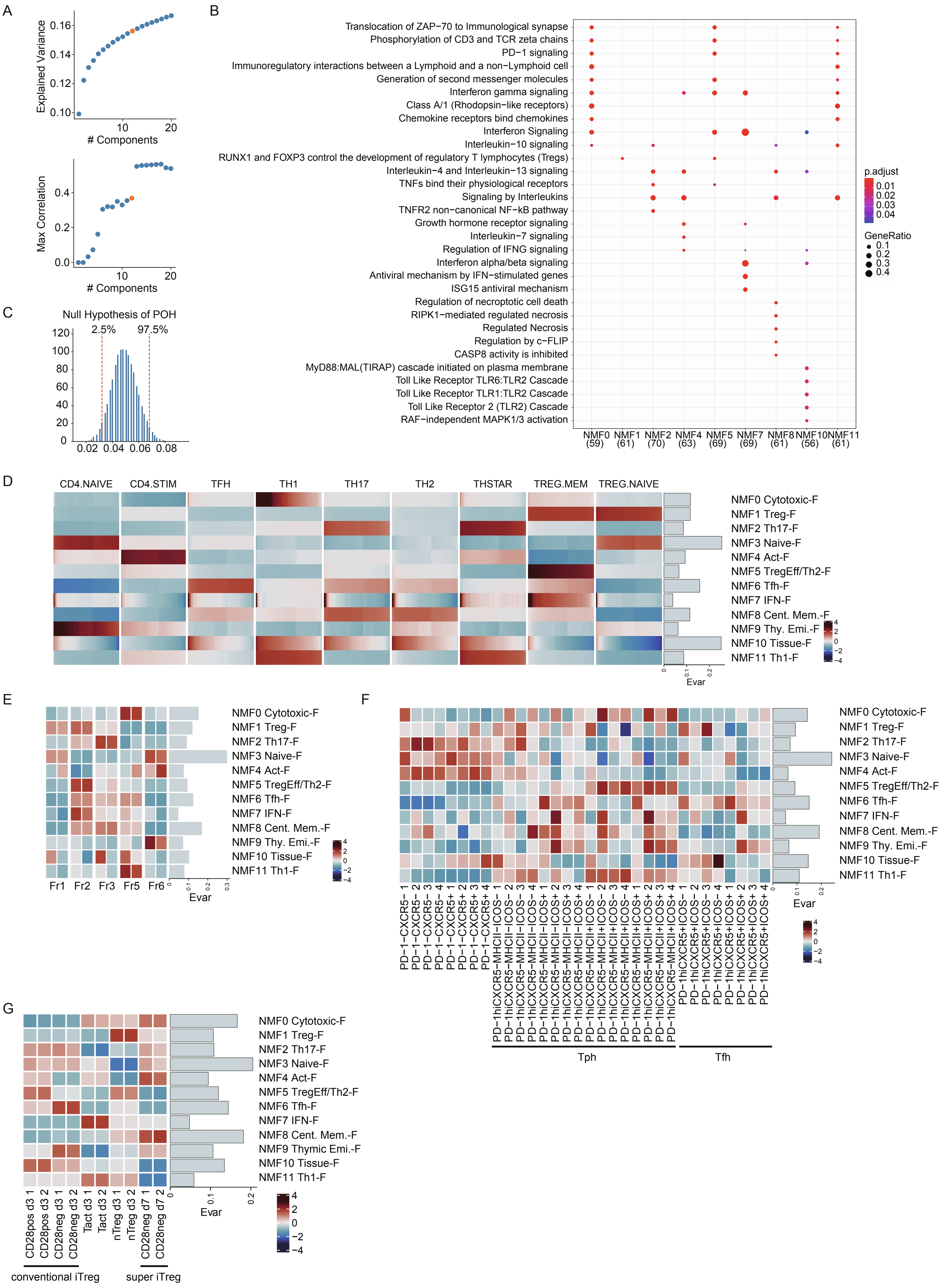
A
Explained Variance
0.16
0.14
0.12
0.10
10
20
Components
Max Correlation
0.4
0.2
0.0
Components
B
Translocation of ZAP−70 to Immunological synapse
Phosphorylation of CD3 and TCR zeta chains
PD−1 signaling
Immunoregulatory interactions between a Lymphoid and a non−Lymphoid cell
Generation of second messenger molecules
Interferon gamma signaling
Class A/1 (Rhodopsin−like receptors)
Chemokine receptors bind chemokines
Interferon Signaling
Interleukin−10 signaling
RUNX1 and FOXP3 control the development of regulatory T lymphocytes (Tregs)
Interleukin−4 and Interleukin−13 signaling
TNFs bind their physiological receptors
Signaling by Interleukins
TNFR2 non−canonical NF−kB pathway
Growth hormone receptor signaling
Interleukin−7 signaling
Regulation of IFNG signaling
Interferon alpha/beta signaling
Antiviral mechanism by IFN−stimulated genes
ISG15 antiviral mechanism
Regulation of necroptotic cell death
RIPK1−mediated regulated necrosis
Regulated Necrosis
Regulation by c−FLIP
CASP8 activity is inhibited
MyD88:MAL(TIRAP) cascade initiated on plasma membrane
Toll Like Receptor TLR6:TLR2 Cascade
Toll Like Receptor TLR1:TLR2 Cascade
Toll Like Receptor 2 (TLR2) Cascade
RAF−independent MAPK1/3 activation
NMF0 (59)
NMF1 (61)
NMF2 (70)
NMF4 (63)
NMF5 (69)
NMF7 (69)
NMF8 (61)
NMF10 (56)
NMF11 (61)
p.adjust
0.01
0.02
0.03
0.04
GeneRatio
0.1
0.2
0.3
0.4
C
Null Hypothesis of POH
2.5%
97.5%
120
100
80
60
40
20
0
0.02 0.04 0.06 0.08
D
CD4.NAIVE
CD4.STIM
TFH
TH1
TH17
TH2
THSTAR
TREG.MEM
TREG.NAIVE
NMF0 Cytotoxic-F
NMF1 Treg-F
NMF2 Th17-F
NMF3 Naive-F
NMF4 Act-F
NMF5 TregEff/Th2-F
NMF6 Tfh-F
NMF7 IFN-F
NMF8 Cent. Mem.-F
NMF9 Thy. Emi.-F
NMF10 Tissue-F
NMF11 Th1-F
Evar
E
Fr1
Fr2
Fr3
Fr5
Fr6
Evar
F
PD−1−CXCR5− 1
PD−1−CXCR5− 2
PD−1−CXCR5− 3
PD−1−CXCR5− 4
PD−1−CXCR5+ 1
PD−1−CXCR5+ 2
PD−1−CXCR5+ 3
PD−1−CXCR5+ 4
PD−1hiCXCR5−MHCII−ICOS− 1
PD−1hiCXCR5−MHCII−ICOS− 2
PD−1hiCXCR5−MHCII−ICOS− 3
PD−1hiCXCR5−MHCII−ICOS− 4
PD−1hiCXCR5−MHCII−ICOS+ 1
PD−1hiCXCR5−MHCII−ICOS+ 2
PD−1hiCXCR5−MHCII−ICOS+ 3
PD−1hiCXCR5−MHCII−ICOS+ 4
PD−1hiCXCR5−MHCII+ICOS− 1
PD−1hiCXCR5−MHCII+ICOS− 2
PD−1hiCXCR5−MHCII+ICOS− 3
PD−1hiCXCR5−MHCII+ICOS− 4
PD−1hiCXCR5−MHCII+ICOS+ 1
PD−1hiCXCR5−MHCII+ICOS+ 2
PD−1hiCXCR5−MHCII+ICOS+ 3
PD−1hiCXCR5−MHCII+ICOS+ 4
PD−1hiCXCR5+ICOS− 1
PD−1hiCXCR5+ICOS− 2
PD−1hiCXCR5+ICOS− 3
PD−1hiCXCR5+ICOS− 4
PD−1hiCXCR5+ICOS+ 1
PD−1hiCXCR5+ICOS+ 2
PD−1hiCXCR5+ICOS+ 3
PD−1hiCXCR5+ICOS+ 4
Tph
Tfh
Evar
G
NMF9 Thymic Emi.-F
CD28pos d3 1
CD28pos d3 2
CD28neg d3 1
CD28neg d3 2
Tact d3 1
Tact d3 2
nTreg d3 1
nTreg d3 2
CD28neg d7 1
CD28neg d7 2
conventional iTreg
super iTreg
Evar
4
2
0
−2
−4

Figure S3. NMF and NMF projection, related to Figure 2

(A) The statistics for the determination of the number of components. The Y-axis shows explained variance (upper) and maximum correlation of the inter-component (lower). The X-axis shows the number of components. Spearman's correlation between components of gene features was calculated. (B) Reactome pathways enriched in each gene feature. The dot size indicates the gene ratio or the fraction of genes found in the gene set, and the color indicates p_{adj}. (Table S5) (C) Histogram of the proportion of overlapped highly variable genes (POH) under the null hypothesis for this study setting. We randomly sampled 5000 POH in the null hypothesis calculated from the overlap between random 500 (number of HVGs for the calculation of POH) genes and 1271 (number of highly variable genes of $CD4^+$ T cell) genes. Red dashed lines show 2.5 and 97.5 percentiles. (D) Heatmap showing NMF values of DICE bulk RNA-seq datasets of sorted $CD4^+$ T cell fractions. Explained variance (Evar) was also shown on the right side. (E) Heatmap showing NMF values of sorted $CD4^+$ T cell fractions by Miyara's classification (JGAD000214). Explained variance (Evar) was also shown on the right side. (F) Heatmap showing NMF values of sorted Tph fractions (SDY939). Explained variance (Evar) was also shown on the right side. (G) Heatmap showing NMF values of induced Treg (iTreg) cells cultured in different conditions (DRA008294). Explained variance (Evar) was also shown on the right side.

Figure S4

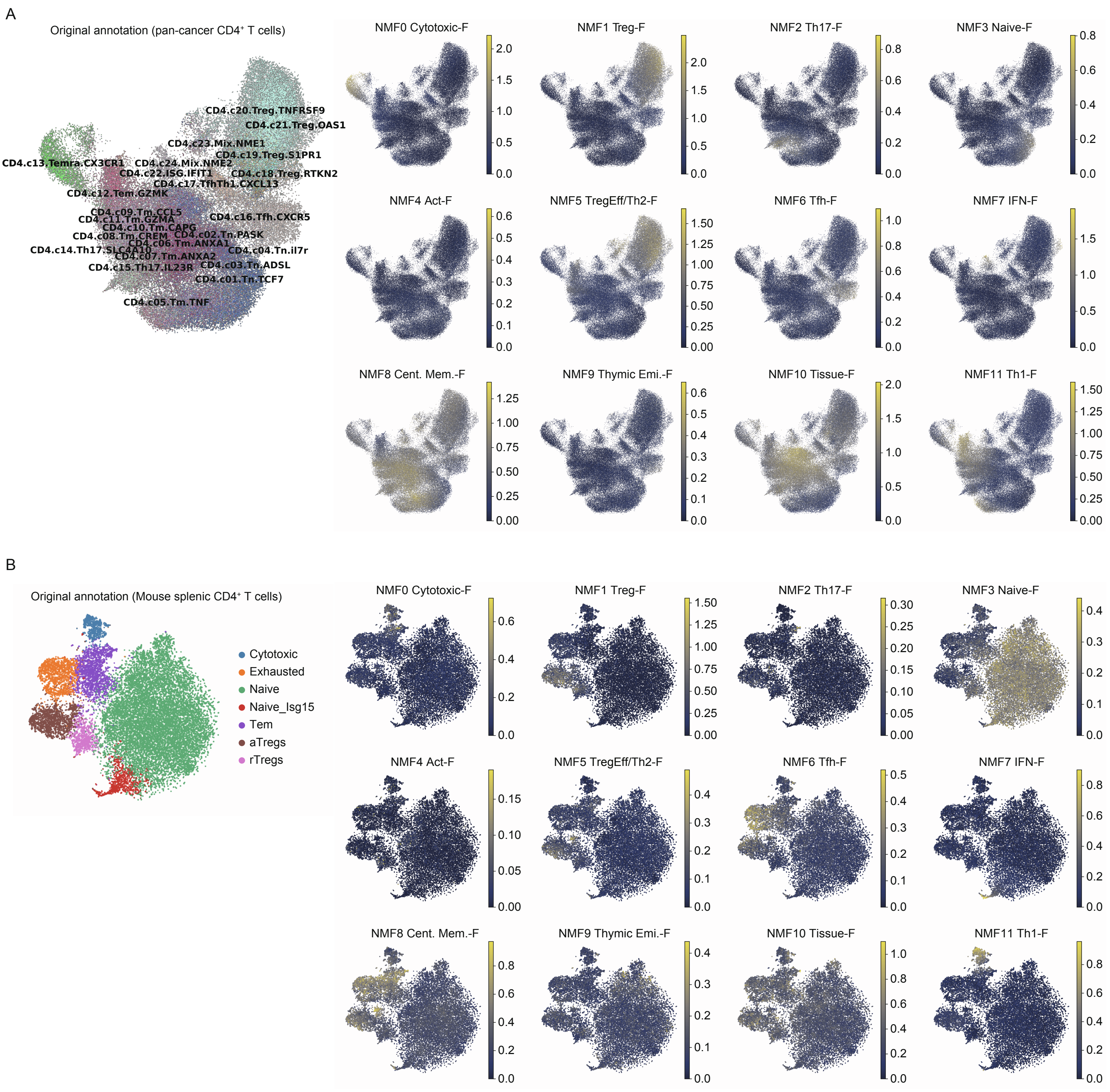
A
Original annotation (pan-cancer CD4⁺ T cells)
CD4.c20.Treg.TNFRSF9
CD4.c21.Treg.OAS1
CD4.c23.Mix.NME1
CD4.c19.Treg.S1PR1
CD4.c13.Temra.CX3CR1
CD4.c24.Mix.NME2
CD4.c22.ISG.IFIT1
CD4.c18.Treg.RTKN2
CD4.c17.TfhTh1.CXCL13
CD4.c12.Tem.GZMK
CD4.c09.Tm.CCL5
CD4.c11.Tm.GZMA
CD4.c16.Tfh.CXCR5
CD4.c10.Tm.CAPG
CD4.c08.Tm.CREM
CD4.c02.Tn.PASK
CD4.c06.Tm.ANXA1
CD4.c14.Th17.SLC4A10
CD4.c04.Tn.il7r
CD4.c07.Tm.ANXA2
CD4.c15.Th17.IL23R
CD4.c03.Tn.ADSL
CD4.c01.Tn.TCF7
CD4.c05.Tm.TNF
NMF0 Cytotoxic-F
NMF1 Treg-F
NMF2 Th17-F
NMF3 Naive-F
NMF4 Act-F
NMF5 TregEff/Th2-F
NMF6 Tfh-F
NMF7 IFN-F
NMF8 Cent. Mem.-F
NMF9 Thymic Emi.-F
NMF10 Tissue-F
NMF11 Th1-F
B
Original annotation (Mouse splenic CD4⁺ T cells)
Cytotoxic
Exhausted
Naive
Naive_Isg15
Tem
aTregs
rTregs
NMF0 Cytotoxic-F
NMF1 Treg-F
NMF2 Th17-F
NMF3 Naive-F
NMF4 Act-F
NMF5 TregEff/Th2-F
NMF6 Tfh-F
NMF7 IFN-F
NMF8 Cent. Mem.-F
NMF9 Thymic Emi.-F
NMF10 Tissue-F
NMF11 Th1-F

Figure S4. NMFproj applications in tumor-infiltrating T cells and mouse splenocytes, related to Figure 2

(A and B) UMAP plots showing original cell types (left) and projected NMF cell feature values (right) in pan-cancer tumor-infiltrating T cells scRNA-seq data (GSE156728) (A) and mouse splenic $CD4^+$ T cells (SCP490) (B).

Figure S5

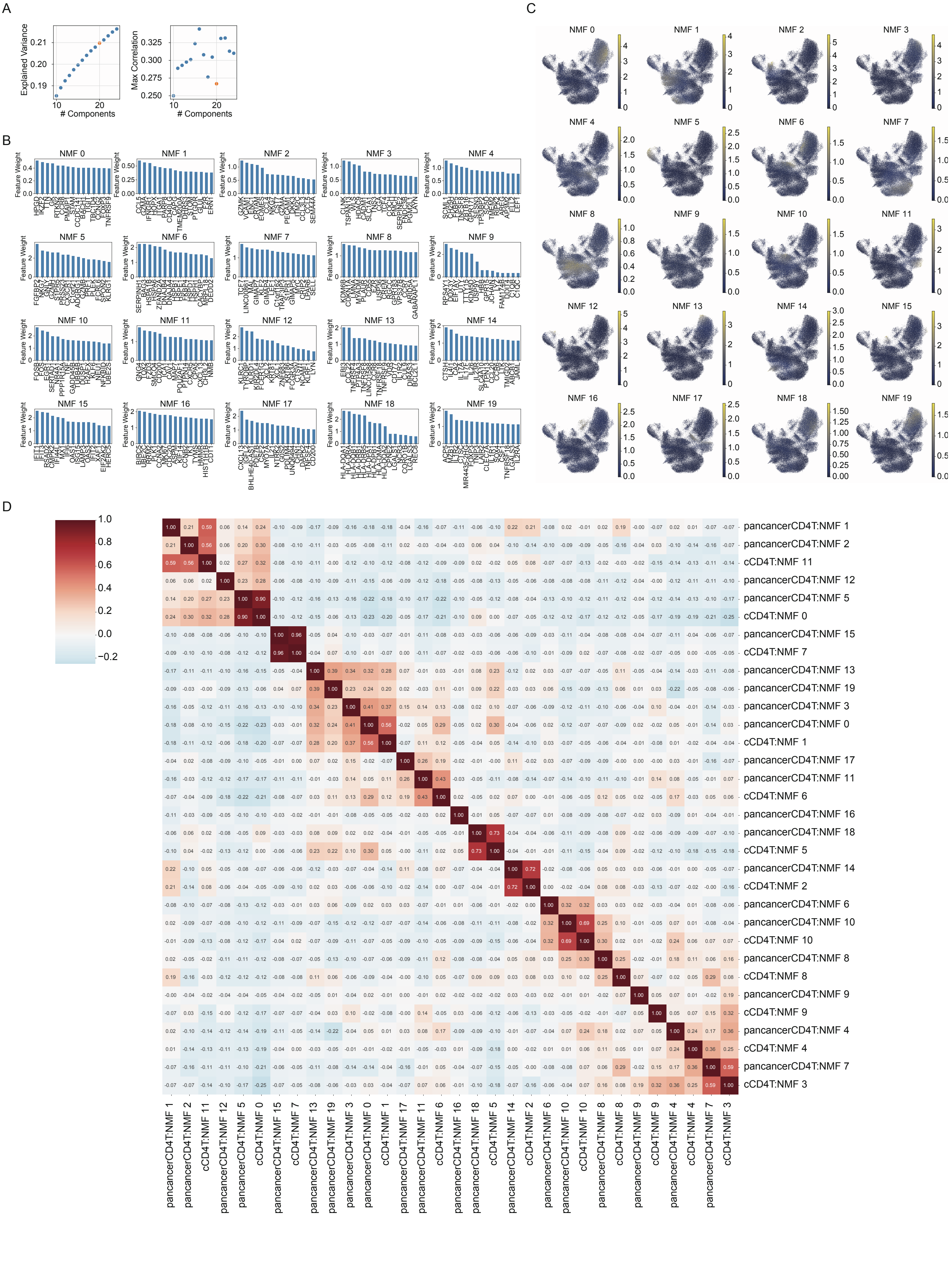

A
Explained Variance
Components
Max Correlation
Components
B
C
D
pancancerCD4T:NMF 1
pancancerCD4T:NMF 2
cCD4T:NMF 11
pancancerCD4T:NMF 12
pancancerCD4T:NMF 5
cCD4T:NMF 0
pancancerCD4T:NMF 15
cCD4T:NMF 7
pancancerCD4T:NMF 13
pancancerCD4T:NMF 19
pancancerCD4T:NMF 3
pancancerCD4T:NMF 0
cCD4T:NMF 1
pancancerCD4T:NMF 17
pancancerCD4T:NMF 11
cCD4T:NMF 6
pancancerCD4T:NMF 16
pancancerCD4T:NMF 18
cCD4T:NMF 5
pancancerCD4T:NMF 14
cCD4T:NMF 2
pancancerCD4T:NMF 6
pancancerCD4T:NMF 10
cCD4T:NMF 10
pancancerCD4T:NMF 8
cCD4T:NMF 8
pancancerCD4T:NMF 9
cCD4T:NMF 9
pancancerCD4T:NMF 4
cCD4T:NMF 4
pancancerCD4T:NMF 7
cCD4T:NMF 3

Figure S5. Comparison of NMF gene features defined in circulating $CD4^+$ T cells and pan-cancer $CD4^+$ T cells, related to Figure 2

(A) The statistics for the determination of the number of components for pan-cancer $CD4^+$ T cell dataset. The Y-axis shows explained variance (left) and maximum correlation of the inter-component (right). The X-axis shows the number of components. Spearman's correlation between components of gene features was calculated. 20 was selected for the number of components. (B) Bar plots showing gene feature weight for top 15 genes. (C) Cell feature value is shown on the UMAP plots. (D) Heatmap showing the correlation of gene features between the NMF gene feature value defined in peripheral blood $CD4^+$ T cells and the newly calculated gene feature value in a pan-cancer $CD4^+$ T dataset. For the two weight matrices, only common genes were selected, and the Pearson correlation coefficients were calculated.

Figure S6

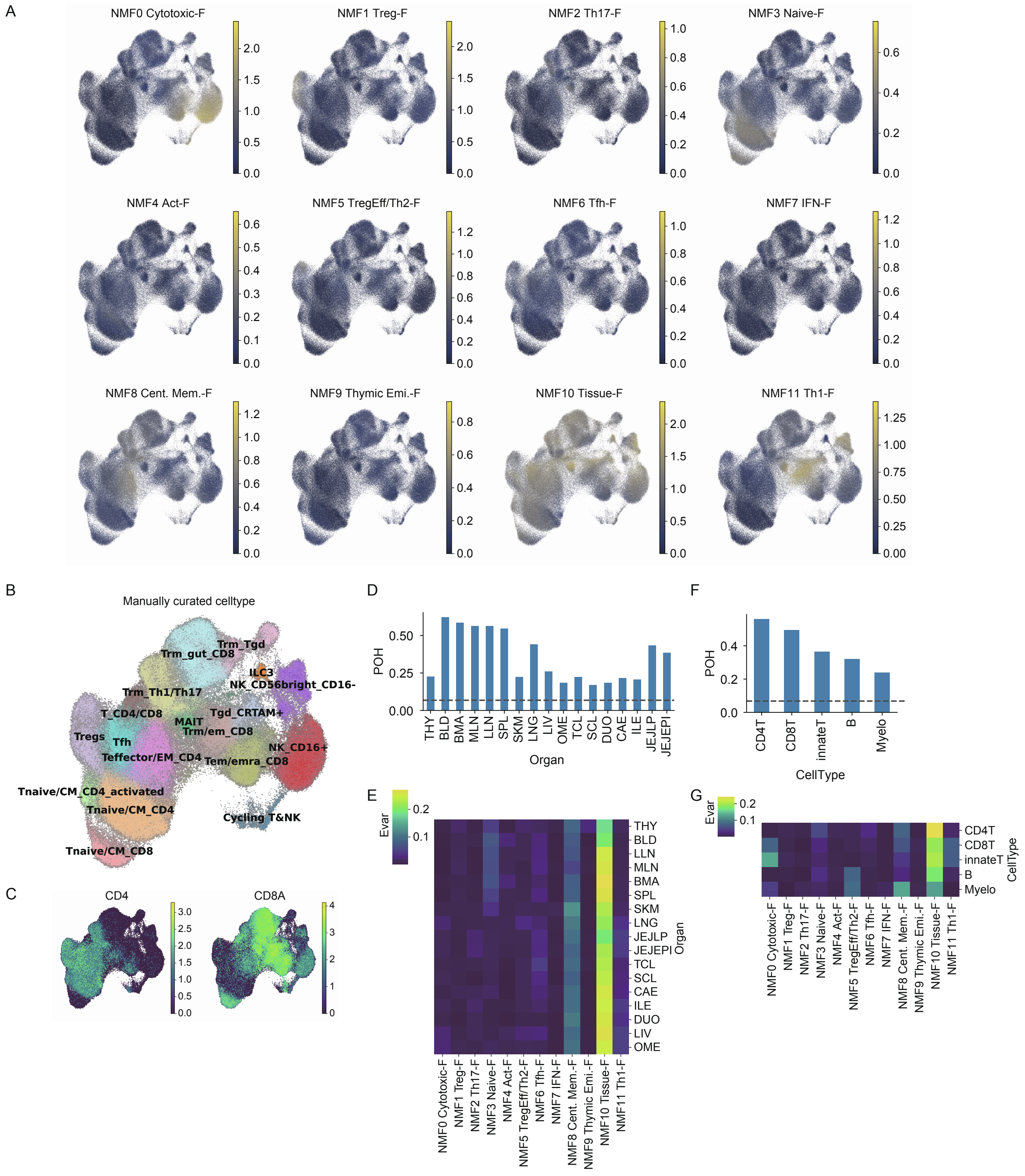
A
NMF0 Cytotoxic-F
NMF1 Treg-F
NMF2 Th17-F
NMF3 Naive-F
NMF4 Act-F
NMF5 TregEff/Th2-F
NMF6 Tfh-F
NMF7 IFN-F
NMF8 Cent. Mem.-F
NMF9 Thymic Emi.-F
NMF10 Tissue-F
NMF11 Th1-F
B
Manually curated celltype
Trm_Tgd
Trm_gut_CD8
ILC3
NK_CD56bright_CD16-
Trm_Th1/Th17
T_CD4/CD8
Tgd_CRTAM+
MAIT
Trm/em_CD8
Tregs
Tfh
NK_CD16+
Teffector/EM_CD4
Tem/emra_CD8
Tnaive/CM_CD4_activated
Tnaive/CM_CD4
Cycling T&NK
Tnaive/CM_CD8
C
CD4
CD8A
D
POH
THY BLD BMA MLN LLN SPL SKM LNG LIV OME TCL SCL DUO CAE ILE JEJLP JEJEPI
Organ
E
Evar
THY BLD LLN MLN BMA SPL SKM LNG JEJLP JEJEPI TCL SCL CAE ILE DUO LIV OME
Organ
F
POH
CD4T CD8T innateT B Myelo
CellType
G
Evar
CD4T CD8T innateT B Myelo
CellType

Figure S6. NMFproj contributes to interpreting cross-tissue T cells, related to Figure 2

(A) Projected NMF cell feature value of cross-tissue T cells scRNA-seq datasets on the UMAP plots. The T & innate lymphoid cells dataset was used for the analysis (https://www.tissueimmunecellatlas.org/). (B and C) Original cell types (B) and the expression of CD4 and CD8A (C) were shown on the UMAP plots. (D and E) Distribution of POH (D) and Evar (E) in each tissue. THY: Thymus, BLD: Blood, BMA: Bone marrow, MLN: Mesenchymal lymph nodes, LLN: Lung-draining lymph nodes, SPL: Spleen, SKM: Skeletal muscle, LNG: Lung, LIV: Liver, OME: Omentum, TCL: Transverse colon, SCL: Sigmoid colon, DUO: Duodenum, CAE: Caecum, ILE: Ileum, JEJLP: Jejunum lamina propria, JEJEPI: Jejunum epithelial. The dashed line indicates the 97.5 percentile of simulated null distribution (Fig. S3C). (F and G) Distribution of POH (F) and Evar (G) in each cell type. B and Myeloid cells were also added to the analysis. The dashed line indicates the 97.5 percentile of simulated null distribution (Fig. S3C).

Figure S7

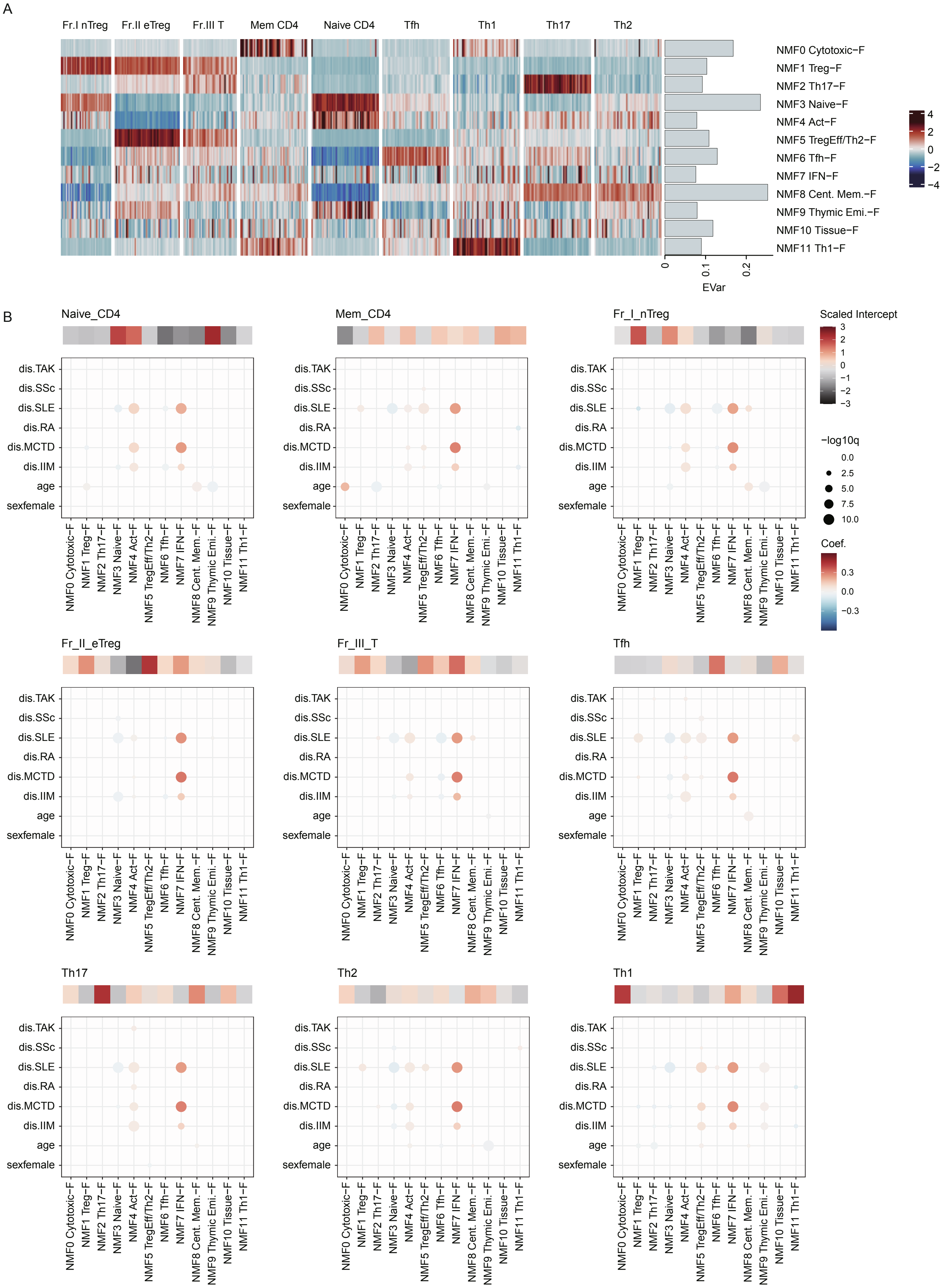
A
Fr.I nTreg
Fr.II eTreg
Fr.III T
Mem CD4
Naive CD4
Tfh
Th1
Th17
Th2
NMF0 Cytotoxic−F
NMF1 Treg−F
NMF2 Th17−F
NMF3 Naive−F
NMF4 Act−F
NMF5 TregEff/Th2−F
NMF6 Tfh−F
NMF7 IFN−F
NMF8 Cent. Mem.−F
NMF9 Thymic Emi.−F
NMF10 Tissue−F
NMF11 Th1−F
EVar
B
Naive_CD4
Mem_CD4
Fr_I_nTreg
Fr_II_eTreg
Fr_III_T
Tfh
Th17
Th2
Th1
dis.TAK
dis.SSc
dis.SLE
dis.RA
dis.MCTD
dis.IIM
age
sexfemale
Scaled Intercept
−log10q
Coef.

Figure S7. NMFproj reveals disease-specific qualitative changes, related to Figure 2

(A) Heatmap showing NMF values of sorted $CD4^+$ T cell fractions collected from autoimmune patients (E-GEAD-397). Explained variance (Evar) was also shown on the right side. (B) Dot plot depicting NMF cell feature changes in each cell type in E-GEAD-397. Dot colors show coefficients, and sizes show the significance of GLM. GLM was performed with a model, cell frequency, or NMF cell feature ~ disease + age + gender. The heatmaps at the top of each plot display the standardized values of the GLM intercept for each feature, representing the baseline activity of each feature in each cell. IIM: idiopathic inflammatory myopathy, MCTD: mixed connective tissue disease, RA: rheumatoid arthritis, SLE: systemic lupus erythematosus, SSc: systemic sclerosis, TAK: Takayasu arteritis.

Figure S8

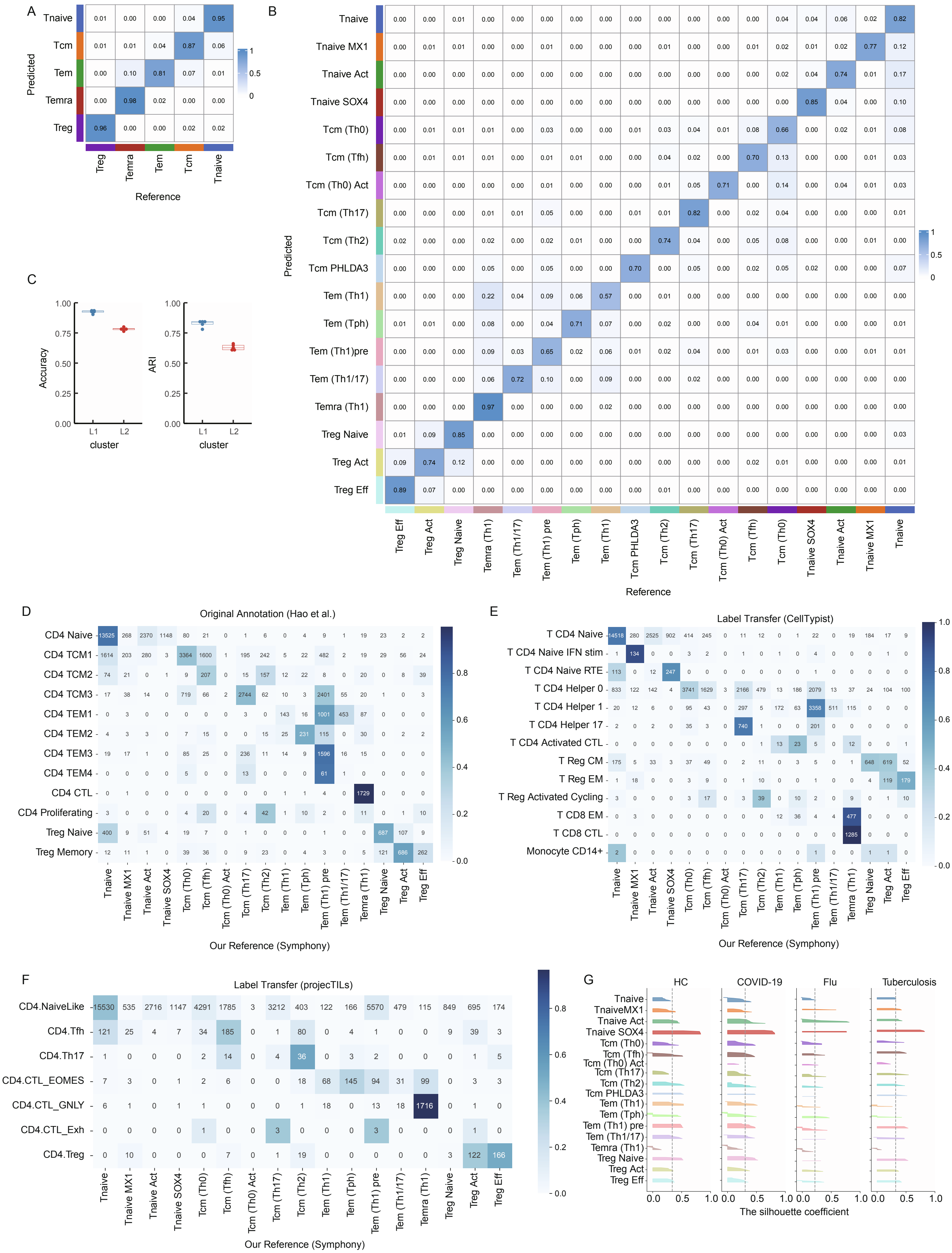

A
B
C
D
E
F
G
Predicted
Reference
Accuracy
ARI
cluster
Original Annotation (Hao et al.)
Label Transfer (CellTypist)
Label Transfer (projecTILs)
Our Reference (Symphony)
HC
COVID-19
Flu
Tuberculosis
The silhouette coefficient

Figure S8. Quality check of reference mapping using Symphony, related to Figure 3

(A-C) Heatmap depicting the precision of reference mapping at L1 (A) and L2 (B) levels, along with plots showing accuracy and Adjusted Rand Index (ARI) (C). We divided our dataset (n=13) into five non-overlapping groups and performed five-fold cross-validation (CV). In this CV, we calculated corrected PCs with Harmony using four groups, constructed a Symphony reference, and mapped the remaining independent sample. These procedures were repeated five times for each fold. We compared the predicted labels with the labels we had pre-annotated to evaluate accuracy. As evaluation metrics, we present the accuracy and ARI at cluster L1 and L2 levels per CV. The lower and upper hinges represent the first quartile and the third quartile. Whiskers show the smallest or largest values that are within 1.5 times the IQR from the hinges. (D) Transferred labels using our pipeline were compared to the original annotations for human PBMC cellular indexing of transcriptomes and epitopes by sequencing (CITE-seq) data from Hao *et al.* The numbers represent the absolute cell count for each paired annotation, while colors denote the fraction relative to the total count in the original study's annotation. (E and F) Transferred labels using our pipeline were compared to labels assigned by CellTypist with the model, COVID19_HumanChallenge_Blood (E) and ProjecTILs with human $CD4^{+}$ TILs (F) for human PBMC CITE-seq data from Hao et al. The numbers represent the absolute cell count for each paired annotation, while colors denote the fraction relative to the total count in the original study's annotation. (G) Silhouette scores for each sample based on the corrected PCA by Symphony and the cluster L2 assigned by Symphony. Each panel represents a different condition. The number of cells per disease is as follows: Healthy Controls (HC) 7,671 cells, COVID-19 3,475 cells, Flu 421 cells, and Tuberculosis 8,767 cells.

Figure S9

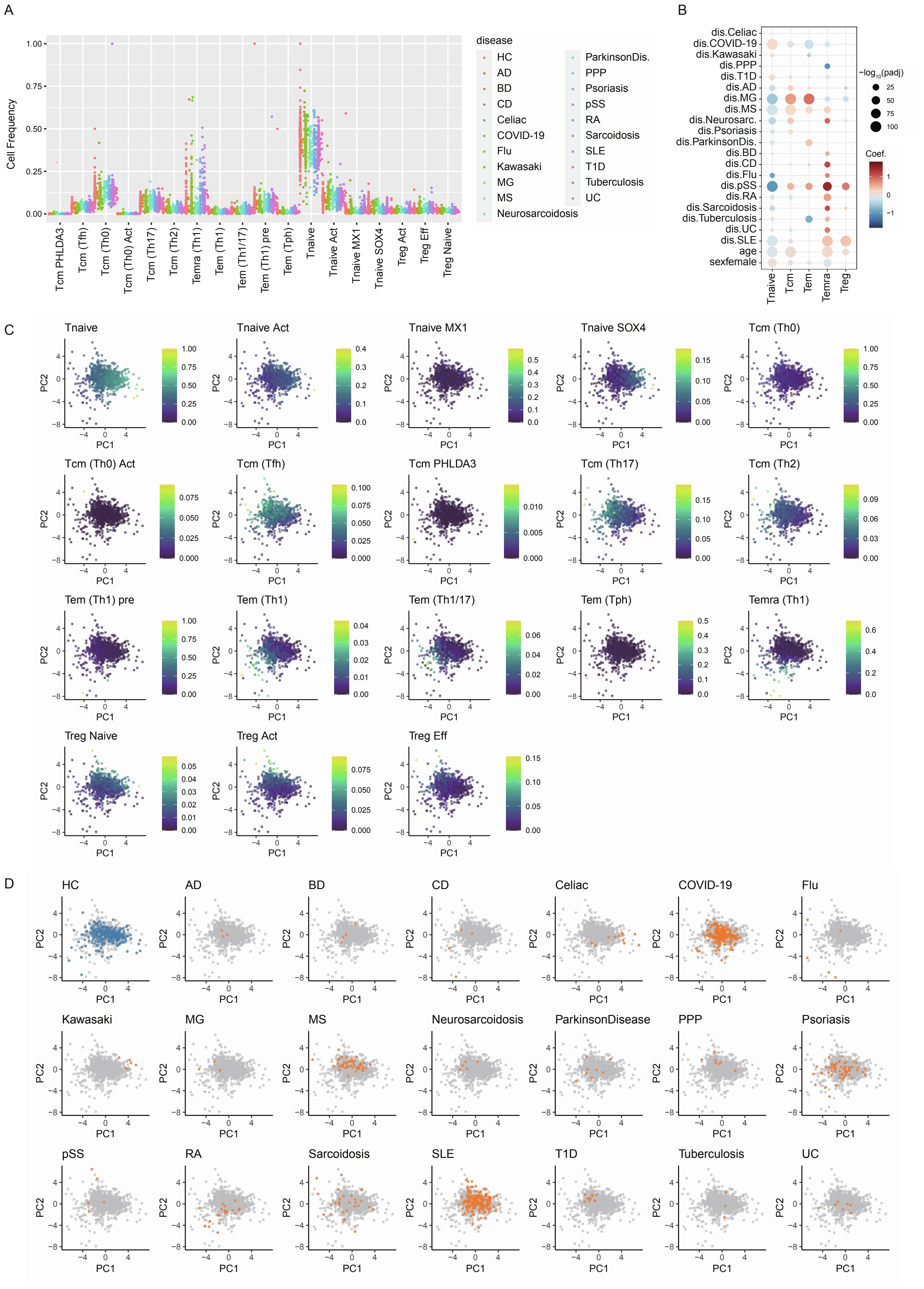
A
B
C
D
Cell Frequency
disease
HC
AD
BD
CD
Celiac
COVID-19
Flu
Kawasaki
MG
MS
Neurosarcoidosis
ParkinsonDis.
PPP
Psoriasis
pSS
RA
Sarcoidosis
SLE
T1D
Tuberculosis
UC
Tcm PHLDA3
Tcm (Tfh)
Tcm (Th0)
Tcm (Th0) Act
Tcm (Th17)
Tcm (Th2)
Temra (Th1)
Tem (Th1)
Tem (Th1/17)
Tem (Th1) pre
Tem (Tph)
Tnaive
Tnaive Act
Tnaive MX1
Tnaive SOX4
Treg Act
Treg Eff
Treg Naive
dis.Celiac
dis.COVID-19
dis.Kawasaki
dis.PPP
dis.T1D
dis.AD
dis.MG
dis.MS
dis.Neurosarc.
dis.Psoriasis
dis.ParkinsonDis.
dis.BD
dis.CD
dis.Flu
dis.pSS
dis.RA
dis.Sarcoidosis
dis.Tuberculosis
dis.UC
dis.SLE
age
sexfemale
Tnaive
Tcm
Tem
Temra
Treg
−log10(padj)
Coef.
PC1
PC2
Tcm PHLDA3
Treg Naive
Treg Act
Treg Eff
ParkinsonDisease

Figure S9. Quantitative alterations revealed by meta-analysis , related to Figure 3

(A) Swarm plot showing frequencies of cell types in each sample. (B) Dot plot showing changes in cell frequency at cluster L1 resolution. Dot colors show coefficients, and sizes show the significance of the Generalized Linear Model (Methods). Detailed statistics can be found in Table S7. Only significant dots ($p_{adj} < 0.05$) are shown. (C) Cell frequencies of each population are shown on the PCA plots. PCA was performed using cell frequencies of each cluster in each individual. (D) Distribution of samples for each disease on the PCA plot.

Figure S10

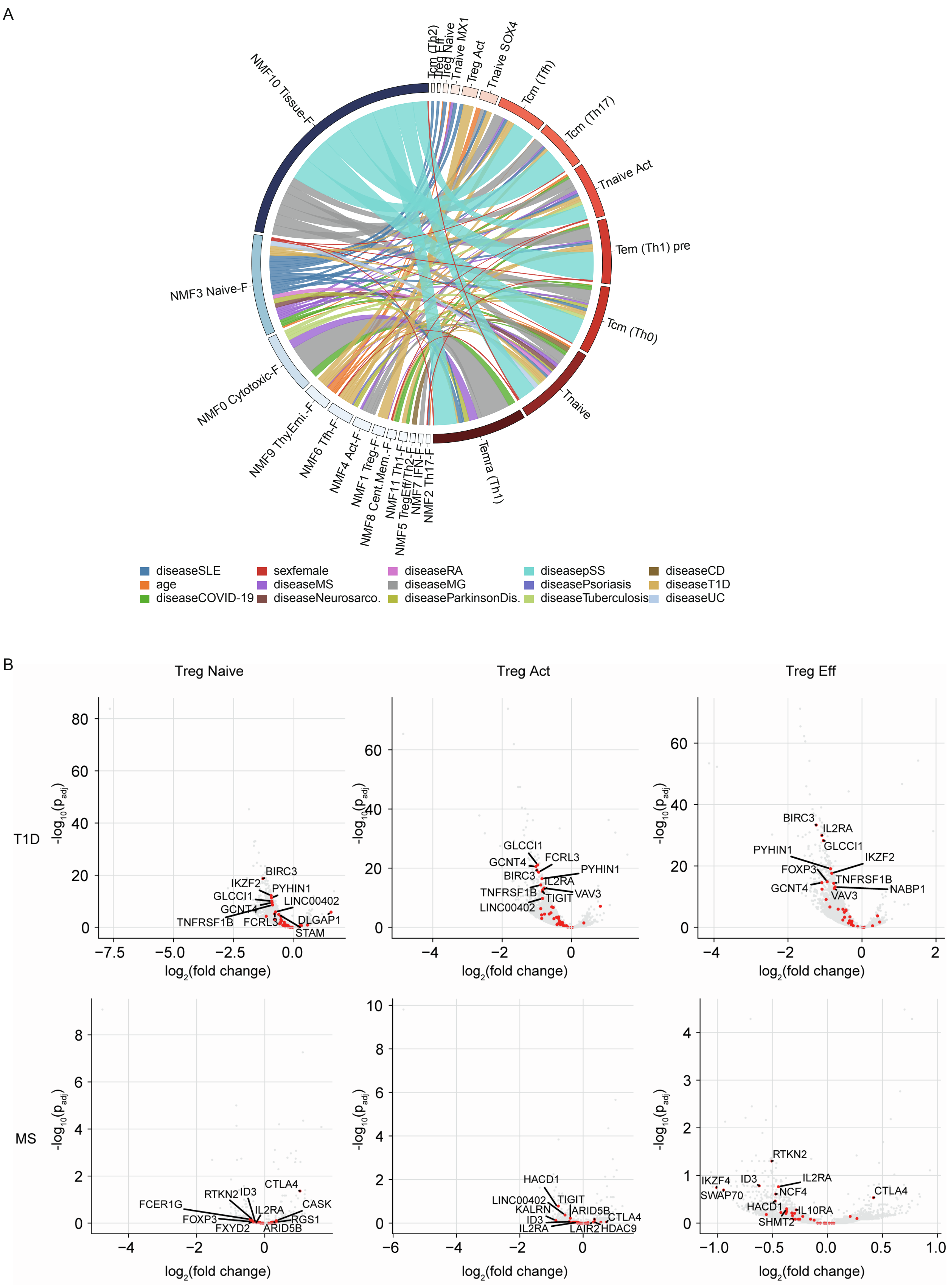
A
NMF10 Tissue-F
NMF3 Naive-F
NMF0 Cytotoxic-F
NMF9 ThyEmi.-F
NMF6 Tfh-F
NMF4 Act-F
NMF1 Treg-F
NMF8 Cent.Mem.-F
NMF11 Th1-F
NMF5 TregEff/Th2-F
NMF7 IFN-F
NMF2 Th17-F
Tcm (Th2)
Treg Eff
Treg Naive
Tnaive MX1
Treg Act
Tnaive SOX4
Tcm (Tfh)
Tcm (Th17)
Tnaive Act
Tem (Th1) pre
Tcm (Th0)
Tnaive
Temra (Th1)
diseaseSLE
age
diseaseCOVID-19
sexfemale
diseaseMS
diseaseNeurosarco.
diseaseRA
diseaseMG
diseaseParkinsonDis.
diseasepSS
diseasePsoriasis
diseaseTuberculosis
diseaseCD
diseaseT1D
diseaseUC
B
Treg Naive
Treg Act
Treg Eff
T1D
MS
-log10(padj)
log2(fold change)
BIRC3
IKZF2
PYHIN1
GLCCI1
LINC00402
GCNT4
TNFRSF1B
FCRL3
DLGAP1
STAM
FCRL3
PYHIN1
IL2RA
TIGIT
VAV3
IKZF2
FOXP3
NABP1
CTLA4
FCER1G
RTKN2
ID3
CASK
FOXP3
FXYD2
ARID5B
RGS1
HACD1
LINC00402
KALRN
ARID5B
CTLA4
LAIR2
HDAC9
RTKN2
IKZF4
SWAP70
NCF4
HACD1
IL10RA
SHMT2

Figure S10. Alterations in $CD4^+$ T cells revealed by meta-analysis, related to Figure 3

(A) Chord diagram showing the top 100 significant associations with negative coefficients between NMF features and cells in each condition, calculated by GLM (Methods). Detailed statistics are shown in Table S9. The thickness of edges indicates the absolute value of the coefficient of GLM, and colors indicate conditions such as diseases, gender, and age.
(B) Volcano plots showing differentially expressed genes in Treg clusters in T1D and MS. Variation in gene expression between diseases and healthy controls in three Treg clusters was calculated using sc.tl.rank_genes_groups (method='t-test_overestim_var') for each disease. To avoid the influence of batch effects between projects, only the GSE144744 dataset was used for MS. Genes with a mean normalized expression value of 0.1 or higher are shown in the volcano plot. Genes that are in the top 50 for NMF1 feature values are marked in red.

Figure S11

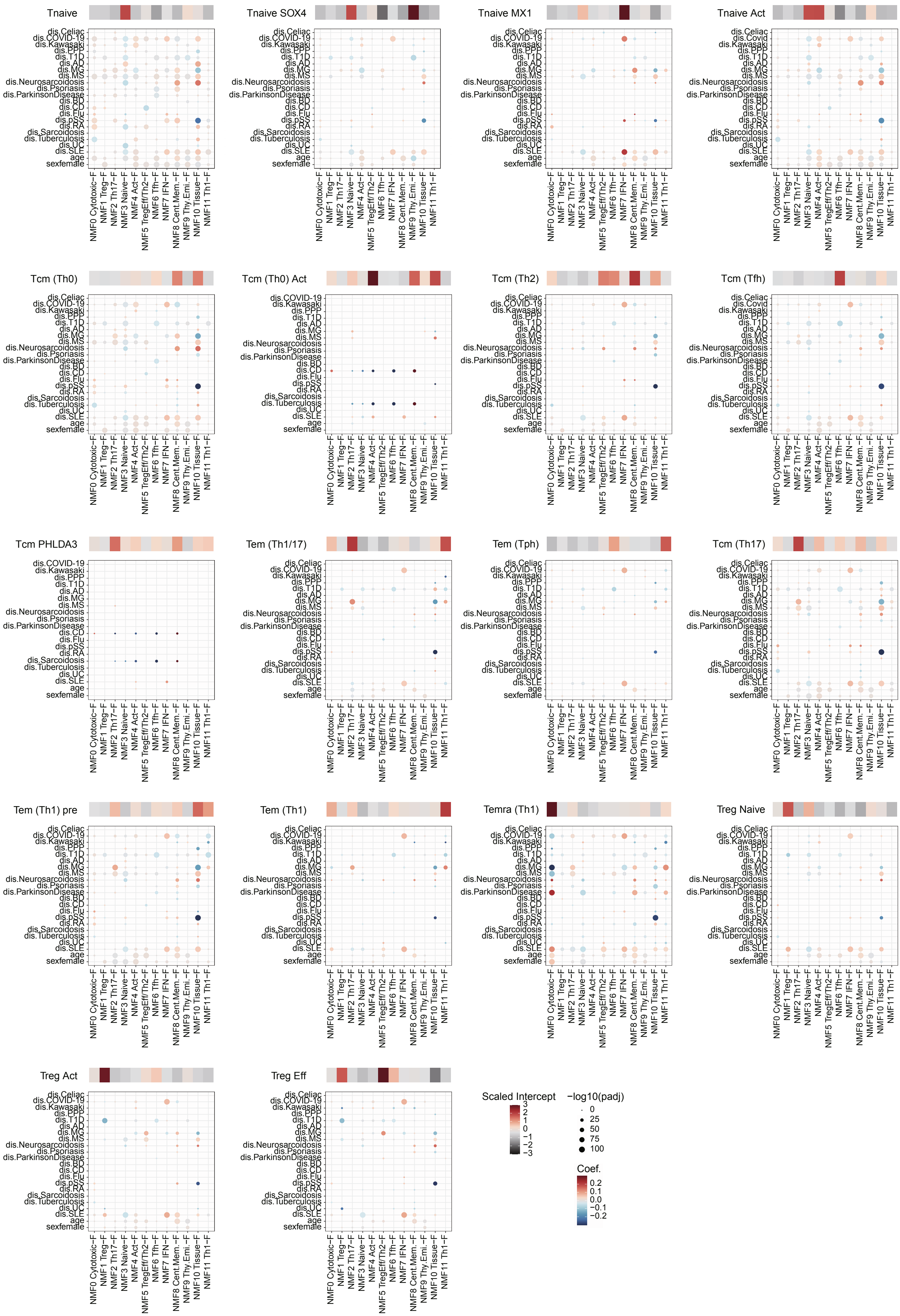
Tnaive
Tnaive SOX4
Tnaive MX1
Tnaive Act
Tcm (Th0)
Tcm (Th0) Act
Tcm (Th2)
Tcm (Tfh)
Tcm PHLDA3
Tem (Th1/17)
Tem (Tph)
Tcm (Th17)
Tem (Th1) pre
Tem (Th1)
Temra (Th1)
Treg Naive
Treg Act
Treg Eff
dis.Celiac
dis.COVID-19
dis.Covid
dis.Kawasaki
dis.PPP
dis.T1D
dis.AD
dis.MG
dis.MS
dis.Neurosarcoidosis
dis.Psoriasis
dis.ParkinsonDisease
dis.BD
dis.CD
dis.Flu
dis.pSS
dis.RA
dis.Sarcoidosis
dis.Tuberculosis
dis.UC
dis.SLE
age
sexfemale
NMF0 Cytotoxic−F
NMF1 Treg−F
NMF2 Th17−F
NMF3 Naive−F
NMF4 Act−F
NMF5 TregEff/Th2−F
NMF6 Tfh−F
NMF7 IFN−F
NMF8 Cent.Mem.−F
NMF9 Thy.Emi.−F
NMF10 Tissue−F
NMF11 Th1−F
Scaled Intercept
3
2
1
0
−1
−2
−3
−log10(padj)
0
25
50
75
100
Coef.
0.2
0.1
0.0
−0.1
−0.2

Figure S11. NMF cell feature changes depending on diseases, related to Figure 3

Dot plots depicting NMF cell feature changes in each cell type. Dot colors show coefficients, and sizes show the significance of GLM. GLM was performed with a model, NMF cell feature ~ disease + age + gender + project. Only significant dots ($p_{adj} < 0.05$) are shown. The heatmaps at the top of each plot display the standardized values of the GLM intercept for each feature, representing the baseline activity of each feature in each cell.

Figure S12

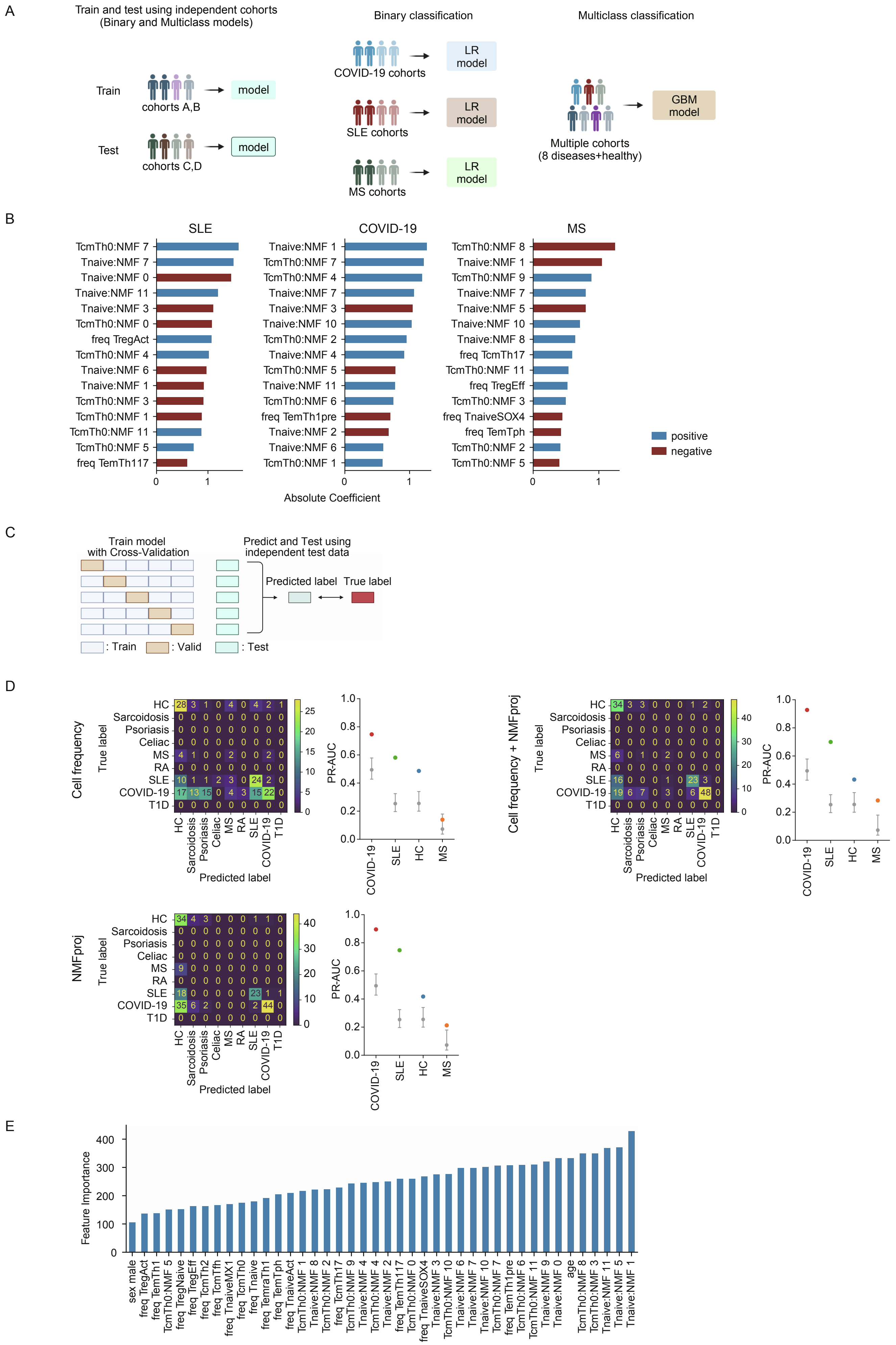
A
Train and test using independent cohorts
(Binary and Multiclass models)
Train
cohorts A,B
model
Test
cohorts C,D
model
Binary classification
COVID-19 cohorts
LR model
SLE cohorts
LR model
MS cohorts
LR model
Multiclass classification
Multiple cohorts
(8 diseases+healthy)
GBM model
B
SLE
TcmTh0:NMF 7
Tnaive:NMF 7
Tnaive:NMF 0
Tnaive:NMF 11
Tnaive:NMF 3
TcmTh0:NMF 0
freq TregAct
TcmTh0:NMF 4
Tnaive:NMF 6
Tnaive:NMF 1
TcmTh0:NMF 3
TcmTh0:NMF 1
TcmTh0:NMF 11
TcmTh0:NMF 5
freq TemTh117
COVID-19
Tnaive:NMF 1
TcmTh0:NMF 7
TcmTh0:NMF 4
Tnaive:NMF 7
Tnaive:NMF 3
Tnaive:NMF 10
TcmTh0:NMF 2
Tnaive:NMF 4
TcmTh0:NMF 5
Tnaive:NMF 11
TcmTh0:NMF 6
freq TemTh1pre
Tnaive:NMF 2
Tnaive:NMF 6
TcmTh0:NMF 1
MS
TcmTh0:NMF 8
Tnaive:NMF 1
TcmTh0:NMF 9
Tnaive:NMF 7
Tnaive:NMF 5
Tnaive:NMF 10
Tnaive:NMF 8
freq TcmTh17
TcmTh0:NMF 11
freq TregEff
TcmTh0:NMF 3
freq TnaiveSOX4
freq TemTph
TcmTh0:NMF 2
TcmTh0:NMF 5
Absolute Coefficient
positive
negative
C
Train model
with Cross-Validation
Predict and Test using
independent test data
Predicted label
True label
: Train
: Valid
: Test
D
Cell frequency
Cell frequency + NMFproj
NMFproj
True label
Predicted label
HC
Sarcoidosis
Psoriasis
Celiac
MS
RA
SLE
COVID-19
T1D
PR-AUC
COVID-19
SLE
HC
MS
E
Feature Importance
sex male
freq TregAct
freq TemTh1
TcmTh0:NMF 5
freq TregNaive
freq TregEff
freq TcmTh2
freq TcmTfh
freq TnaiveMX1
freq TcmTh0
freq Tnaive
freq TemraTh1
freq TemTph
freq TnaiveAct
TcmTh0:NMF 1
Tnaive:NMF 8
TcmTh0:NMF 2
freq TcmTh17
TcmTh0:NMF 9
Tnaive:NMF 4
TcmTh0:NMF 4
Tnaive:NMF 2
freq TemTh117
TcmTh0:NMF 0
freq TnaiveSOX4
Tnaive:NMF 3
TcmTh0:NMF 10
Tnaive:NMF 6
Tnaive:NMF 7
Tnaive:NMF 10
TcmTh0:NMF 7
freq TemTh1pre
TcmTh0:NMF 6
TcmTh0:NMF 11
Tnaive:NMF 9
Tnaive:NMF 0
age
TcmTh0:NMF 8
TcmTh0:NMF 3
Tnaive:NMF 11
Tnaive:NMF 5
Tnaive:NMF 1

Figure S12. Predicting Autoimmune Diseases Based on $CD4^+$ T Cell Profiles Using Machine Learning, related to Figure 3

(A) Overview of autoimmune disease prediction using machine learning. LR: Logistic Regression, GBM: Gradient Boosting Machine. (B) Bar plots showing absolute coefficient for logistic regression models. The top 15 variables are shown. Colors indicate whether the coefficient is positive or negative. (C) Strategy for multiclass classification by machine learning. The training was performed with cross-validation. The evaluation was performed using the independent dataset of training datasets. (D) Evaluations of models trained by cell frequencies (upper left panel), by NMFproj values in Tnaive and Tcm (Th0) (lower left panel), and by both cell frequencies and NMFproj values (right panel). The confusion matrix (left) and PR-AUC (right) are shown. The dashed lines in the PR-AUC plot show the expected PR-AUC scores in random models. The number of samples used for the training is 263, 27, 62, 11, 43, 20, 156, 116, 11 subjects for HC, sarcoidosis, psoriasis, celiac disease, MS, RA, SLE, COVID-19, and T1D, and evaluated on 89, 43, 43, and 9 subjects from independent data sets. Error bars indicate a 95% confidence interval for the random model calculated by 500 times permutations of true labels. The accuracy for each model is cell frequency model: 0.413, NMF model: 0.484, and cell frequency + NMF model: 0.527, respectively. (E) Bar plot showing feature importance of variables in the cell frequency + NMF GBM model.

Figure S13

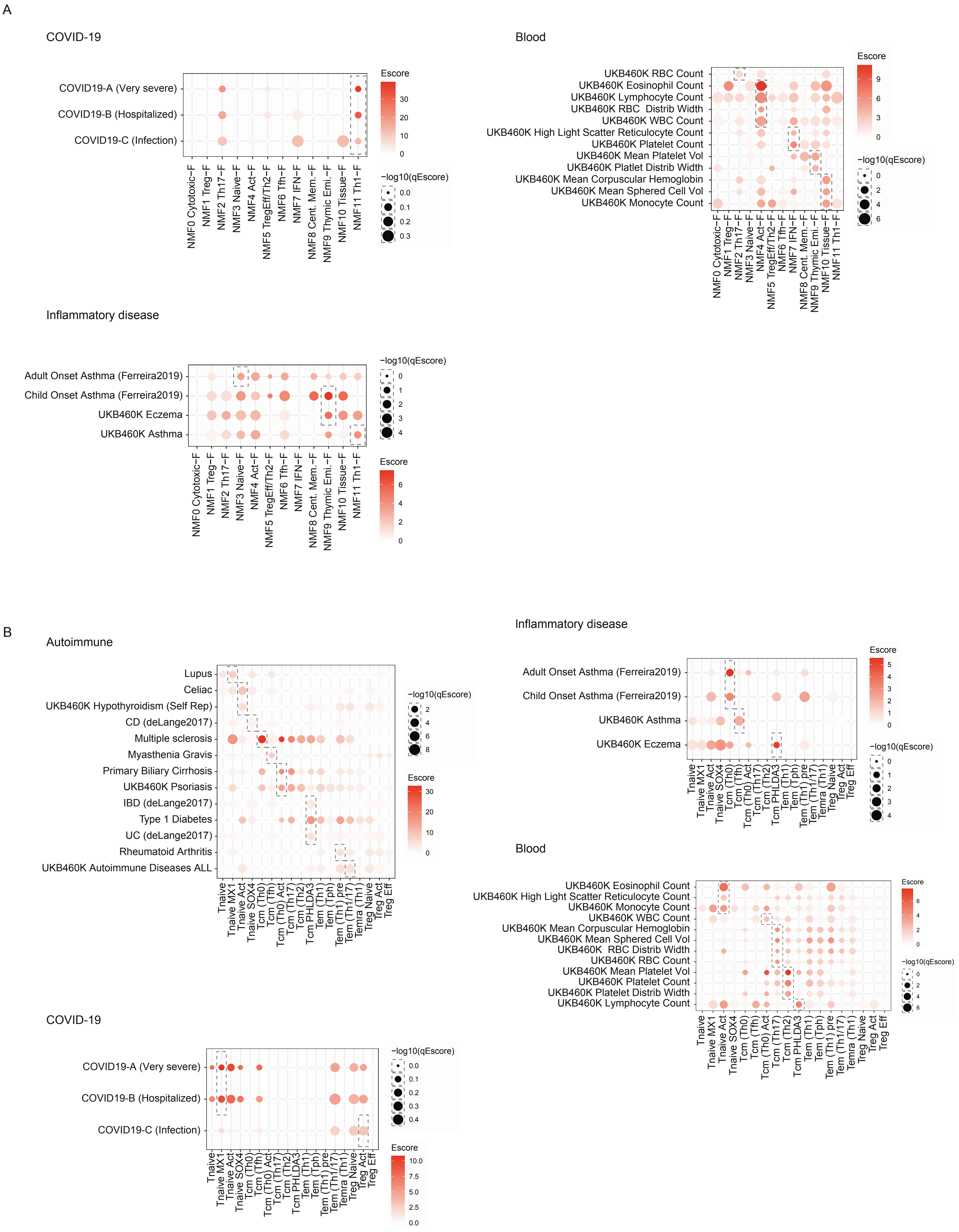
A
COVID-19
COVID19-A (Very severe)
COVID19-B (Hospitalized)
COVID19-C (Infection)
NMF0 Cytotoxic−F
NMF1 Treg−F
NMF2 Th17−F
NMF3 Naive−F
NMF4 Act−F
NMF5 TregEff/Th2−F
NMF6 Tfh−F
NMF7 IFN−F
NMF8 Cent. Mem.−F
NMF9 Thymic Emi.−F
NMF10 Tissue−F
NMF11 Th1−F
Escore
−log10(qEscore)
Blood
UKB460K RBC Count
UKB460K Eosinophil Count
UKB460K Lymphocyte Count
UKB460K RBC Distrib Width
UKB460K WBC Count
UKB460K High Light Scatter Reticulocyte Count
UKB460K Platelet Count
UKB460K Mean Platelet Vol
UKB460K Platlet Distrib Width
UKB460K Mean Corpuscular Hemoglobin
UKB460K Mean Sphered Cell Vol
UKB460K Monocyte Count
Inflammatory disease
Adult Onset Asthma (Ferreira2019)
Child Onset Asthma (Ferreira2019)
UKB460K Eczema
UKB460K Asthma
B
Autoimmune
Lupus
Celiac
UKB460K Hypothyroidism (Self Rep)
CD (deLange2017)
Multiple sclerosis
Myasthenia Gravis
Primary Biliary Cirrhosis
UKB460K Psoriasis
IBD (deLange2017)
Type 1 Diabetes
UC (deLange2017)
Rheumatoid Arthritis
UKB460K Autoimmune Diseases ALL
Tnaive
Tnaive MX1
Tnaive Act
Tnaive SOX4
Tcm (Th0)
Tcm (Tfh)
Tcm (Th0) Act
Tcm (Th17)
Tcm (Th2)
Tcm PHLDA3
Tem (Th1)
Tem (Tph)
Tem (Th1) pre
Tem (Th1/17)
Temra (Th1)
Treg Naive
Treg Act
Treg Eff
Inflammatory disease
Blood
UKB460K Eosinophil Count
UKB460K High Light Scatter Reticulocyte Count
UKB460K Monocyte Count
UKB460K WBC Count
UKB460K Mean Corpuscular Hemoglobin
UKB460K Mean Sphered Cell Vol
UKB460K RBC Distrib Width
UKB460K RBC Count
UKB460K Mean Platelet Vol
UKB460K Platelet Count
UKB460K Platelet Distrib Width
UKB460K Lymphocyte Count
COVID-19

Figure S13. Partitioned heritability, related to Figure 4

(A and B) Dot plots showing partitioned heritability of diseases across NMF gene features (A) or cell types (B). Duplicated traits were removed for the visualization. Full statistics are shown in Tables S11 and 12.

ID	Gender	Age	Disease
**STR4**	Female	31	HC
**STR5**	Male	50	HC
**STR21**	Male	27	HC
**MS01**	Male	36	MS
**MS02**	Female	23	MS
**MS03**	Female	39	MS
**MS04**	Male	24	MS
**MG01**	Male	72	MG
**MG02**	Male	51	MG
**MG03**	Male	46	MG
**SL02**	Female	58	SLE
**SL03**	Female	62	SLE
**SL04**	Male	44	SLE

Table S1. Patient information, related to Figure 1

The information of patients used in scRNA-seq experiments.
