# Supplementary material for: Single-cell transcriptome landscape of circulating CD4+ T cell populations in autoimmune diseases
Source: Cell Genom. 2024 Jan 3;4(2):100473. doi: 10.1016/j.xgen.2023.100473 (PMC10879034; doi:10.1016/j.xgen.2023.100473)
Supplement: Document S2. Supplementary Note [file mmc13.pdf]

Cell Genomics

Article

Single-cell transcriptome landscape of circulating $CD4^+$ T cell populations in autoimmune diseases

Graphical abstract

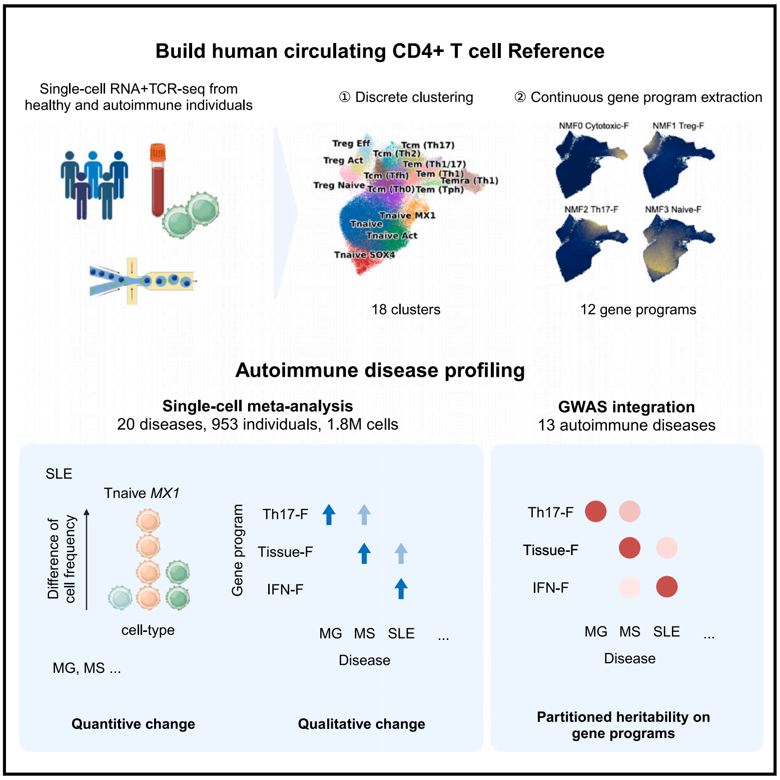

Highlights

- Identified 18 cell types and 12 gene programs from blood $CD4^+$ T cells
- Meta-analysis enrolling 1.8M cells outlines $CD4^+$ T alterations in autoimmune diseases
- Observed disease-specific heritability enrichment within $CD4^+$ T cell gene programs

Authors

Yoshiaki Yasumizu, Daiki Takeuchi, Reo Morimoto, ..., Hideki Mochizuki, Naganari Ohkura, Shimon Sakaguchi

Correspondence

nohkura@ifrec.osaka-u.ac.jp (N.O.), shimon@ifrec.osaka-u.ac.jp (S.S.)

In brief

Yasumizu, Takeuchi, Morimoto, et al. compile a comprehensive reference of circulating $CD4^+$ T cells, encompassing 12 gene programs and 18 clusters. They reveal disease-specific cellular characteristics through the integration of genome-wide association studies and single-cell meta-analysis. This work provides insights for precision medicine.

Yasumizu et al., 2024, Cell Genomics *4*, 100473
February 14, 2024 © 2023 The Authors.
https://doi.org/10.1016/j.xgen.2023.100473

CellPress

Cell Genomics

CellPress
OPEN ACCESS

Article

Single-cell transcriptome landscape of circulating CD4$^+$ T cell populations in autoimmune diseases

Yoshiaki Yasumizu,[1,2,3,13] Daiki Takeuchi,[1,4,13] Reo Morimoto,[1,13] Yusuke Takeshima,[1] Tatsusada Okuno,[2] Makoto Kinoshita,[2] Takayoshi Morita,[5] Yasuhiro Kato,[5,6] Min Wang,[7,8] Daisuke Motooka,[3,9] Daisuke Okuzaki,[3,9] Yamami Nakamura,[1] Norihisa Mikami,[1] Masaya Arai,[1] Xuan Zhang,[8] Atsushi Kumanogoh,[3,5,6,10] Hideki Mochizuki,[2,3] Naganari Ohkura,[1,11,14,*] and Shimon Sakaguchi[1,12,*]

[1]Department of Experimental Immunology, Immunology Frontier Research Center, Osaka University, Osaka, Japan
[2]Department of Neurology, Graduate School of Medicine, Osaka University, Osaka, Japan
[3]Integrated Frontier Research for Medical Science Division, Institute for Open and Transdisciplinary Research Initiatives (OTRI), Osaka University, Osaka, Japan
[4]Faculty of Medicine, Osaka University, Osaka, Japan
[5]Department of Respiratory Medicine and Clinical Immunology, Osaka University Graduate School of Medicine, Osaka, Japan
[6]Department of Immunopathology, Immunology Frontier Research Center, Osaka University, Osaka, Japan
[7]Clinical Immunology Center, State Key Laboratory of Complex Severe and Rare Diseases, Peking Union Medical College Hospital, Chinese Academy of Medical Sciences and Peking Union Medical College, Beijing, China
[8]Department of Rheumatology, Beijing Hospital, National Center of Gerontology, Institute of Geriatric Medicine, Chinese Academy of Medical Sciences, Beijing, China
[9]Genome Information Research Center, Research Institute for Microbial Diseases, Osaka University, Osaka, Japan
[10]Center for Infectious Diseases for Education and Research, Osaka University, Osaka, Japan
[11]Department of Frontier Research in Tumor Immunology, Graduate School of Medicine, Osaka University, Osaka, Japan
[12]Department of Experimental Immunology, Institute for Life and Medical Sciences, Kyoto University, Kyoto, Japan
[13]These authors contributed equally
[14]Lead contact
*Correspondence: nohkura@ifrec.osaka-u.ac.jp (N.O.), shimon@ifrec.osaka-u.ac.jp (S.S.)
https://doi.org/10.1016/j.xgen.2023.100473

SUMMARY

CD4$^+$ T cells are key mediators of various autoimmune diseases; however, their role in disease progression remains unclear due to cellular heterogeneity. Here, we evaluated CD4$^+$ T cell subpopulations using decomposition-based transcriptome characterization and canonical clustering strategies. This approach identified 12 independent gene programs governing whole CD4$^+$ T cell heterogeneity, which can explain the ambiguity of canonical clustering. In addition, we performed a meta-analysis using public single-cell datasets of over 1.8 million peripheral CD4$^+$ T cells from 953 individuals by projecting cells onto the reference and cataloging cell frequency and qualitative alterations of the populations in 20 diseases. The analyses revealed that the 12 transcriptional programs were useful in characterizing each autoimmune disease and predicting its clinical status. Moreover, genetic variants associated with autoimmune diseases showed disease-specific enrichment within the 12 gene programs. The results collectively provide a landscape of single-cell transcriptomes of CD4$^+$ T cell subpopulations involved in autoimmune disease.

INTRODUCTION

Autoimmune disorders encompass various conditions where the immune cells display abnormal reactivity toward normal tissues. These diseases are multifactorial, polygenic, and prevalent, affecting 3%–5% of the population.[1,2] Numerous studies have highlighted the role of CD4$^+$ T cells in the onset or exacerbation of these diseases.[2,3]

CD4$^+$ T cells exhibit a variety of states (e.g., naive, memory), polarizations (e.g., Th1, Th2, Th17, T follicular helper or Tfh), and also include a distinct subpopulation engaged in the maintenance of self-tolerance and homeostasis (regulatory T cells or Tregs).[4,5] While a great deal of effort has been devoted to the detailed classification of CD4$^+$ T cells, the complete picture of heterogeneity and its relationship to diseases is still controversial. Furthermore, making consistent assessments across reports is challenging since these reports were based on inconsistent cellular classifications.

The recent emergence of single-cell analysis has greatly contributed to the elucidation of cellular diversities through unbiased profiling.[6–11] In addition, single-cell RNA sequencing (scRNA-seq) is suitable for robust cross-dataset data integration,

© 2023 The Authors.
This is an open access article under the CC BY license (http://creativecommons.org/licenses/by/4.0/).

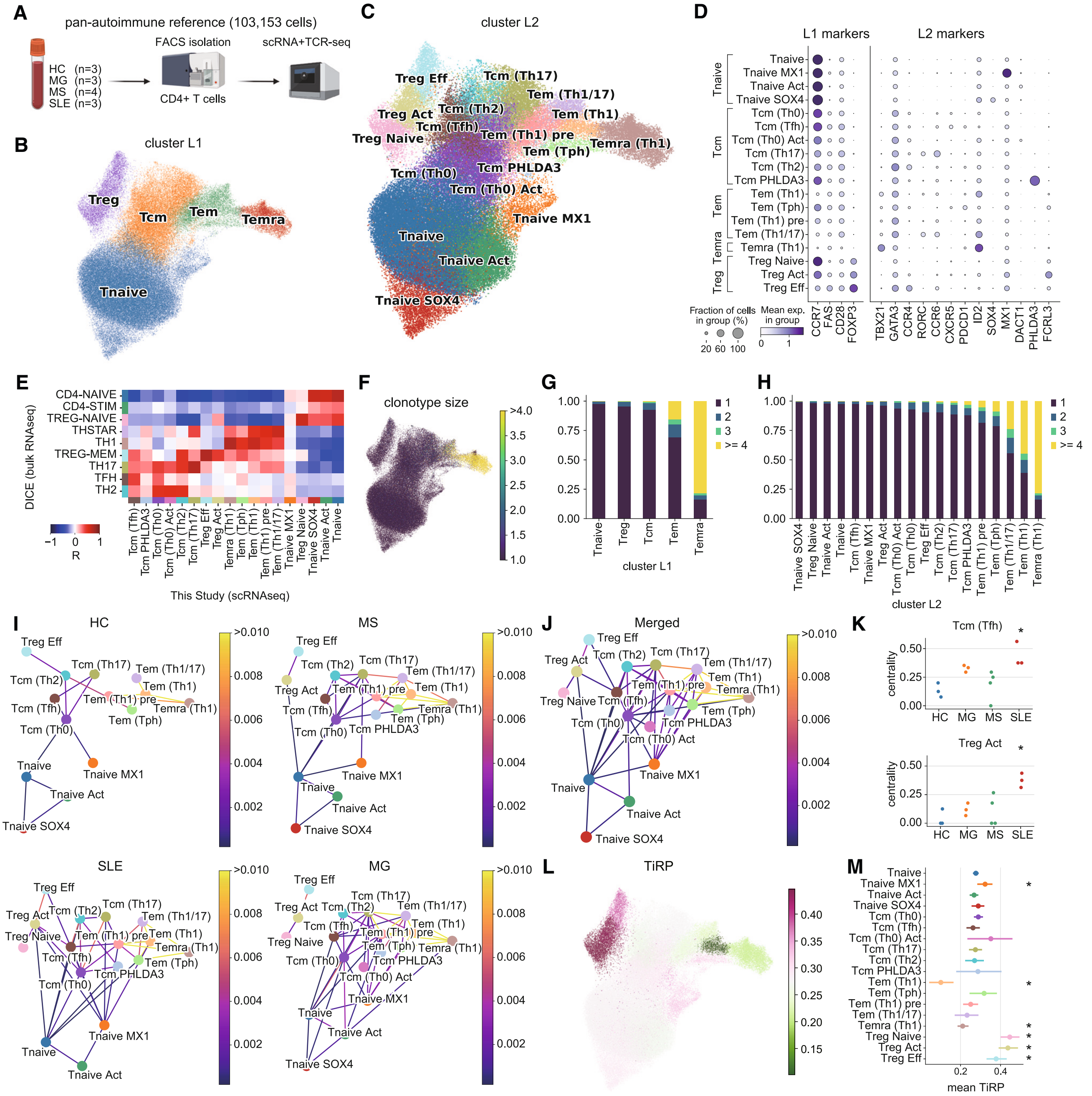

Figure 1. Global profiling of CD4⁺ T cells

(A) Sample collection strategy. HC, healthy control; MG, myasthenia gravis; MS, multiple sclerosis; SLE, systemic lupus erythematosus.

(B and C) Cluster layer 1 (L1) and layer 2 (L2) on Uniform Manifold Approximation and Projection (UMAP) embeddings.

(D) Dot plot depicting signature genes' mean expression levels and percentage of cells expressing them across clusters. Marker genes for the plot were manually selected. See also Figure S1C for automatically extracted marker genes.

(E) Expression correlation of clusters with bulk RNA-seq for sorted $CD4^+$ T cell fractions generated by the DICE project.

(F) UMAP plot showing clonotype size. The color indicates the number of cells sharing the same T cell receptor (TCR) sequences.

(G and H) Clonotype size distributions across clusters.

(I and J) TCR similarity networks in autoimmune patients, healthy donors (I), and all donors (J). TCR similarity was calculated for each sample, and only edges where overlapping clonotypes were detected in ≥ 2 (I) or ≥ 4 (J) samples are depicted as robust overlaps. The edge color indicates the average TCR similarity of all samples.

(legend continued on next page)

allowing large-scale investigations.[12–15] Furthermore, in the field of T cell research, a number of tools have enabled precise T cell profiling across various contexts such as chronic infection and tumor-infiltrating T cells.[16,17] On the other hand, conventional clustering and marker gene detection strategies for single-cell analysis possess the following weaknesses: (1) cell fraction definition requires arbitrary boundaries, (2) marker genes for clusters can be occupied by redundant genes or uninterpretable genes, such as long noncoding or ribosomal genes, due to the influence of larger cell population structures, and (3) pairwise differentially expressed gene detection cannot capture global gene variation across multiple clusters. Several studies have attempted to tackle these issues.[18,19] Especially, in some previous reports, non-negative matrix factorization (NMF)[20] has been utilized to extract both cell-type identity and cellular activity programs, thereby addressing the profiling of complex cell populations.[21,22] However, it still harbored complexity in determining the parameters and in the flexible integration between different datasets.

Here, we construct a consensus reference for CD4$^+$ T cells in peripheral blood from autoimmune and healthy individuals covering various inflammatory conditions. The reference consists of 18 cell types defined by a conventional clustering strategy and 12 transcriptomic gene programs extracted by conducting decomposition using NMF without boundaries, which overcame the weakness of existing clustering-based single-cell analyses. The results show that diverse CD4$^+$ T cell features were formed by a combination of 12 independent gene programs. We also illustrate that the gene features obtained by NMF could be projected to other bulk/scRNA-seq data to help interpret various datasets. Using these frameworks to examine the genetic contribution and subsequent changes of CD4$^+$ T cells in autoimmunity, we performed a meta-analysis that enrolled over 1.8 million CD4$^+$ T cells using published single-cell data of 20 diseases and integrated genome-wide association study (GWAS) statistics for 180 traits with our dataset. These analyses provided a full picture of CD4$^+$ T cells in autoimmune diseases from the perspective of phenotypes and genetics.

RESULTS

Single-cell profiling of peripheral CD4$^+$ T cells from healthy and autoimmune donors

To characterize CD4$^+$ T cells in various autoimmune properties, we performed scRNA-seq and T cell receptor (TCR)-seq using droplet-based single-cell isolation technology and profiled CD4$^+$ T cells, which were collected from three healthy donors, three myasthenia gravis (MG) patients, four multiple sclerosis (MS) patients, and three systemic lupus erythematosus (SLE) patients (Figures 1A–1D; Table S1). After quality control (QC), 103,153 cells were retained and used for the downstream analyses. As the primary layer of clustering (cluster L1), we identified a dynamic differentiation from a naive state via an effector state to a terminally differentiated state. In cluster L1, CD4$^+$ naive T cells (Tnaive) (*CCR7*$^+$ *FAS*$^-$), CD4$^+$ central memory T cells (Tcm) (*CCR7*$^+$ *FAS*$^+$), CD4$^+$ effector memory T cells (Tem) (*CCR7*$^-$ *FAS*$^+$), and CD4$^+$ terminally differentiated effector memory T cells (Temra) (*FAS*$^+$ *CD28*$^-$) were observed with distinct gene expression patterns (Figures 1B, 1D, and S1A; Table S2). Tregs were also observed as a distinct cluster with the expression of the master regulator *FOXP3*. Next, we further divided the cells into 18 clusters as the secondary layer, cluster L2 (Figures 1C and S1B–S1D; Table S3). For example, we broke down cluster L1 cells into several T cell subclusters according to well-known transcription factors and chemokine receptors such as Tcm cells into Tfh (Tfh; *CXCR5*, *PDCD1*), Th2 (*GATA3*, *CCR4*), Th17 (*RORC*, *CCR6*); Tem cells into Th1/17 (*TBX21*/Tbet, *RORC*), Th1 (*TBX21*/Tbet); Temra cells into Th1 (Figures 1C, 1D, and S1A–S1D). Treg cells were divided into three clusters; Treg Naive (*CCR7*), Treg Activated (*ID2*), and Treg Effector (*CCR4*) (Figures 1C, 1D, and S1A–S1D). In addition, several minor clusters were found, such as Tnaive *MX1*, which preferentially expresses interferon signature genes (Figures 1C, 1D, S1A, S1C, and S1D). Transcriptome profiles of each cluster were concordant with bulk RNA-seq data from fluorescence-activated cell sorting-sorted CD4$^+$ T cell fractions provided by the Database of Immune Cell Expression, Expression quantitative trait loci and Epigenomics (DICE) project[23] (Figure 1E). Furthermore, based on the consistent expression of marker genes across all samples, we confirmed that the batch correction by Harmony was performed appropriately (Figures S1E and S1F). We found a *CXCR5*$^-$ *PDCD1*$^+$ cluster occupying 1% in CD4$^+$ T cells whose marker genes corresponded to the canonical marker for T peripheral helper (Tph) cells[24–26] in Tem (Figures S1A and S1G). The population was annotated as circulating Tph, although a few cells with the expression *CXCR5*$^-$ *PDCD1*$^+$ were also observed in broader populations such as Tcm and Temra (Figures S1A, S1C, and S1H). Overall, we identified cell populations of peripheral CD4$^+$ T cells from healthy and autoimmune states using scRNA-seq.

TCR features across CD4$^+$ T cells reflect cellular properties

Because TCR responses shape T cell functions and differentiation, TCR diversities and overlaps provide useful information for the properties and relationships of populations. Therefore, we analyzed single-cell TCR features sequenced along with gene expression. Clonotype sizes and diversity across cluster L1 populations revealed that Temra was most clonally expanded, followed by Tem, Tcm, Treg, and Tnaive (Figures 1F and 1G). Similarly, in cluster L2 populations, Temra (Th1), Tem (Th1), and Tem

(K) Degree centrality of TCR networks. Significance across clusters was calculated by one-way analysis of variance and, after multiple test corrections by false discovery rate, Tcm (Tfh) and Treg Act were retained as significant cell types. Then, pairwise Tukey-HSD post hoc tests were performed. *$p_{adj} < 0.05$ in comparison with HCs.

(L) TCR-intrinsic regulatory potential (TiRP) score distributions on UMAP plot. Mean scores for each cluster L2 were shown.

(M) TiRP score distribution across cluster L2. The dot shows the mean, and the confidence interval (CI) shows 95% CI of the bootstrap distribution of means (n = 1,000). Adjusted p values of significant clusters, Tnaive *MX1* 1.29×10^{-2}, Tem (Th1) 5.82×10^{-11}, Temra (Th1) 4.84×10^{-22}, Treg Naive 2.14×10^{-13}, Treg Act 2.14×10^{-13}, Treg Eff 3.82×10^{-5} (two-sided Mann-Whitney U test was performed for one cluster vs. the other clusters iteratively).

(Th1/17) possessed a limited number of clonotypes, whereas Tnaive and Treg Naive maintained diverse clonotype pools (Figure 1H). The TCR similarity network showed a repertoire sharing between neighboring clusters, Tnaive and Tcm, Tcm and Tem, and Tem and Temra, while the distal connection such as from Tnaive to Temra was not observed, suggesting stepwise development from Tnaive to Tcm, Tem, and Temra (Figures 1I, 1J, and S2B). The repertoires were also mutually shared within Tcm cell populations, suggesting the plasticity of T cell polarization against the same epitopes. In addition, no substantial overlap was observed between the repertoires of Treg Naive and conventional T cells (Tconvs), whereas Treg Act and Treg Eff demonstrated shared repertoires with Tcm populations (Figures 1I, 1J, and S2B). We also measured the centrality of TCR networks for each cell type to evaluate the differentiation potential of each cluster. The centrality of Tcm (Th0), Tem (Th1) pre, and Tnaive were consistently high, suggesting that these cells possess the possibility to differentiate into a variety of cell types (Figure S2C). In addition, TCR networks differed depending on the disease states (Figure 1I). Especially the centrality of Tcm (Tfh) and Treg Act were higher in SLE (Figures 1K and S2D). These results indicated that the kinetics of $CD4^+$ T cell differentiation varied depending on the disease state.

Previous studies have shown that T cells with stronger TCR stimulation within the thymus are more likely to differentiate into Tregs than Tconvs.[27] Therefore, Tregs have specific TCR properties, such as hydrophobicity in complementary determining region 3 regions.[28] We measured the Tregness of TCRβ chains (TCR-intrinsic regulatory potential [TiRP] score[28]) and found that the mean TiRP score was higher in Treg cells compared with Tconv cells (Figures 1L and 1M). On the contrary, Tem (Th1) showed a low TiRP score, indicating that Tem (Th1) has experienced stimulation with non-self antigens. Among Treg cells, Treg Naive and Treg Act showed higher TiRP scores than Treg Eff. It has been thought that naive Tregs contain predominantly thymic differentiated Tregs (tTregs), while effector Tregs are compensated by peripherally differentiated Tregs in addition to tTregs.[29] This notion was concordant with our observations that naive Tregs had the strongest Treg characteristics in the TCRs and that Treg Act and Eff shared TCRs with Tconvs (Figures 1I, 1J, and S2E). Furthermore, in MS patients, the TiRP scores of the Treg Act were significantly low, reflecting disease-dependent Treg compensation by Tconvs (Figure S2F). Overall, TCR repertoires provided valuable insights into T cell characteristics and relationships during the differentiation.

Decomposition of cellular programs using NMF

Next, we attempted to identify cellular programs within and across cell types. We noticed that conventional clustering and marker gene detections could fail to capture meaningful clusters and genes. For example, differentially expressed genes in our reference included overlapping genes among Th1 cell populations and nonsense genes in Tnaive cells, suggesting that the conventional marker gene detection is insufficient for $CD4^+$ T cells (Figure S1C). We suspected that artificially delineating in the clustering process is unsuitable for a gradual population such as $CD4^+$ T cells. In addition, because marker gene detections are performed by pairwise comparison, global representations across cell types cannot be detected. To overcome these limitations, we applied non-negative matrix factorization[20] to normalized gene expression of our scRNA-seq data. NMF and its derivative method, consensus NMF (cNMF),[22] have been widely used in single-cell analysis as a technique to extract both cell-type identity and cellular activity as gene programs. NMF unbiasedly dissected gene expression profiles into a gene feature matrix ***W*** and a cell feature matrix ***H*** (Figure 2A). To determine the number of components, we assessed the explained variances and maximum inter-component correlations and selected 12 for the number of components as they kept sufficient information and were not redundant (Figure S3A; STAR Methods). Based on the gene feature profiles and the enriched pathways, we annotated the NMF components (Figures 2B–2D and S3B; Tables S4 and S5). Several factors were related to T cell polarization, such as Treg-Feature (Treg-F) (NMF1; genes with high weights; *IKZF2*, *FOXP3*), Th17-F (NMF 2; *RORC*, *CCR6*), TregEff/Th2-F (NMF5; HLA class II genes, *CCR10*, *CCR4*), Tfh-F (NMF6; *TIGIT*, *CXCR5*), Th1-F (NMF11; *GZMK*, *EOMES*, *CXCR3*), and differentiations such as Naive-F (NMF3; *CCR7*, *TCF7*), Central Memory-F (NMF8; *S100A8*, *ANXA1*), and Cytotoxic-F (NMF0; *GZMB*, *NKG7*). NMF5 was enriched in both Th2 and Treg Eff, suggesting that effector Treg cells and Th2 cells may be controlled by the shared program as previously suggested[30] (Figure 2B). NMF6 (Tfh-F) also demonstrated moderate activity in Treg Act, suggesting an overlap between Treg Act and T-follicular regulatory (Tfr) cells[31] (Figure 2B). $NMF11^{high}$ cells were enriched in Tem (Tph), Tem (Th1), and Tem (Th1/17) cells, showing a wide range of Th1ness gene usage across these subtypes. Moreover, NMF7 was a type I interferon signature gene component enriched in Tnaive *MX1* (Figures 1C and S3B). Intriguingly, NMF10 captured a global feature across cell types consisting of AP-1 family genes (*JUNB*, *FOS*), *NFKBIA*, *CD69*, and *CXCR4* (Figure 2D). This feature was concordant with tissue-homing T cells observed in the thymoma of MG patients[7] and the central nervous system of neurodegenerative disease patients,[32] and was labeled as Tissue-F. NMF4 (Act-F) was related to *IL7R* signaling, which is an essential survival and differentiation signal.[33] The proportion of explained variance (Evar) showed the most drastic variations in the peripheral $CD4^+$ T cells were differentiation from Tnaive to Tcm, Tem, and Temra, and the polarizations were relatively smaller changes and independent of the differentiation programs (Figure 2B). Altogether, NMF succeeded in the decomposition of peripheral $CD4^+$ T cell gene programs into 12 components and showed that complex $CD4^+$ T cell populations were represented by a simple combination of the 12 components.

NMF projection enables fast interpretation of various $CD4^+$ T transcriptome datasets

One of the biggest challenges in single-cell analysis is the integration of datasets. To achieve a simple integration, we expanded the NMF framework to allow the projection of the pre-computed gene feature matrix onto other datasets by developing a bioinformatics tool, NMFproj (Figure 2A, https://github.com/yyoshiaki/NMFprojection). Recognizing the prevalent use of cNMF, we also designed NMFproj to be directly compatible with cNMF outputs. To measure how the NMF features explain

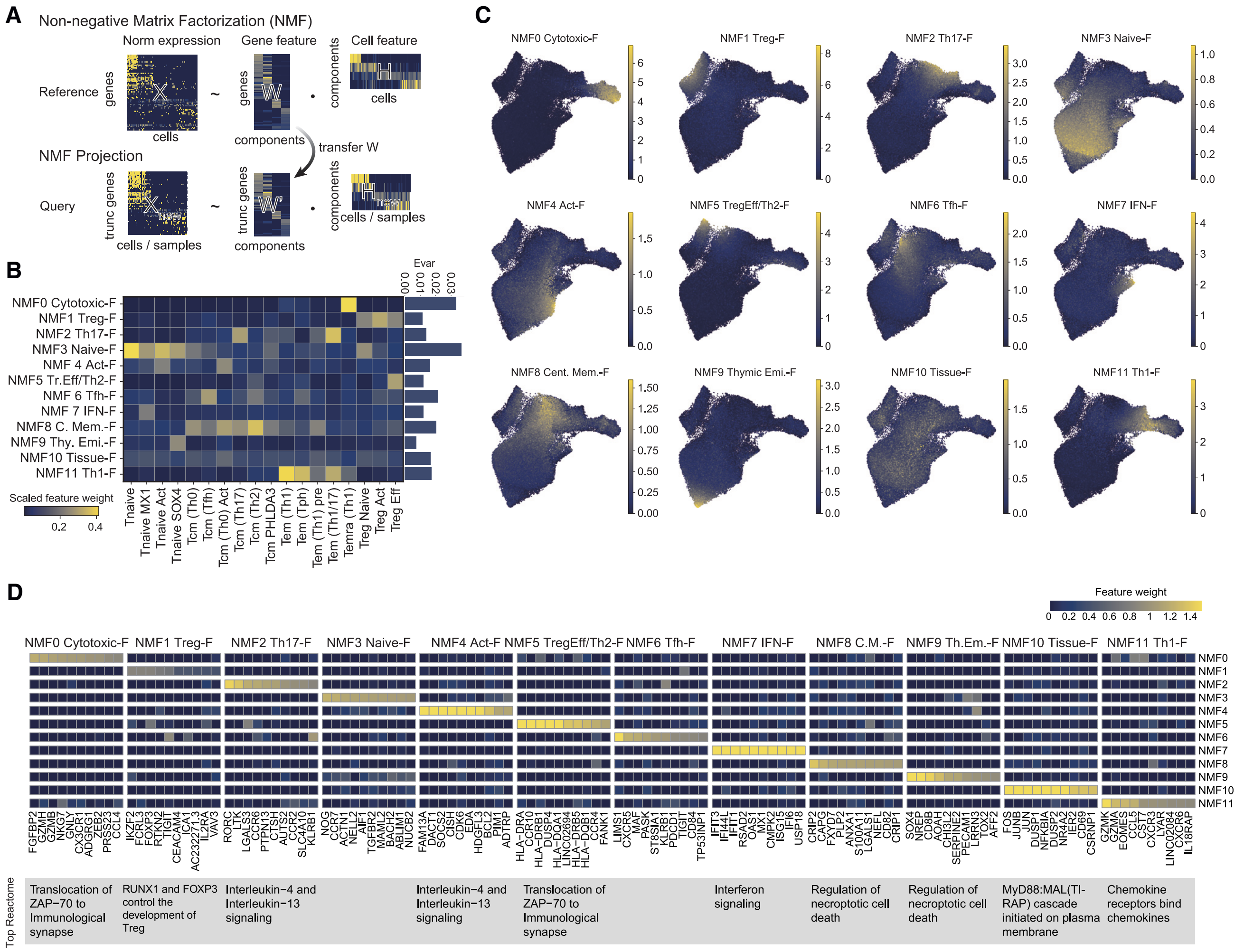

Figure 2. NMF captured 12 CD4+ T cell features

(A) Schematic view of NMF and NMF projection (NMFproj).

(B) Matrixplot showing the mean scaled NMF feature weight for each cluster L2 population. The explained variance (Evar) is also shown on the right. The NMF feature weight is scaled by the maximum value for each feature for visualization.

(C) NMF cell feature value on UMAP plots.

(D) Gene features for each component. The top 10 genes for each feature were selected. The 12 gene features are annotated using top genes and previous reports as NMF0 Cytotoxic-Feature (F), NMF1 Treg-F, NMF2 Th17-F, NMF3 Naive-F, NMF4 Activation-F (Act-F), NMF5 TregEff/Th2-F, NMF6 Tfh-F, NMF7 IFN-F, NMF8 Central Memory-F, NMF9 Thymic emigrant-F, NMF10 Tissue-F, and NMF11 Th1-F. The Reactome pathway with the smallest p value for each gene feature is shown below the heatmap (Figure S3B; Table S5).

the variance of the query dataset, we introduced a QC metric named the proportion of overlapped highly variable genes (POH) and Evar for all factors (Evar all) (Figure S3C). A low POH indicates that the query dataset has much variability other than the NMF features evaluated by NMFproj. We applied NMFproj to various datasets to validate the scalability (supplemental note). Analysis of bulk RNA-seq of sorted peripheral CD4+ T cells provided by the DICE project[23] demonstrated that each fraction was well represented by the 12 NMF gene features (POH: 0.272; Evar all: 0.839, Figure S3D). Miyara classification[34] categorizes CD4+ T cells into six distinct fractions (Fr. I to Fr. VI) based on the expression of CD45RA and CD25. This classification enables the distinction between Tregs and Tconvs, as well as naive and activated cell states. However, the identity of borderline populations, such as Fr. III (CD45RA− CD25int), remains less understood. Using NMFproj, we re-evaluated this classification and found that Fr. III possesses Th17 type characteristics in line with the original report[34] (POH: 0.542; Evar all: 0.823, Figure S3E; supplemental note). In addition, profiling of circulating Tph cells[24] revealed that Tph cells possessed both NMF6 (Tfh-F) and NMF11 (Th1-F) in concordance with Tem (Tph) we defined as cluster L2 (POH: 0.134; Evar all: 0.816, Figure S3F; supplemental note). We also attempted to utilize NMFproj for the QC of *in vitro* induced Treg (iTreg) cells of mouse[35] and found that iTreg+ cells induced in optimized conditions for enhancing Treg functionality showed higher NMF1

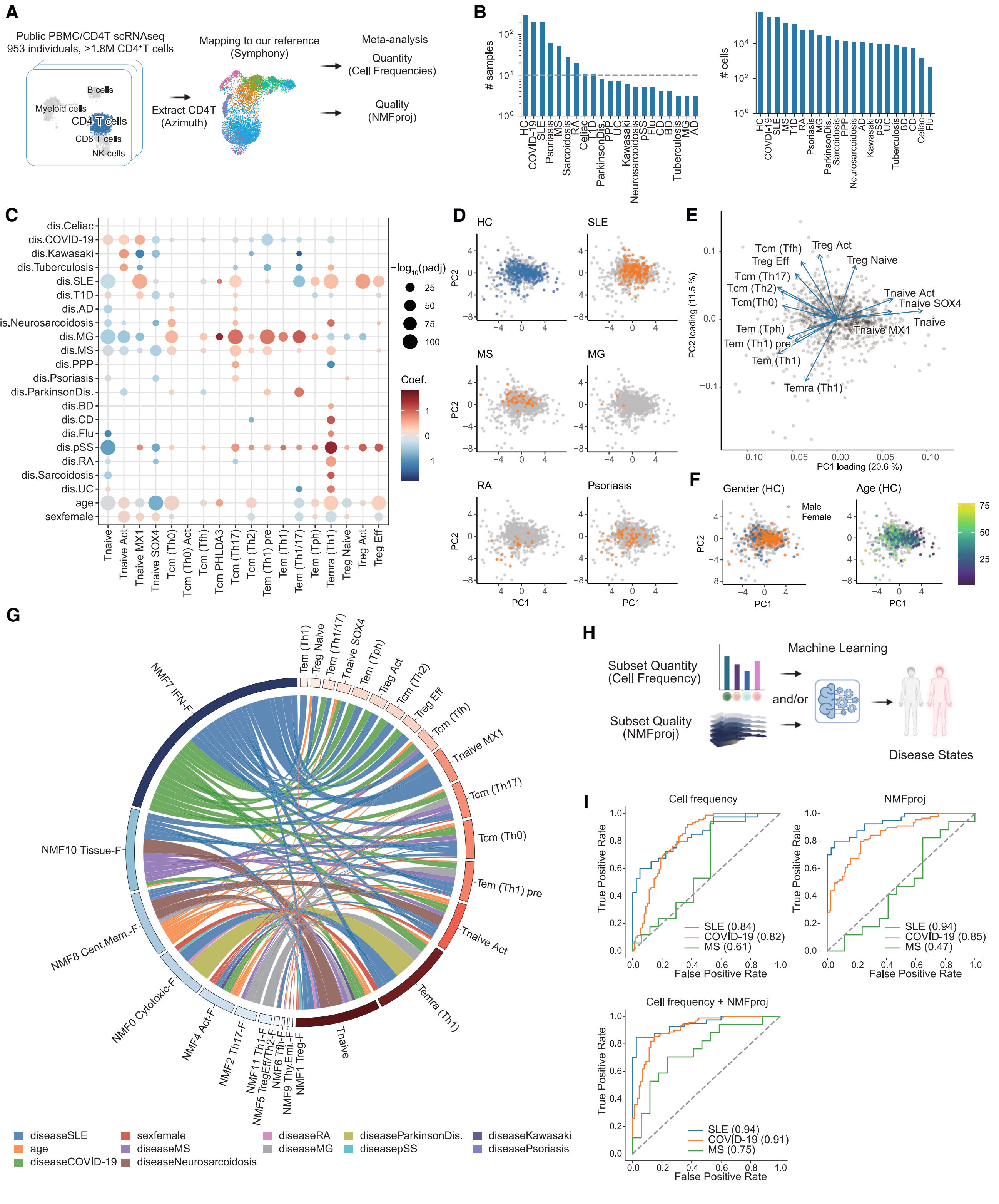

Figure 3. Pan-autoimmune meta-analysis of peripheral CD4$^+$ T cells

(A) Strategy for meta-analysis of peripheral CD4$^+$ T cells across diseases. First, CD4$^+$ T cells were extracted from peripheral blood mononuclear cell (PBMC) scRNA-seq datasets using Azimuth.[62] Extracted CD4$^+$ T cells were mapped on our reference using Symphony[14] with a batch correction. Mapped cells were used to assess cell frequency and NMF cell features for each cluster.

(legend continued on next page)

(Treg-F) values than conventional iTreg cells (POH: 0.182; Evar all: 0.844, Figure S3G; supplemental note). We also applied NMFproj to scRNA-seq datasets of cross-tissue immune cells[36] (Figure S6, POH: 0.560; Evar all: 0.332 in CD4$^+$ T cells, supplemental note), pan-cancer tumor-infiltrating CD4$^+$ T cells[15] (Figure S4A, POH: 0.530; Evar all: 0.338, supplemental note), and mouse splenocytes[37] (Figure S4B, POH: 0.394; Evar all: 0.146, supplemental note). CD4$^+$ T programs defined by circulating CD4$^+$ T cells were adequately projected to various conditions, with permissive POH values and lower Evar all. Given the variance observed in Evar all between circulating and tissue-derived datasets, we sought to explain this by comparing the *ab initio* NMF in a pan-cancer dataset with the NMF defined in circulating CD4$^+$ T cells. We found that the majority of circulating CD4$^+$ T factors had correlating factors in the NMF defined by the CD4$^+$ tumor-infiltrating lymphocyte (TIL) data (Figures S5A–S5D). For example, NMF7 (IFN-F) in circulating CD4$^+$ T corresponded to NMF15 in *ab initio* NMF defined by the pan-cancer dataset (Figures S4A and S5D). However, NMF8 (Cent. Mem.-F) and NMF4 (Act-F), which were broadly elevated or almost inactive within the tumor, were not included in the NMF defined for TILs. In addition, some factors, including NMF17, which contains *CXCL13* and *IL21*, were only included in NMF components defined by TILs (Figure S5D). From these results, it seems that the NMF defined in circulating CD4$^+$ T cells can be correctly projected by NMFproj even to TILs, but some tissue-specific factors may need to be carefully evaluated. Furthermore, cell-specific qualitative changes have been reported in autoimmune diseases, such as Treg dysfunction in SLE,[38] and we hypothesized that NMFproj could be used to detect these changes in individual cell populations. To test this, we applied NMFproj to bulk RNA-seq data of sorted peripheral CD4$^+$ T cell fractions from various autoimmune patients.[39] NMFproj detected a subset-specific gene program robustly even in a variety of autoimmune disease conditions (Figure S7A; supplemental note). The results showed cell-type-wide enhancement of NMF7 (IFN-F) in SLE and mixed connective tissue disease and hampered NMF1 (Treg-F) in Fr.I nTregs (CD45RA$^+$ CD25$^+$) in SLE patients as previously reported[38] (Figure S7B). These results indicated that NMFproj could robustly assess the qualities of CD4$^+$ T cells in various tissues and disease states, regardless of bulk/single cell or human/mouse. We were also able to perform analyses while maintaining interpretability in a large dataset spanning multiple diseases and cell types.

Meta-analysis of CD4$^+$ T cells in various autoimmune diseases

To extend CD4$^+$ T cell profiling to various autoimmune and infectious diseases, we performed a meta-analysis using publicly available single-cell data.[6,8,40–61] We integrated publicly available datasets with two strategies: (1) quantitative evaluation of cell frequencies by mapping to our reference and (2) evaluation of qualitative changes per cell type using NMFproj. We extracted CD4$^+$ T cells from peripheral blood mononuclear cells (PBMCs) using Azimuth[62] and then mapped them to our reference using Symphony[14] (Figure 3A, the pipeline is available at https://github.com/yyoshiaki/screfmapping). Reference mapping using Symphony achieved accurate label transfer at both cluster L1 and L2 resolutions (Figures S8A–S8D, accuracy = 0.923 and 0.781, adjusted Rand index = 0.825 and 0.631 in cluster L1 and L2, respectively), and the result was concordant with those by other tools, CellTypist[36] and ProjecTILs[16] (Figures S8E and S8F). The suitability of Symphony mapping for conditions such as infectious diseases was also verified using silhouette scores (Figure S8G). We collected 1,809,668 CD4$^+$ T cells collected from 647 cases and 306 controls from 25 projects (Figures 3B and S9A; Table S6). For quality assurance, only datasets in which both HC and patients were present and at least three cases were included were used. As a prominent change, Tnaive decreased and Temra increased in various autoimmune diseases (Figure S9B; Table S7). It has been reported that Temra increased in the peripheral blood of rheumatoid arthritis (RA), MS, ulcerative colitis (UC), and Crohn's disease (CD) patients,[63–66] which was consistent with the present data. Kawasaki disease and type 1 diabetes (T1D) were exceptions among autoimmune diseases, with a slight increase in Tnaive and no significant change in Tcm, Tem, and Temra (Figure S9B), as reported previously.[67,68] At cluster L2 resolution, we found that Tnaive *MX1* increased in COVID-19, SLE, T1D, and primary Sjögren syndrome (pSS) patients (Figure 3C). The type I IFN response is essential for viral elimination and has been reported to be associated with COVID-19 pathology[69] and also known to be

(B) Bar plots showing the number of samples (left) and the number of CD4$^+$ T cells (right) enrolled in the meta-analysis. The dashed line in the left plot indicates a sample size of 10. Celiac, celiac disease; Kawasaki, Kawasaki disease; T1D, type 1 diabetes; AD, Alzheimer's disease; PPP, palmoplantar pustulosis; ParkinsonDis., Parkinson's disease; BD, Behçet's disease; CD, Crohn's disease; Flu, influenza; pSS, primary Sjögren syndrome; RA, rheumatoid arthritis; UC, ulcerative colitis.

(C) Dot plot showing changes in cell frequency at cluster L2 resolution. Dot colors show coefficients, and sizes show the significance of the generalized linear model (GLM) (STAR Methods). Detailed statistics can be found in Table S8. Only significant dots (p_{adj} < 0.05) are shown.

(D–F) Principal-component analysis (PCA) plots of samples based on cell frequencies. Sample distributions for each disease state (D), loading vectors for each cell type (E), and sample characteristics in healthy donors (F) are shown.

(G) Chord diagram showing the top 100 significant associations with positive coefficients between NMF features and cells in each condition, calculated by GLM (STAR Methods). Detailed statistics are shown in Table S9. The thickness of edges indicates the coefficient of GLM, and colors indicate conditions such as diseases, gender, and age.

(H) Strategy for predicting autoimmune states from CD4$^+$ T cell profiles using machine learning framework. As the input parameters, one model took only cell frequency, age, and gender (without NMFproj), while the other took cell frequency, NMF cell features in Tcm (Th0) and Tnaive, age, and gender (with NMFproj).

(I) Receiver operating characteristic curves of logistic regression models trained by cell frequencies (top left), by NMFproj values in Tnaive and Tcm (Th0) (top right), and both cell frequencies and NMFproj values (bottom). SLE, COVID-19, and MS patients were trained on 159, 116, and 35 patients with the same number of healthy subjects, and evaluated on 40, 89, and 17 patients and the same number of healthy subjects from independent datasets. Numbers in parentheses indicate the area under the curve (AUC).

associated with SLE,[70] pSS,[71] and T1D.[72] Our meta-analysis could detect these effects as the increase of Tnaive *MX1*. Moreover, in our meta-analysis, Tcm (Th17) was increased in various diseases, including previously reported diseases such as MG,[73] MS,[74,75] and psoriasis.[76] Regarding Tregs, we reported that Fr. II (CD45RA$^-$ CD25$^+$) is increased in sarcoidosis, while Fr. I and III are increased in active SLE.[34] Another group reported an increase of Tregs in pSS.[77] In concordance with these observations, Treg increased in SLE, neurosarcoidosis, sarcoidosis, and pSS, especially for Treg Eff in neurosarcoidosis and for Treg Act and Treg Eff in SLE patients (Figures 3C and S9B). Interestingly, the acute infection response to COVID-19 showed an increase in Tnaive, whereas influenza infection showed an increase in Temra. We also found age-dependent Tnaive decrease and Temra, Treg Eff increases concordant with previous reports[78] (Figure 3C). Sex differences in immunity are critical, especially for autoimmune diseases, because 80% of autoimmune disease occurs in females.[79] Previous reports have addressed several changes in females, such as the increase of recent thymic emigrants[80] and greater activation responses by *in vitro* stimulation.[81] As for gender differences, we observed a decrease in Tcm (Th2), Tem (Th1/17), Temra (Th1), and Treg Eff and an increase in Tnaive Act, Tnaive *MX1*, Tnaive *SOX4*, Tem (Tph), and Treg Naive in females. These alterations depending on disease, gender, and age were also observed as specific distributions on the principal-component analysis plot (Figures 3D–3F, S9C, and S3D). Overall, we profiled numerical features of CD4$^+$ T cells in broad autoimmune status, age, and gender.

Next, to measure the quality changes in autoimmune diseases, we applied NMFproj to the datasets and investigated NMF cell feature changes in each cluster L2 population (Figures 3G, S10A, and S11; Table S9). To add measurements for the biological significance, we also calculated a scaled intercept as the baseline gene program activity (Figure S11). The strongest skews were enriched in NMF7 (INF-F) in SLE and COVID-19 patients in a cell-type-wide manner. We also found that even neutral populations such as Tnaive, Tnaive (Act), and Tcm (Th0) showed disease-specific propensities. For example, NMF0 (Cytotoxic-F) increased in RA, MS, and pSS, NMF10 (Tissue-F) was increased in MS, COVID-19, SLE, and neurosarcoidosis, while NMF3 (Naive-F) decreased in a broad range of autoimmune diseases. In Treg cells, NMF1 (Treg-F) decreased in T1D, MG, and MS, indicating the dysfunction of Treg in these diseases independently of the number of Treg cells. In particular, for T1D and MS, where the sample size was large, we investigated the differentially expressed genes in each Treg subtype in each diseases and examined the genes with high feature values for NMF1 (Treg-F) (Figure S10B). Variations in the NMF1 (Treg-F)-related genes were most pronounced in Treg Eff, with T1D showing a decrease in functional molecules such as *IL2RA*, *TNFRSF1B*, and Treg regulators such as *FOXP3*, *IKZF2*, while MS showed a decrease in *IL2RA*, *IL10RA*, *IKZF4*, along with an increase in *CTLA4*, which is one of the prominent Treg effector molecules. Age-dependent increases of NMF8 (Cent. Mem.-F) and NMF4 (Act-F) were also observed. In females, NMF4 (Act-F) was enhanced broadly. Other findings, such as the upregulation of an activation molecule, CD69, which was the feature gene of NMF10 (Tissue-F), in MS and SLE,[82] and the reduction of effector Tregs and their decreased function in T1D,[83] are also consistent with our meta-analysis. In conclusion, by meta-analysis, in addition to quantitative changes, we identified qualitative changes depending on the disease, sex, and age at a resolution that is difficult to observe in existing methodologies.

Predictive potential of peripheral CD4$^+$ T profiles for autoimmune diseases

Given that each autoimmune disease disorder possessed a characteristic CD4$^+$ T cell profile, we hypothesized that disease status might be predicted solely from CD4$^+$ T profiles by utilizing machine learning techniques and our autoimmune-wide scRNA-seq dataset (Figure S12A). To confirm this, we created three models for the prediction of disease status. The first model took the frequency of each cell population in cluster L2, the second model took the cellular features of the NMF in subsets, and the last model took both as input parameters (Figure 3H). We note that, for NMFproj features, only Tnaive and Tcm (Th0) cell features, which were affected by various conditions as discussed previously (Figures 3G and S10A), were used to avoid over-fitting. First, binary classification models were constructed for SLE, COVID-19, and MS to distinguish between certain diseases and healthy individuals using logistic regression where equal numbers of diseased and healthy subjects were used for the training, and the models were evaluated using samples from independent projects from these used for the training (Figure 3I). SLE and COVID-19 yielded relatively good predictions from cell frequencies alone (area under the curve [AUC] = 0.84, 0.82 in SLE and COVID-19, respectively). NMF cell features alone also could predict SLE and COVID-19 well (AUC -= 0.94, 0.85 in SLE and COVID-19, respectively), and the perdition using both cell frequencies and NMFproj values further improved accuracy (AUC = 0.94, 0.91 in SLE and COVID-19 respectively). For MS, for which hematological biomarkers have not yet been well established, prediction from cell frequencies or from NMFproj values alone was not successful (AUC = 0.61, 0.47 in cell frequency and NMFproj, respectively), while both cell frequencies and NMFproj values resulted in better predictions (AUC = 0.75) than previous plasma based methods.[84] Next, we built multiclass classification models that more closely resemble real-world clinical practice. The multiclass classification was assumed to be a more difficult task due to the similarities among autoimmune diseases and the imbalance of training sample sizes. We trained a gradient boosting model with 5-fold cross-validation on data from 714 samples from 8 diseases or healthy for which at least 10 samples were available for training and then evaluated the model using the data from the independent dataset (Figures S12C and S12D). The model trained only by cell frequencies or by NMFproj values could predict COVID-19, SLE, HC, and MS (area under the precision-recall curve [PR-AUC] = 0.72, 0.68, 0.48, 0.12 in the cell frequency model and 0.91, 0.75, 0.47, 0.35 in the NMFproj model, for COVID-19, SLE, HC, and MS, respectively), and the model trained by both cell frequencies and NMFproj values in Tnaive and Tcm (Th0) was marked with superior accuracy (PR-AUC = 0.92, 0.69, 050, 0.33 for COVID-19, SLE, HC, and MS, respectively). The combined cell frequency and NMF model also achieved the highest

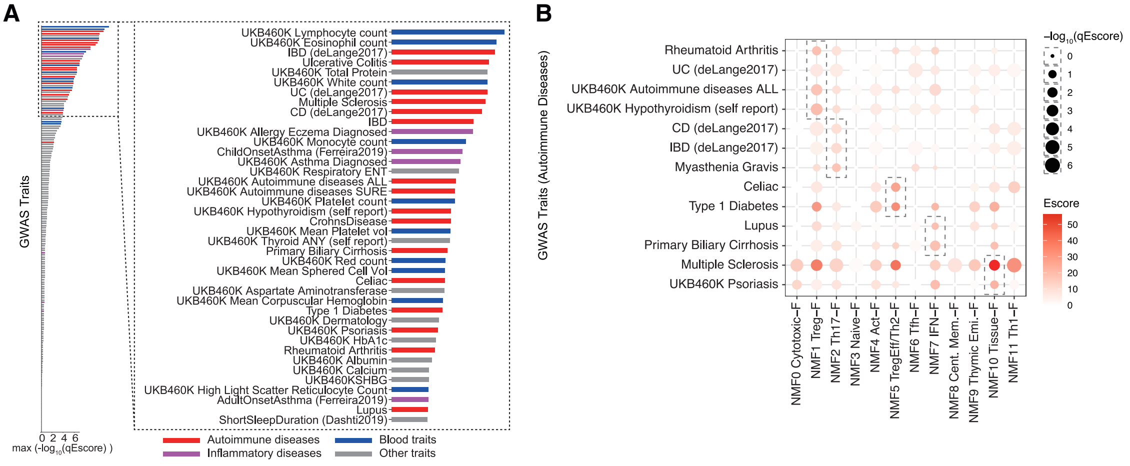

Figure 4. Partitioned heritability of autoimmune diseases by CD4⁺ T cell features

(A) Bar plot showing maximum $-\log_{10}$(q_ *E* score) among NMF gene features. Partitioned heritability of GWASs was measured using the sc-linker framework. Enrichment of each category is the following: autoimmune diseases, $p = 8.51 \times 10^{-12}$; inflammatory traits, $p = 0.131$; and blood cell count, $p = 7.59 \times 10^{-8}$ (two-sided Mann-Whitney U test).

(B) Dot plot showing enrichment of partitioned heritability of autoimmune diseases across NMF gene features. The dashed boxes indicate the factor with the highest *E* score for each disease. Duplicated traits were removed for the visualization. Full statistics are shown in Table S11.

accuracy (the cell frequency model: 0.413, the NMF model: 0.484, and the cell frequency + NMF model: 0.527). These results highlight that the disease-specific changes in $CD4^+$ T cells, both in terms of quantitative and qualitative alterations, contribute to the prediction of disease status.

Partitioned heritability of autoimmune diseases on $CD4^+$ T cell NMF features

We examined the association between $CD4^+$ T characteristics and genetic factors for each disease and trait. Studies using GWAS statistics have reported associations between autoimmune diseases and immune cells.[2,85] In particular, stratified linkage disequilibrium score regression (S-LDSC) has revealed the association between $CD4^+$ T cells and autoimmune diseases by stratifying the heritability of polygenic autoimmune diseases by genetic features.[86,87] Since we captured elaborate $CD4^+$ T cell features, we investigated which of these features was associated with each trait using the S-LDSC framework. In addition to the cell-type-specific genes of cluster L2, 12-dimensional features extracted by NMF were used as genetic features. The Roadmap enhancer-gene linking (Roadmap) and activity-by-contact strategies introduced in the sc-linker framework[88] were used for linking genes and SNPs. We note that as some gene programs were shared with other immune cells (Figure S6G), some associations below may potentially be influenced a broader range of cell types. Among 180 traits, autoimmune diseases showed significantly high enrichment in NMF features ($p = 8.51 \times 10^{-12}$), suggesting autoimmunity is closely associated with $CD4^+$ T cells (Figure 4A). Cross-sectional disease association revealed that many diseases, such as inflammatory bowel disease (IBD), RA, and MG, have an enrichment of heritability on NMF1 (Treg-F) (Figure 4B). By focusing on the most accumulated factors for each disease, we found that autoimmune diseases can be divided into several groups. For each disease, the most enriched gene features were observed as NMF1 (Treg-F): RA, UC (deLange), hypothyroidism; NMF2 (Th17-F): CD (deLange), IBD (deLange), MG; NMF5 (TregEff/Th2-F): celiac disease, T1D; NMF7 (IFN-F): SLE, primary biliary cirrhosis; NMF10 (Tissue-F): MS, psoriasis. In MS, accumulation was observed in various features, including NMF2 (Th17-F) and NMF11 (Th1-F). The heritability of each autoimmune disease was accumulated in several factors, suggesting that autoimmune diseases have multiple susceptibilities. In other traits, weak accumulation on NMF1 (Treg-F), NMF2 (Th17-F), and NMF11 (Th1-F) was common in COVID-19 in both severe symptoms and infection, while NMF7 (IFN-F) was infection specific (Figure S13A). Lymphocyte counts were also susceptible to NMF4 (Act-F) (Figure S13A). We also examined enrichment in cell-type-specific genes. Similar enrichment patterns, such as Treg Naive in most autoimmune diseases and Tnaive *MX1* in SLE, were observed, while caution should be taken as marker gene detection is not optimal for $CD4^+$ T cells as in the previous section (Figure S13B). Taken together, we comprehensively profiled heritability enrichment on $CD4^+$ T cell gene features across autoimmune diseases.

Partitioned heritability is associated with qualitative and/or quantitative changes in $CD4^+$ T in a disease-specific manner

Finally, we compared partitioned heritability and observed changes in $CD4^+$ T in terms of quantity (cell frequency) and quality (NMFproj) to assess the genetic effect on phenotypic changes (Figure 5A). We first investigated the correlation between enriched heritability and changes in cell frequency and NMF features for diseases that were enrolled in our analysis for both GWASs and meta-analysis. We found several patterns depending on the disease (Figure 5B). First, in MG and psoriasis, both changes in cell frequency and NMF feature correlated with

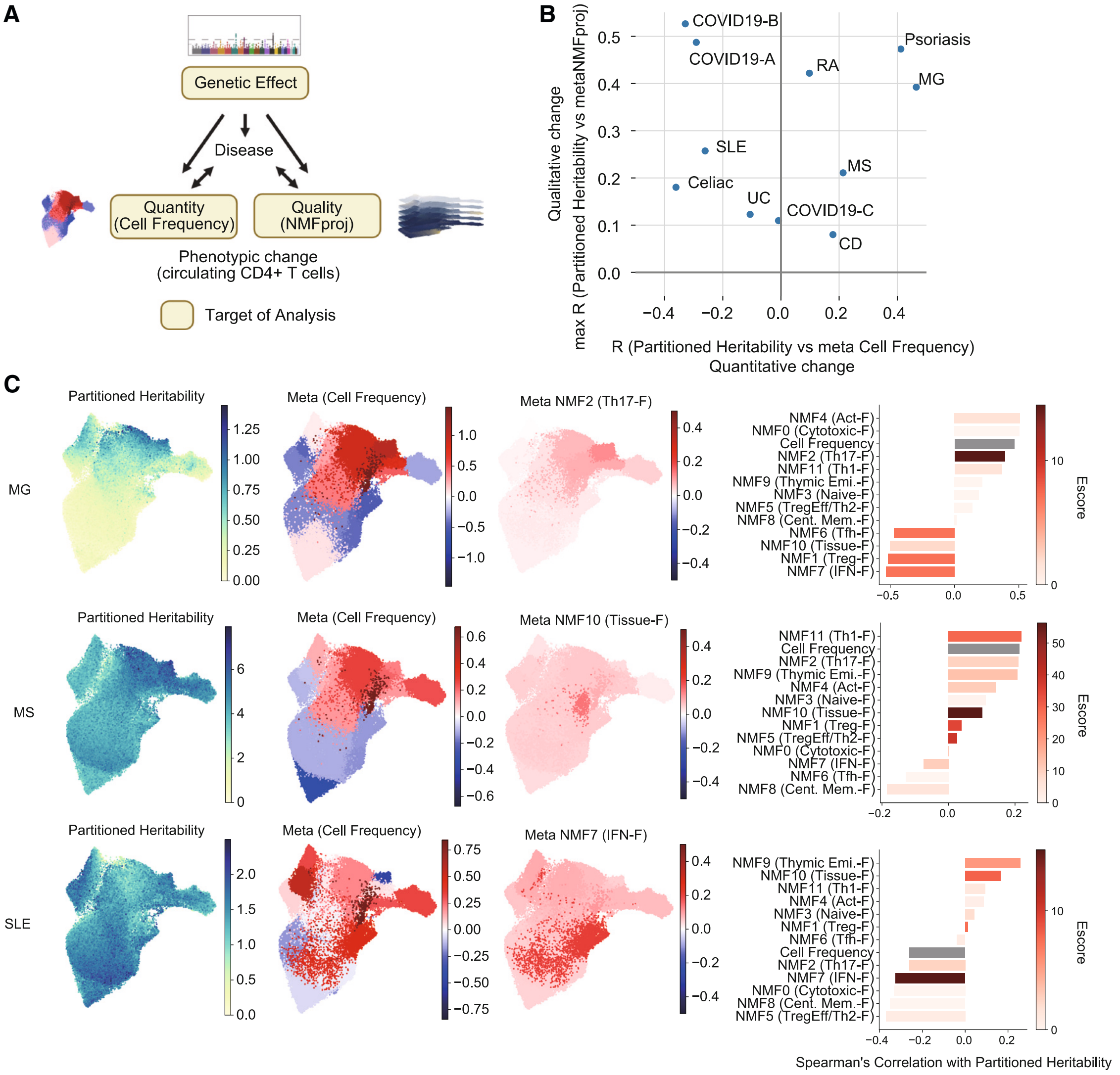

Figure 5. Relationship between genetic factors and phenotypic changes in CD4+ T cells

(A) Model of genetic effect on phenotypic changes in CD4+ T cells. CD4+ T cell changes are observed as qualitative (NMFproj cell features) and quantitative (cell-type frequencies) changes.

(B) Scatterplot showing the genetic effect on cell frequencies (x axis) and NMF features (y axis). Sc-linker weight per cell was calculated by dot products of sc-linker outcome (NMF) and NMF cell features. For cell frequencies and NMF cell features, coefficients of GLM output for each cluster L2 population were used. Spearman's correlation of sc-linker weight and cell frequency/NMF cell feature changes were calculated. For the correlation with sc-linker and NMF cell feature changes, we used the maximum R among NMF features for the visualization. COVID19-A, very severe respiratory symptom; COVID19-B, hospitalized; COVID19-C, SARS-CoV-2 infection.

(C) Individual sc-linker weights, cell frequency changes (coefficient for each cluster L2), and NMF cell feature changes in the factor with the highest magnitude (*E* score) (coefficient for each cluster L2) of MS, MG, and SLE were visualized on the UMAP embeddings (left panel). For the coefficient of the NMF cell feature changes, only one representative factor with the highest *E* score for each disease is shown. The bar plot of Spearman's correlation of cell frequency and NMFproj changes with partitioned heritability is shown in the right panel. The colors of the bars, except for cell frequency, indicate the *E* score calculated using sc-linker.

heritability accumulation. In severe COVID-19 (COVID19-A, COVID19-B) and RA, the correlation was mainly observed with changes in quality only. SLE, celiac disease, UC, and SARS-CoV-2 infection (COVID19-C) showed poor correlation with cell frequency.

Next, we examined MS, MG, and SLE, whose samples were collected in this study, in more detail (Figure 5C). In MG, NMF2 (Th17-F), which accumulated heritability most significantly, was increased in Tcm (Th17) and Tem (Th1/17) in correlation with genetic factor accumulation, and the cell frequency was also correlated with genetic factors (Figures 5C and S13). On the other hand, NMF1 (Treg-F), which showed the next highest accumulation of heritability, was negatively correlated with the genetic effect and lower in Treg cells (Figure S13), indicating that the dysfunction of Treg cells might be enhanced by the genetic effect. In MS, the highest heritability accumulation was observed

in NMF10 (Tissue-F). This factor was increased in all cell populations without cell specificity, resulting in a low correlation with the heritability. SLE susceptibility was most accumulated in NMF7 (IFN-F). In our meta-analysis, an enhancement of NMF7 (IFN-F) was observed in all cell populations, especially in Tnaive *MX1*. Overall, our study cataloged heritability enrichment and phenotypic changes across autoimmune diseases, enabling elucidation of the disease-specific effect of underlying genetic factors on $CD4^+$ T cell phenotypes.

DISCUSSION

The classifications and characterizations of $CD4^+$ T cells have been challenging, with cellular heterogeneity being a major obstacle.[4] In this study, by performing single-cell analysis on $CD4^+$ T cells from autoimmune and healthy subjects, we succeeded in mutually exclusive and collectively exhaustive subtype identifications of peripheral $CD4^+$ T cells. Moreover, in contrast to the conventional dualistic comparisons, such as Th1 vs. Th2 and Treg vs. Th17, the NMF-based decomposition revealed that $CD4^+$ T cells are formed by a combination of 12 features rather than simple contradistinction. While qualitative profiling by NMF was not suitable for numerical evaluation, it allowed for a more robust assessment of gradual cell populations. Moreover, our results can also be extrapolated for other single-cell and bulk RNA-seq studies by using a label transfer and the projection of NMF features.

These analytical frameworks also allowed us to perform autoimmune-wide single-cell meta-analyses and integration of $CD4^+$ T cell features with GWASs. As a result, we comprehensively cataloged $CD4^+$ T cell alterations in 20 diseases, providing a valuable resource for a broad range of disease research. The assessment of qualitative changes through NMFproj enabled us to explore biological insights, such as Treg functional abnormalities, that were previously unattainable using cytometry. Furthermore, the decomposition of gene programs using NMF was beneficial not only for T cell profiling but also for interpreting GWAS results. We found that genetic factors can have both disease-specific and cross-disease impacts on autoimmune conditions. The accumulation of heritability on Tregs across diseases and the disease specificity of other features may be potential clues for future therapeutic development.

By examining genetic factors, $CD4^+$ T cell changes, and TCR characteristics in a disease-specific manner, we gained valuable insights into various diseases. For example, MG is caused by autoantibodies against the neuromuscular junction, with germinal center responses involving Tfh cells and B cells within the thymus.[7,89] While Th17 function enhancement has been reported in MG,[73,90] we also observed a heritability enrichment in NMF2 (Th17-F), suggesting that tissue damage by Th17 cells may contribute to symptom completion and persistence. In MS, we observed an increase in NMF10 (Tissue-F) and heritability enrichment in addition to the previously known Th17 and Th1 increase and functional enhancement.[74,75,91] These results suggest that a strong tissue inflammatory response is involved in MS and emphasizes that the tissue-specific gene program centered on the AP-1 family may be a novel MS-specific therapeutic target.[92] In addition, in MS, while Treg Eff slightly increased, the quality of Treg cells in terms of transcriptome and TCR was low, indicating that the compensation of Tregs from Tconvs is an explanation for Treg dysfunction in MS.[93] In SLE, our analysis of heritability enrichment and qualitative alterations supports the traditional belief that type I IFN is central to the disease.[94] Type I IFN drives differentiation into Tregs and Th1 cells,[95,96] and our result suggests that the pleiotropic effect of type I IFN contributed to the complicated cell frequency changes observed in this study. Furthermore, TCR overlaps between Tcm (Tfh) and Treg Act were observed specifically in SLE, suggesting potential Treg-Tfh plasticity in SLE similar to reported Tfh-Treg plasticity under certain inflammatory conditions.[97,98] We identified distinct $CD4^+$ T cell responses between COVID-19 and influenza infection, with an increase in Tnaive cells in COVID-19 and Temra cells in flu. This divergence may reflect differences between pre-trained immunity to influenza and initial responses to SARS-CoV-2, as most COVID-19 samples were collected before the vaccine rollout. In addition, our meta-analysis revealed sex- and age-related $CD4^+$ T cell changes, with new observations such as increased Tnaive *MX1* and Tem (Tph) in females, potentially contributing to gender differences in autoimmune disease incidence. Thus, our study highlights the $CD4^+$ T cell features of each disease and condition, providing new insights for consideration.

In addition, this study created a comprehensive single-cell circulating $CD4^+$ T cell catalog across various diseases, providing the opportunity to tackle the challenging task of assessing whether disease prediction is feasible using $CD4^+$ T cell profiles. Our framework offers an approach to measuring $CD4^+$ T profiles from limited cell counts or sparse gene expression data through the pre-defined reference and NMFproj. However, the predictive accuracy of the machine learning model utilizing $CD4^+$ T profiles in this study was still limited, which could be attributed to factors such as limited sample sizes, sample imbalance, and variations between cohorts. Nevertheless, this underscores the potential for predicting disease status using these profiles. Moreover, the distinct variations in $CD4^+$ T profiles across diseases emphasize the necessity for individualized treatments tailored to specific conditions.

In summary, we constructed the frameworks for extracting the $CD4^+$ T cell programs, enabling a comprehensive interpretation of $CD4^+$ T cells. Moreover, the landscape of disease-specific $CD4^+$ T cell alterations and genetic effects provides biological insights for potential precision medicine.

Limitations of the study

There are several limitations worth noting. Firstly, since our meta-analysis contained data from not only whole PBMCs but also pre-enriched $CD4^+$ T cell samples, we could not consider the proportional alterations of $CD4^+$ T cells in overall PBMCs. Furthermore, our study focused on peripheral blood and did not evaluate tissue-specific alterations, such as barrier tissue-specific programs.[99] This might be the reason that we could not capture some known vital phenomena for $CD4^+$ T cells, such as anergy and exhaustion. For example, when we compared with *ab initio* NMF for TILs, while the factors defined in blood were largely preserved $CD4^+$ T cells in tissues, it is worth noting that certain factors, including those encompassing

CXCL13 and *IL21*, were defined only in TILs, emphasizing the importance of analyses using tissue samples in the future.

In addition, while our study provides insights into the relationship between gene programs in CD4$^+$ T cells and autoimmune diseases, further experimental validation such as interventions in genes and environments *in vitro* could offer more precise gene targeting and therapeutic applications in real-world contexts. Integrating these methodologies with our findings and using NMFproj for transcriptome evaluations might accelerate research for autoimmune diseases and cellular manipulations.

STAR★METHODS

Detailed methods are provided in the online version of this paper and include the following:

- KEY RESOURCES TABLE
- RESOURCE AVAILABILITY
  - Lead contact
  - Materials availability
  - Data and code availability
- EXPERIMENTAL MODEL AND STUDY PARTICIPANT DETAILS
  - Human samples
- METHOD DETAILS
  - Cell preparation and sequencing of scRNA-seq
  - Preprocess of scRNA-seq data
  - Integration with bulk RNA-seq dataset
  - Gene expression decomposition using NMF
  - TCR analysis
  - Heritability partitioning
  - Comparison of our ref-mapping classification performance with other classification tools
  - Meta-analysis of CD4$^+$ T cells from public datasets
  - Machine learning for the prediction of autoimmune states
- QUANTIFICATION AND STATISTICAL ANALYSIS

SUPPLEMENTAL INFORMATION

Supplemental information can be found online at https://doi.org/10.1016/j.xgen.2023.100473.

ACKNOWLEDGMENTS

This work was supported by Grant-in-Aid for Specially Promoted Research Grant 16H06295 from the Ministry of Education, Culture, Sports, Science and Technology of Japan and Leading Advanced Projects for Medical Innovation (LEAP) (no. 18 gm0010005h0001) from Japan's Agency for Medical Research and Development (AMED) to S.S. Y.Y. was supported by the Takeda Science Foundation. D.T. was supported by the Osaka University Medical Doctor Scientist Training Program. We acknowledge the NGS core facility of the Genome Information Research Center at the Research Institute for Microbial Diseases of Osaka University for its support in RNA sequencing. This work was partly achieved through the use of SQUID at the Cybermedia Center, Osaka University. Some illustrations were generated with BioRender.com. The authors wish to acknowledge Dr. Hirotaka Matsumoto, Nagasaki University, for his constructive feedback on NMFproj; Dr. Kazuyoshi Ishigaki (RIKEN) for technical advice for TiRP analysis; Nicole Carter and Sarah Schroeder (Parse Biosciences), Dr. Francesca Capon and Dr. Daniel McCluskey (King's College London), Dr. Karine Chemin (Karolinska Institutet), and Dr. Lennart M. Roesner (Hannover Medical School [MHH]), for sharing scRNA-seq data; and Dr. James Wing for critical comments on the manuscript.

AUTHOR CONTRIBUTIONS

Y.Y., D.T., R.M., N.O., and S.S. designed all experiments. Y.N. performed the experiments under the supervision of N.O.; Y.Y., D.T., and R.M. performed bioinformatics analysis; Y.Y., T.O., M.K., T.M., Y.K., A.K., and H.M. collected samples for analysis. M.W. and X.Z. provided scRNA-seq data. D.M. and D.O. performed library construction and sequencing. Y.T., N.M., and M.A. provided expert advice. Y.Y. prepared the figures. Y.Y., D.T., and R.M. drafted the manuscript. N.O. and S.S. supervised the study. All authors critically reviewed and edited the final version of the manuscript.

DECLARATION OF INTERESTS

The authors declare no competing interests.

DECLARATION OF GENERATIVE AI AND AI-ASSISTED TECHNOLOGIES IN THE WRITING PROCESS

During the preparation of this work the authors used ChatGPT4 in order to improve language and readability. After using this tool/service, the authors reviewed and edited the content as needed and take full responsibility for the content of the publication.

Received: May 9, 2023
Revised: September 7, 2023
Accepted: December 5, 2023
Published: January 3, 2024

REFERENCES

1. Wang, L., Wang, F.-S., and Gershwin, M.E. (2015). Human autoimmune diseases: a comprehensive update. J. Intern. Med. *278*, 369–395.
2. Farh, K.K.-H., Marson, A., Zhu, J., Kleinewietfeld, M., Housley, W.J., Beik, S., Shoresh, N., Whitton, H., Ryan, R.J.H., Shishkin, A.A., et al. (2015). Genetic and epigenetic fine mapping of causal autoimmune disease variants. Nature *518*, 337–343.
3. Ohkura, N., Yasumizu, Y., Kitagawa, Y., Tanaka, A., Nakamura, Y., Motooka, D., Nakamura, S., Okada, Y., and Sakaguchi, S. (2020). Regulatory T Cell-Specific Epigenomic Region Variants Are a Key Determinant of Susceptibility to Common Autoimmune Diseases. Immunity *52*, 1119–1132.e4.
4. Zhu, J., Yamane, H., and Paul, W.E. (2010). Differentiation of effector CD4 T cell populations (*). Annu. Rev. Immunol. *28*, 445–489.
5. Sakaguchi, S., Mikami, N., Wing, J.B., Tanaka, A., Ichiyama, K., and Ohkura, N. (2020). Regulatory T Cells and Human Disease. Annu. Rev. Immunol. *38*, 541–566.
6. Perez, R.K., Gordon, M.G., Subramaniam, M., Kim, M.C., Hartoularos, G.C., Targ, S., Sun, Y., Ogorodnikov, A., Bueno, R., Lu, A., et al. (2022). Single-cell RNA-seq reveals cell type–specific molecular and genetic associations to lupus. Science *376*, eabf1970.
7. Yasumizu, Y., Ohkura, N., Murata, H., Kinoshita, M., Funaki, S., Nojima, S., Kido, K., Kohara, M., Motooka, D., Okuzaki, D., et al. (2022). Myasthenia gravis-specific aberrant neuromuscular gene expression by medullary thymic epithelial cells in thymoma. Nat. Commun. *13*, 4230.
8. Schafflick, D., Xu, C.A., Hartlehnert, M., Cole, M., Schulte-Mecklenbeck, A., Lautwein, T., Wolbert, J., Heming, M., Meuth, S.G., Kuhlmann, T., et al. (2020). Integrated single cell analysis of blood and cerebrospinal fluid leukocytes in multiple sclerosis. Nat. Commun. *11*, 247.
9. Zhang, F., Wei, K., Slowikowski, K., Fonseka, C.Y., Rao, D.A., Kelly, S., Goodman, S.M., Tabechian, D., Hughes, L.B., Salomon-Escoto, K., et al. (2019). Defining inflammatory cell states in rheumatoid arthritis joint

synovial tissues by integrating single-cell transcriptomics and mass cytometry. Nat. Immunol. *20*, 928–942.

10. Martin, J.C., Chang, C., Boschetti, G., Ungaro, R., Giri, M., Grout, J.A., Gettler, K., Chuang, L.S., Nayar, S., Greenstein, A.J., et al. (2019). Single-Cell Analysis of Crohn's Disease Lesions Identifies a Pathogenic Cellular Module Associated with Resistance to Anti-TNF Therapy. Cell *178*, 1493–1508.e20.
11. Argyriou, A., Wadsworth, M.H., 2nd, Lendvai, A., Christensen, S.M., Hensvold, A.H., Gerstner, C., van Vollenhoven, A., Kravarik, K., Winkler, A., Malmström, V., and Chemin, K. (2022). Single cell sequencing identifies clonally expanded synovial CD4+ TPH cells expressing GPR56 in rheumatoid arthritis. Nat. Commun. *13*, 4046.
12. Stuart, T., Butler, A., Hoffman, P., Hafemeister, C., Papalexi, E., Mauck, W.M., Hao, Y., Stoeckius, M., Smibert, P., and Satija, R. (2019). Comprehensive Integration of Single-Cell Data. Cell *177*, 1888–1902.e21.
13. Korsunsky, I., Millard, N., Fan, J., Slowikowski, K., Zhang, F., Wei, K., Baglaenko, Y., Brenner, M., Loh, P.R., and Raychaudhuri, S. (2019). Fast, sensitive and accurate integration of single-cell data with Harmony. Nat. Methods *16*, 1289–1296.
14. Kang, J.B., Nathan, A., Weinand, K., Zhang, F., Millard, N., Rumker, L., Moody, D.B., Korsunsky, I., and Raychaudhuri, S. (2021). Efficient and precise single-cell reference atlas mapping with Symphony. Nat. Commun. *12*, 5890.
15. Zheng, L., Qin, S., Si, W., Wang, A., Xing, B., Gao, R., Ren, X., Wang, L., Wu, X., Zhang, J., et al. (2021). Pan-cancer single-cell landscape of tumor-infiltrating T cells. Science *374*, abe6474.
16. Andreatta, M., Corria-Osorio, J., Müller, S., Cubas, R., Coukos, G., and Carmona, S.J. (2021). Interpretation of T cell states from single-cell transcriptomics data using reference atlases. Nat. Commun. *12*, 2965.
17. Andreatta, M., Tjitropranoto, A., Sherman, Z., Kelly, M.C., Ciucci, T., and Carmona, S.J. (2022). A CD4+ T cell reference map delineates subtype-specific adaptation during acute and chronic viral infections. Elife *11*, e76339.
18. Nathan, A., Asgari, S., Ishigaki, K., Valencia, C., Amariuta, T., Luo, Y., Beynor, J.I., Baglaenko, Y., Suliman, S., Price, A.L., et al. (2022). Single-cell eQTL models reveal dynamic T cell state dependence of disease loci. Nature *606*, 120–128.
19. Morabito, S., Miyoshi, E., Michael, N., Shahin, S., Martini, A.C., Head, E., Silva, J., Leavy, K., Perez-Rosendahl, M., and Swarup, V. (2021). Single-nucleus chromatin accessibility and transcriptomic characterization of Alzheimer's disease. Nat. Genet. *53*, 1143–1155.
20. Stein-O'Brien, G.L., Arora, R., Culhane, A.C., Favorov, A.V., Garmire, L.X., Greene, C.S., Goff, L.A., Li, Y., Ngom, A., Ochs, M.F., et al. (2018). Enter the Matrix: Factorization Uncovers Knowledge from Omics. Trends Genet. *34*, 790–805.
21. Pelka, K., Hofree, M., Chen, J.H., Sarkizova, S., Pirl, J.D., Jorgji, V., Bejnood, A., Dionne, D., Ge, W.H., Xu, K.H., et al. (2021). Spatially organized multicellular immune hubs in human colorectal cancer. Cell *184*, 4734–4752.e20.
22. Kotliar, D., Veres, A., Nagy, M.A., Tabrizi, S., Hodis, E., Melton, D.A., and Sabeti, P.C. (2019). Identifying gene expression programs of cell-type identity and cellular activity with single-cell RNA-Seq. Elife *8*, e43803.
23. Schmiedel, B.J., Singh, D., Madrigal, A., Valdovino-Gonzalez, A.G., White, B.M., Zapardiel-Gonzalo, J., Ha, B., Altay, G., Greenbaum, J.A., McVicker, G., et al. (2018). Impact of Genetic Polymorphisms on Human Immune Cell Gene Expression. Cell *175*, 1701–1715.e16.
24. Rao, D.A., Gurish, M.F., Marshall, J.L., Slowikowski, K., Fonseka, C.Y., Liu, Y., Donlin, L.T., Henderson, L.A., Wei, K., Mizoguchi, F., et al. (2017). Pathologically expanded peripheral T helper cell subset drives B cells in rheumatoid arthritis. Nature *542*, 110–114.
25. Yoshitomi, H., and Ueno, H. (2021). Shared and distinct roles of T peripheral helper and T follicular helper cells in human diseases. Cell. Mol. Immunol. *18*, 523–527.
26. Asashima, H., Mohanty, S., Comi, M., Ruff, W.E., Hoehn, K.B., Wong, P., Klein, J., Lucas, C., Cohen, I., Coffey, S., et al. (2023). PD-1highCXCR5-CD4+ peripheral helper T cells promote CXCR3+ plasmablasts in human acute viral infection. Cell Rep. *42*, 111895.
27. Ohkura, N., Hamaguchi, M., Morikawa, H., Sugimura, K., Tanaka, A., Ito, Y., Osaki, M., Tanaka, Y., Yamashita, R., Nakano, N., et al. (2012). T Cell Receptor Stimulation-Induced Epigenetic Changes and Foxp3 Expression Are Independent and Complementary Events Required for Treg Cell Development. Immunity *37*, 785–799.
28. Lagattuta, K.A., Kang, J.B., Nathan, A., Pauken, K.E., Jonsson, A.H., Rao, D.A., Sharpe, A.H., Ishigaki, K., and Raychaudhuri, S. (2022). Repertoire analyses reveal T cell antigen receptor sequence features that influence T cell fate. Nat. Immunol. *23*, 446–457.
29. Rosenblum, M.D., Way, S.S., and Abbas, A.K. (2016). Regulatory T cell memory. Nat. Rev. Immunol. *16*, 90–101.
30. Delacher, M., Imbusch, C.D., Hotz-Wagenblatt, A., Mallm, J.-P., Bauer, K., Simon, M., Riegel, D., Rendeiro, A.F., Bittner, S., Sanderink, L., et al. (2020). Precursors for nonlymphoid-tissue Treg cells reside in secondary lymphoid organs and are programmed by the transcription factor BATF. Immunity *52*, 295–312.e11.
31. Wing, J.B., Kitagawa, Y., Locci, M., Hume, H., Tay, C., Morita, T., Kidani, Y., Matsuda, K., Inoue, T., Kurosaki, T., et al. (2017). A distinct subpopulation of $CD25^{-}$ T-follicular regulatory cells localizes in the germinal centers. Proc. Natl. Acad. Sci. USA *114*, E6400–E6409.
32. Gate, D., Tapp, E., Leventhal, O., Shahid, M., Nonninger, T.J., Yang, A.C., Strempfl, K., Unger, M.S., Fehlmann, T., Oh, H., et al. (2021). CD4 + T cells contribute to neurodegeneration in Lewy body dementia. Science *374*, 868–874.
33. Boyman, O., Purton, J.F., Surh, C.D., and Sprent, J. (2007). Cytokines and T-cell homeostasis. Curr. Opin. Immunol. *19*, 320–326.
34. Miyara, M., Yoshioka, Y., Kitoh, A., Shima, T., Wing, K., Niwa, A., Parizot, C., Taflin, C., Heike, T., Valeyre, D., et al. (2009). Functional Delineation and Differentiation Dynamics of Human CD4+ T Cells Expressing the FoxP3 Transcription Factor. Immunity *30*, 899–911.
35. Mikami, N., Kawakami, R., Chen, K.Y., Sugimoto, A., Ohkura, N., and Sakaguchi, S. (2020). Epigenetic conversion of conventional T cells into regulatory T cells by CD28 signal deprivation. Proc. Natl. Acad. Sci. USA *117*, 12258–12268.
36. Domínguez Conde, C., Xu, C., Jarvis, L.B., Rainbow, D.B., Wells, S.B., Gomes, T., Howlett, S.K., Suchanek, O., Polanski, K., King, H.W., et al. (2022). Cross-tissue immune cell analysis reveals tissue-specific features in humans. Science *376*, eabl5197.
37. Elyahu, Y., Hekselman, I., Eizenberg-Magar, I., Berner, O., Strominger, I., Schiller, M., Mittal, K., Nemirovsky, A., Eremenko, E., Vital, A., et al. (2019). Aging promotes reorganization of the CD4 T cell landscape toward extreme regulatory and effector phenotypes. Sci. Adv. *5*, eaaw8330.
38. Valencia, X., Yarboro, C., Illei, G., and Lipsky, P.E. (2007). Deficient CD4+CD25high T regulatory cell function in patients with active systemic lupus erythematosus. J. Immunol. *178*, 2579–2588.
39. Ota, M., Nagafuchi, Y., Hatano, H., Ishigaki, K., Terao, C., Takeshima, Y., Yanaoka, H., Kobayashi, S., Okubo, M., Shirai, H., et al. (2021). Dynamic landscape of immune cell-specific gene regulation in immune-mediated diseases. Cell *184*, 3006–3021.e17.
40. Nehar-Belaid, D., Hong, S., Marches, R., Chen, G., Bolisetty, M., Baisch, J., Walters, L., Punaro, M., Rossi, R.J., Chung, C.H., et al. (2020). Mapping systemic lupus erythematosus heterogeneity at the single-cell level. Nat. Immunol. *21*, 1094–1106.
41. Garman, L., Pelikan, R.C., Rasmussen, A., Lareau, C.A., Savoy, K.A., Deshmukh, U.S., Bagavant, H., Levin, A.M., Daouk, S., Drake, W.P., and Montgomery, C.G. (2020). Single Cell Transcriptomics Implicate Novel Monocyte and T Cell Immune Dysregulation in Sarcoidosis. Front. Immunol. *11*, 567342.

42. Stephenson, E., Reynolds, G., Botting, R.A., Calero-Nieto, F.J., Morgan, M.D., Tuong, Z.K., Bach, K., Sungnak, W., Worlock, K.B., Yoshida, M., et al. (2021). Single-cell multi-omics analysis of the immune response in COVID-19. Nat. Med. *27*, 904–916.

43. Wang, Z., Xie, L., Ding, G., Song, S., Chen, L., Li, G., Xia, M., Han, D., Zheng, Y., Liu, J., et al. (2021). Single-cell RNA sequencing of peripheral blood mononuclear cells from acute Kawasaki disease patients. Nat. Commun. *12*, 5444.

44. Lefferts, A.R., Regner, E.H., Stahly, A., O'Rourke, B., Gerich, M.E., Fennimore, B.P., Scott, F.I., Freeman, A.E., Jones, K., and Kuhn, K.A. (2021). Circulating mature granzyme B+ T cells distinguish Crohn's disease-associated axial spondyloarthritis from axial spondyloarthritis and Crohn's disease. Arthritis Res. Ther. *23*, 147.

45. Liu, J., Kumar, S., Hong, J., Huang, Z.-M., Paez, D., Castillo, M., Calvo, M., Chang, H.-W., Cummins, D.D., Chung, M., et al. (2022). Combined Single Cell Transcriptome and Surface Epitope Profiling Identifies Potential Biomarkers of Psoriatic Arthritis and Facilitates Diagnosis via Machine Learning. Front. Immunol. *13*, 835760.

46. Ramírez-Sánchez, A.D., Chu, X., Modderman, R., Kooy-Winkelaar, Y., Koletzko, S., Korponay-Szabó, I.R., Troncone, R., Wijmenga, C., Mearin, L., Withoff, S., et al. (2022). Single-Cell RNA Sequencing of Peripheral Blood Mononuclear Cells From Pediatric Coeliac Disease Patients Suggests Potential Pre-Seroconversion Markers. Front. Immunol. *13*, 843086.

47. Paley, M.A., Baker, B.J., Dunham, S.R., Linskey, N., Cantoni, C., Lee, K., Hassman, L.M., Laurent, J., Roberson, E.D.O., Clifford, D.B., and Yokoyama, W.M. (2022). The CSF in neurosarcoidosis contains consistent clonal expansion of CD8 T cells, but not CD4 T cells. J. Neuroimmunol. *367*, 577860.

48. Kaufmann, M., Evans, H., Schaupp, A.-L., Engler, J.B., Kaur, G., Willing, A., Kursawe, N., Schubert, C., Attfield, K.E., Fugger, L., and Friese, M.A. (2021). Identifying CNS-colonizing T cells as potential therapeutic targets to prevent progression of multiple sclerosis. Med *2*, 296–312.e8.

49. Cai, Y., Dai, Y., Wang, Y., Yang, Q., Guo, J., Wei, C., Chen, W., Huang, H., Zhu, J., Zhang, C., et al. (2020). Single-cell transcriptomics of blood reveals a natural killer cell subset depletion in tuberculosis. EBioMedicine *53*, 102686.

50. Wu, X., Liu, Y., Jin, S., Wang, M., Jiao, Y., Yang, B., Lu, X., Ji, X., Fei, Y., Yang, H., et al. (2021). Single-cell sequencing of immune cells from anticitrullinated peptide antibody positive and negative rheumatoid arthritis. Nat. Commun. *12*, 4977.

51. Hong, X., Meng, S., Tang, D., Wang, T., Ding, L., Yu, H., Li, H., Liu, D., Dai, Y., and Yang, M. (2020). Single-Cell RNA Sequencing Reveals the Expansion of Cytotoxic CD4+ T Lymphocytes and a Landscape of Immune Cells in Primary Sjögren's Syndrome. Front. Immunol. *11*, 594658.

52. Wilk, A.J., Rustagi, A., Zhao, N.Q., Roque, J., Martínez-Colón, G.J., McKechnie, J.L., Ivison, G.T., Ranganath, T., Vergara, R., Hollis, T., et al. (2020). A single-cell atlas of the peripheral immune response in patients with severe COVID-19. Nat. Med. *26*, 1070–1076.

53. McCluskey, D., Benzian-Olsson, N., Mahil, S.K., Hassi, N.K., Wohnhaas, C.T., APRICOT and PLUM study team; Burden, A.D., Griffiths, C.E.M., Ingram, J.R., Levell, N.J., et al. (2022). Single-cell analysis implicates T_H17-to-T_H2 cell plasticity in the pathogenesis of palmoplantar pustulosis. J. Allergy Clin. Immunol. *150*, 882–893.

54. Ren, X., Wen, W., Fan, X., Hou, W., Su, B., Cai, P., Li, J., Liu, Y., Tang, F., Zhang, F., et al. (2021). COVID-19 immune features revealed by a large-scale single-cell transcriptome atlas. Cell *184*, 1895–1913.e19.

55. Ramesh, A., Schubert, R.D., Greenfield, A.L., Dandekar, R., Loudermilk, R., Sabatino, J.J., Koelzer, M.T., Tran, E.B., Koshal, K., Kim, K., et al. (2020). A pathogenic and clonally expanded B cell transcriptome in active multiple sclerosis. Proc. Natl. Acad. Sci. USA *117*, 22932–22943.

56. Wang, P., Yao, L., Luo, M., Zhou, W., Jin, X., Xu, Z., Yan, S., Li, Y., Xu, C., Cheng, R., et al. (2021). Single-cell transcriptome and TCR profiling reveal activated and expanded T cell populations in Parkinson's disease. Cell Discov. *7*, 52.

57. Xu, H., and Jia, J. (2021). Single-Cell RNA Sequencing of Peripheral Blood Reveals Immune Cell Signatures in Alzheimer's Disease. Front. Immunol. *12*, 645666.

58. Zheng, W., Wang, X., Liu, J., Yu, X., Li, L., Wang, H., Yu, J., Pei, X., Li, C., Wang, Z., et al. (2022). Single-cell analyses highlight the proinflammatory contribution of C1q-high monocytes to Behçet's disease. Proc. Natl. Acad. Sci. USA *119*, e2204289119.

59. Boland, B.S., He, Z., Tsai, M.S., Olvera, J.G., Omilusik, K.D., Duong, H.G., Kim, E.S., Limary, A.E., Jin, W., Milner, J.J., et al. (2020). Heterogeneity and clonal relationships of adaptive immune cells in ulcerative colitis revealed by single-cell analyses. Sci. Immunol. *5*, eabb4432.

60. Tran, V., Papalexi, E., Schroeder, S., Kim, G., Sapre, A., Pangallo, J., Sova, A., Matulich, P., Kenyon, L., Sayar, Z., et al. (2022). High sensitivity single cell RNA sequencing with split pool barcoding. Preprint at bioRxiv.

61. Lee, J.S., Park, S., Jeong, H.W., Ahn, J.Y., Choi, S.J., Lee, H., Choi, B., Nam, S.K., Sa, M., Kwon, J.S., et al. (2020). Immunophenotyping of covid-19 and influenza highlights the role of type i interferons in development of severe covid-19. Sci. Immunol. *5*. eabd1554–14.

62. Hao, Y., Hao, S., Andersen-Nissen, E., Mauck, W.M., Zheng, S., Butler, A., Lee, M.J., Wilk, A.J., Darby, C., Zager, M., et al. (2021). Integrated analysis of multimodal single-cell data. Cell *184*, 3573–3587.e29.

63. Schmidt, D., Goronzy, J.J., and Weyand, C.M. (1996). CD4+ CD7- CD28- T cells are expanded in rheumatoid arthritis and are characterized by autoreactivity. J. Clin. Invest. *97*, 2027–2037.

64. Fasth, A.E.R., Cao, D., van Vollenhoven, R., Trollmo, C., and Malmström, V. (2004). CD28nullCD4+ T cells–characterization of an effector memory T-cell population in patients with rheumatoid arthritis. Scand. J. Immunol. *60*, 199–208.

65. Raveney, B.J.E., Sato, W., Takewaki, D., Zhang, C., Kanazawa, T., Lin, Y., Okamoto, T., Araki, M., Kimura, Y., Sato, N., et al. (2021). Involvement of cytotoxic Eomes-expressing $CD4^+$ T cells in secondary progressive multiple sclerosis. Proc. Natl. Acad. Sci. USA *118*, e2021818118.

66. Funderburg, N.T., Stubblefield Park, S.R., Sung, H.C., Hardy, G., Clagett, B., Ignatz-Hoover, J., Harding, C.V., Fu, P., Katz, J.A., Lederman, M.M., and Levine, A.D. (2013). Circulating CD4(+) and CD8(+) T cells are activated in inflammatory bowel disease and are associated with plasma markers of inflammation. Immunology *140*, 87–97.

67. Xie, Z., Huang, Y., Li, X., Lun, Y., Li, X., He, Y., Wu, S., Wang, S., Sun, J., and Zhang, J. (2022). Atlas of circulating immune cells in Kawasaki disease. Int. Immunopharm. *102*, 108396.

68. Knoop, J., Gavrisan, A., Kuehn, D., Reinhardt, J., Heinrich, M., Hippich, M., Eugster, A., Ockert, C., Ziegler, A.-G., and Bonifacio, E. (2018). GM-CSF producing autoreactive CD4+ T cells in type 1 diabetes. Clin. Immunol. *188*, 23–30.

69. Zhang, Q., Bastard, P., Liu, Z., Le Pen, J., Moncada-Velez, M., Chen, J., Ogishi, M., Sabli, I.K.D., Hodeib, S., Korol, C., et al. (2020). Inborn errors of type I IFN immunity in patients with life-threatening COVID-19. Science *370*, eabd4570.

70. Banchereau, J., and Pascual, V. (2006). Type I interferon in systemic lupus erythematosus and other autoimmune diseases. Immunity *25*, 383–392.

71. Yao, Y., Liu, Z., Jallal, B., Shen, N., and Rönnblom, L. (2013). Type I interferons in Sjögren's syndrome. Autoimmun. Rev. *12*, 558–566.

72. Apaolaza, P.S., Balcacean, D., Zapardiel-Gonzalo, J., Nelson, G., Lenchik, N., Akhbari, P., Gerling, I., Richardson, S.J., and Rodriguez-Calvo, T.; nPOD-Virus Group (2021). Islet expression of type I interferon response sensors is associated with immune infiltration and viral infection in type 1 diabetes. Sci. Adv. *7*, eabd6527.

CellPress
OPEN ACCESS

73. Wang, Z., Wang, W., Chen, Y., and Wei, D. (2012). T helper type 17 cells expand in patients with myasthenia-associated thymoma. Scand. J. Immunol. *76*, 54–61.

74. Jones, A.P., Trend, S., Byrne, S.N., Fabis-Pedrini, M.J., Geldenhuys, S., Nolan, D., Booth, D.R., Carroll, W.M., Lucas, R.M., Kermode, A.G., and Hart, P.H. (2017). Altered regulatory T-cell fractions and Helios expression in clinically isolated syndrome: clues to the development of multiple sclerosis. Clin. Transl. Immunology *6*, e143.

75. Kleinewietfeld, M., and Hafler, D.A. (2013). The plasticity of human Treg and Th17 cells and its role in autoimmunity. Semin. Immunol. *25*, 305–312.

76. Kanai, Y., Satoh, T., Igawa, K., and Yokozeki, H. (2012). Impaired expression of Tim-3 on Th17 and Th1 cells in psoriasis. Acta Derm. Venereol. *92*, 367–371.

77. Christodoulou, M.I., Kapsogeorgou, E.K., Moutsopoulos, N.M., and Moutsopoulos, H.M. (2008). Foxp3+ T-regulatory cells in sjögren's syndrome. Am. J. Pathol. *173*, 1389–1396.

78. Mogilenko, D.A., Shchukina, I., and Artyomov, M.N. (2022). Immune ageing at single-cell resolution. Nat. Rev. Immunol. *22*, 484–498.

79. Klein, S.L., and Flanagan, K.L. (2016). Sex differences in immune responses. Nat. Rev. Immunol. *16*, 626–638.

80. Pido-Lopez, J., Imami, N., and Aspinall, R. (2001). Both age and gender affect thymic output: more recent thymic migrants in females than males as they age. Clin. Exp. Immunol. *125*, 409–413.

81. Abdullah, M., Chai, P.-S., Chong, M.-Y., Tohit, E.R.M., Ramasamy, R., Pei, C.P., and Vidyadaran, S. (2012). Gender effect on in vitro lymphocyte subset levels of healthy individuals. Cell. Immunol. *272*, 214–219.

82. González-Amaro, R., Cortés, J.R., Sánchez-Madrid, F., and Martín, P. (2013). Is CD69 an effective brake to control inflammatory diseases? Trends Mol. Med. *19*, 625–632.

83. Okubo, Y., Torrey, H., Butterworth, J., Zheng, H., and Faustman, D.L. (2016). Treg activation defect in type 1 diabetes: correction with TNFR2 agonism. Clin. Transl. Immunology *5*, e56.

84. Huang, J., Khademi, M., Fugger, L., Lindhe, Ö., Novakova, L., Axelsson, M., Malmeström, C., Constantinescu, C., Lycke, J., Piehl, F., et al. (2020). Inflammation-related plasma and CSF biomarkers for multiple sclerosis. Proc. Natl. Acad. Sci. USA *117*, 12952–12960.

85. Zhang, M.J., Hou, K., Dey, K.K., Sakaue, S., Jagadeesh, K.A., Weinand, K., Taychameekiatchai, A., Rao, P., Pisco, A.O., Zou, J., et al. (2022). Polygenic enrichment distinguishes disease associations of individual cells in single-cell RNA-seq data. Nat. Genet. *1–9*.

86. Finucane, H.K., Bulik-Sullivan, B., Gusev, A., Trynka, G., Reshef, Y., Loh, P.R., Anttila, V., Xu, H., Zang, C., Farh, K., et al. (2015). Partitioning heritability by functional annotation using genome-wide association summary statistics. Nat. Genet. *47*, 1228–1235.

87. Kanai, M., Akiyama, M., Takahashi, A., Matoba, N., Momozawa, Y., Ikeda, M., Iwata, N., Ikegawa, S., Hirata, M., Matsuda, K., et al. (2018). Genetic analysis of quantitative traits in the Japanese population links cell types to complex human diseases. Nat. Genet. *50*, 390–400.

88. Jagadeesh, K.A., Dey, K.K., Montoro, D.T., Mohan, R., Gazal, S., Engreitz, J.M., Xavier, R.J., Price, A.L., and Regev, A. (2022). Identifying disease-critical cell types and cellular processes by integrating single-cell RNA-sequencing and human genetics. Nat. Genet. *54*, 1479–1492.

89. Song, Y., Zhou, L., Miao, F., Chen, G., Zhu, Y., Gao, X., Wang, Y., Pang, L., Zhao, C., Sun, X., and Chen, Z. (2016). Increased frequency of thymic T follicular helper cells in myasthenia gravis patients with thymoma. J. Thorac. Dis. *8*, 314–322.

90. Villegas, J.A., Bayer, A.C., Ider, K., Bismuth, J., Truffault, F., Roussin, R., Santelmo, N., Le Panse, R., Berrih-Aknin, S., and Dragin, N. (2019). Il-23/Th17 cell pathway: A promising target to alleviate thymic inflammation maintenance in myasthenia gravis. J. Autoimmun. *98*, 59–73.

91. Nylander, A., and Hafler, D.A. (2012). Multiple sclerosis. J. Clin. Invest. *122*, 1180–1188.

92. Hasan, Z., Koizumi, S.-I., Sasaki, D., Yamada, H., Arakaki, N., Fujihara, Y., Okitsu, S., Shirahata, H., and Ishikawa, H. (2017). JunB is essential for IL-23-dependent pathogenicity of Th17 cells. Nat. Commun. *8*, 15628.

93. Viglietta, V., Baecher-Allan, C., Weiner, H.L., and Hafler, D.A. (2004). Loss of functional suppression by CD4+CD25+ regulatory T cells in patients with multiple sclerosis. J. Exp. Med. *199*, 971–979.

94. Psarras, A., Wittmann, M., and Vital, E.M. (2022). Emerging concepts of type I interferons in SLE pathogenesis and therapy. Nat. Rev. Rheumatol. *18*, 575–590.

95. Fueyo-González, F., McGinty, M., Ningoo, M., Anderson, L., Cantarelli, C., Angeletti, A., Demir, M., Llaudó, I., Purroy, C., Marjanovic, N., et al. (2022). Interferon-β acts directly on T cells to prolong allograft survival by enhancing regulatory T cell induction through Foxp3 acetylation. Immunity *55*, 459–474.e7.

96. Brinkmann, V., Geiger, T., Alkan, S., and Heusser, C.H. (1993). Interferon alpha increases the frequency of interferon gamma-producing human CD4+ T cells. J. Exp. Med. *178*, 1655–1663.

97. Le Coz, C., Oldridge, D.A., Herati, R.S., De Luna, N., Garifallou, J., Cruz Cabrera, E., Belman, J.P., Pueschl, D., Silva, L.V., Knox, A.V.C., et al. (2023). Human T follicular helper clones seed the germinal center-resident regulatory pool. Sci. Immunol. *8*, eade8162.

98. Jacobsen, J.T., Hu, W., R Castro, T.B., Solem, S., Galante, A., Lin, Z., Allon, S.J., Mesin, L., Bilate, A.M., Schiepers, A., et al. (2021). Expression of Foxp3 by T follicular helper cells in end-stage germinal centers. Science *373*, eabe5146.

99. Poon, M.M.L., Caron, D.P., Wang, Z., Wells, S.B., Chen, D., Meng, W., Szabo, P.A., Lam, N., Kubota, M., Matsumoto, R., et al. (2023). Tissue adaptation and clonal segregation of human memory T cells in barrier sites. Nat. Immunol. *24*, 309–319.

100. Wolf, F.A., Angerer, P., and Theis, F.J. (2018). SCANPY: large-scale single-cell gene expression data analysis. Genome Biol. *19*, 15.

101. Pedregosa, F., Varoquaux, G., Gramfort, A., Michel, V., Thirion, B., Grisel, O., Blondel, M., Müller, A., Nothman, J., Louppe, G., et al. (2012). Scikit-learn: Machine Learning in Python. Preprint at arXiv.

102. Hiraoka, Y., Yamada, K., Kawasaki, Y., Hirose, H., Matsumoto, Y., Ishikawa, K., and Yasumizu, Y. (2019). Ikra : RNAseq Pipeline Centered on Salmon (Zenodo).

103. Yu, G., Wang, L.-G., Han, Y., and He, Q.-Y. (2012). clusterProfiler: an R Package for Comparing Biological Themes Among Gene Clusters. OMICS *16*, 284–287.

104. Yu, G., and He, Q.-Y. (2016). ReactomePA: an R/Bioconductor package for reactome pathway analysis and visualization. Mol. Biosyst. *12*, 477–479.

105. Sturm, G., Szabo, T., Fotakis, G., Haider, M., Rieder, D., Trajanoski, Z., and Finotello, F. (2020). Scirpy: a Scanpy extension for analyzing single-cell T-cell receptor-sequencing data. Bioinformatics *36*, 4817–4818.

106. Bulik-Sullivan, B.K., Loh, P.R., Finucane, H.K., Ripke, S., Yang, J., Schizophrenia Working Group of the Psychiatric Genomics Consortium; Patterson, N., Daly, M.J., Price, A.L., Neale, B.M., et al. (2015). LD score regression distinguishes confounding from polygenicity in genome-wide association studies. Nat. Genet. *47*, 291–295.

107. Lemaitre, G., Nogueira, F., and Aridas, C.K. (2016). Imbalanced-learn: A python toolbox to tackle the curse of imbalanced datasets in machine learning. Preprint at arXiv.

108. Ke, G., Meng, Q., Finley, T., Wang, T., Chen, W., Ma, W., Ye, Q., Liu, T.-Y., Research, M., University, P., et al. (2017). LightGBM: A Highly Efficient Gradient Boosting Decision Tree. https://papers.nips.cc/paper/2017/file/6449f44a102fde848669bdd9eb6b76fa-Paper.pdf.

109. Seabold, S., and Perktold, J. (2010). Statsmodels: Econometric and statistical modeling with python. In Proceedings of the 9th Python in Science Conference (SciPy)).

110. Krueger, F., James, F., Ewels, P., Afyounian, E., and Schuster-Boeckler, B. (2021). FelixKrueger/TrimGalore: v0.6.7 (Zenodo).

111. Patro, R., Duggal, G., Love, M.I., Irizarry, R.A., and Kingsford, C. (2017). Salmon provides fast and bias-aware quantification of transcript expression. Nat. Methods *14*, 417–419.

112. Soneson, C., Love, M.I., and Robinson, M.D. (2015). Differential analyses for RNA-seq: transcript-level estimates improve gene-level inferences. F1000Res. *4*, 1521.

113. Satija, R., Farrell, J.A., Gennert, D., Schier, A.F., and Regev, A. (2015). Spatial reconstruction of single-cell gene expression data. Nat. Biotechnol. *33*, 495–502.

STAR★METHODS

KEY RESOURCES TABLE

REAGENT or RESOURCE	SOURCE	IDENTIFIER
**Antibodies**		
Fc Receptor Binding Inhibitor Polyclonal Antibody, Functional Grade, eBioscience™	Thermo Fisher Scientific	Cat#16-9161-73; RRID: AB_469272
BD Pharmingen™ FITC Mouse Anti-Human CD3	BD Bioscience	Cat#561806; RRID: AB_11154397
A CD4 Monoclonal Antibody (RPA-T4), APC, eBioscience™	Thermo Fisher Scientific	Cat#17-0049-42; RRID: AB_1272048
PE anti-human CD19 Antibody	BioLegend	Cat#302207; RRID: AB_314237
**Chemicals, peptides, and recombinant proteins**		
Ficoll-Paque Plus	Cytiva	Cat#17144003
LIVE/DEAD Fixable Near-IR Dead Cell Stain Kit	Thermo Fisher Scientific	Cat#L34975
**Critical commercial assays**		
Chromium Next GEM Chip K	10x Genomics	PN-1000286
Chromium Next GEM Single Cell 5′ Kit v2	10x Genomics	PN-1000263
Chromium Single Cell Human TCR Amplification Kit	10x Genomics	PN-1000252
**Deposited data**		
scRNA-seq data	This paper	JGA: JGAS000578 https://singlecell.broadinstitute.org/single_cell/study/SCP1963
scRNA-seq data for MG	Yasumizu et al.[7]	JGA: JGAS000482
ImmuNexUT	Ota et al.[39]	GEA: E-GEAD-397
European LD scores	Jagadeesh et al.[88]	https://storage.googleapis.com/broad-alkesgroup-public/LDSCORE/Dey_Enhancer_MasterReg/processed_data,
Roadmap_U_ABC for blood	Jagadeesh et al.[88]	https://storage.googleapis.com/broad-alkesgroup-public/LDSCORE/DeepLearning/Dey_DeepBoost_Imperio/data_extra/AllPredictions.AvgHiC.ABC0.015.minus150.withcolnames.ForABCPaper.txt.gz
scCITE-seq data	Hao et al.[62]	GEO: GSE164378
public scRNA-seq data	See Table S6	See Table S6
**Software and algorithms**		
Code and algorithms for analysis	This paper	https://github.com/yyoshiaki/autoimmune10x_CD4T_Manuscript_2023 (https://doi.org/10.5281/zenodo.10154467) https://github.com/yyoshiaki/sclinker-skg (https://doi.org/10.5281/zenodo.10200443)
NMFproj	This paper	https://github.com/yyoshiaki/NMFprojection (https://doi.org/10.5281/zenodo.10200434)
Pipeline for single-cell reference mapping used in meta-analysis	This paper	https://github.com/yyoshiaki/screfmapping (https://doi.org/10.5281/zenodo.10200424)
Cell Ranger	10x Genomics	v4.0.0
Scanpy	Wolf et al.[100]	https://scanpy.readthedocs.io/en/stable/
Harmony	Korsunsky et al.[13]	https://github.com/immunogenomics/harmony
scikit-learn	Pedregosa et al.[101]	https://zenodo.org/records/10039710
ikra	Hiraoka et al.[102]	https://zenodo.org/records/3352573

(Continued on next page)

Continued

REAGENT or RESOURCE	SOURCE	IDENTIFIER
clusterProfiler	Yu et al.[103]	https://github.com/YuLab-SMU/clusterProfiler
ReactomePA	Yu et al.[104]	https://github.com/YuLab-SMU/ReactomePA
scirpy	Sturm et al.[105]	https://github.com/scverse/scirpy
TiRP	Lagattuta et al.[28]	https://github.com/immunogenomics/TiRP
S-LDSC	Bulik-Sullivan et al.[106]	https://github.com/bulik/ldsc
CellTypist	Domínguez Conde et al.[36]	https://github.com/Teichlab/celltypist
projecTILs	Andreatta et al.[16]	https://github.com/carmonalab/ProjecTILs
Azimuth	Hao et al.[62]	https://azimuth.hubmapconsortium.org/
Seurat	Hao et al.[62]	https://github.com/satijalab/seurat
Symphony	Kang et al.[14]	https://github.com/immunogenomics/symphony
pyCirclize	N/A	https://github.com/moshi4/pyCirclize
imbalanced-learn	Lemaitre et al.[107]	https://github.com/scikit-learn-contrib/imbalanced-learn
LightGBM	Ke et al.[108]	https://github.com/Microsoft/LightGBM
statsmodels	Seabold et al.[109]	https://github.com/statsmodels/statsmodels

RESOURCE AVAILABILITY

Lead contact

Further information and requests for resources and reagents should be directed to and will be fulfilled by the lead contacts, Naganari Ohkura (nohkura@ifrec.osaka-u.ac.jp).

Materials availability

This study did not generate new unique reagents.

Data and code availability

- The raw sequence data for single-cell RNA-seq analysis have been deposited at Japanese Genotype-phenotype Archive (JGA) under accession number JGAS000482 (https://ddbj.nig.ac.jp/resource/jga-study/JGAS000482) and JGAS000578 (https://ddbj.nig.ac.jp/resource/jga-study/JGAS000578). Single-cell data can be explored interactively and downloaded in SingleCellPortal (https://singlecell.broadinstitute.org/single_cell/study/SCP1963). Detailed results of the meta-analysis and sc-linker were deposited at https://yyoshiaki.github.io/autoimmune_scRNAseq/Tcells.html and https://doi.org/10.6084/m9.figshare.23983038.
- All original code is deposited at https://doi.org/10.5281/zenodo.10154467 and in the GitHub repository https://github.com/yyoshiaki/autoimmune10x_CD4T_Manuscript_2023 (https://doi.org/10.5281/zenodo.10154467) and https://github.com/yyoshiaki/sclinker-skg (https://doi.org/10.5281/zenodo.10200443). NMFproj is provided at https://github.com/yyoshiaki/NMFprojection (https://doi.org/10.5281/zenodo.10200434). The pipeline for reference mapping used in meta-analysis is provided at https://github.com/yyoshiaki/screfmapping (https://doi.org/10.5281/zenodo.10200424)
- Any additional information required to reanalyze the data reported in this paper is available from the lead contacts upon request.

EXPERIMENTAL MODEL AND STUDY PARTICIPANT DETAILS

Human samples

The study using human samples was reviewed and approved by the Research Ethics Committee of Osaka University and carried out in accordance with the guidelines and regulations. Human samples were collected under approved Osaka University's review board protocols: ID 708-10. Written informed consent was obtained from all donors. Patient information is provided as a supplementary table (Table S1).

METHOD DETAILS

Cell preparation and sequencing of scRNA-seq

From blood collected using heparin-coated tubes, we first collected PBMCs using Ficoll-Paque Plus (Cytiva). PBMCs were washed, blocked with Fc Receptor Binding Inhibitor Polyclonal Antibody, Functional Grade, eBioscience (Thermo Fisher Scientific), and stained with FITC-labeled anti-CD3 mAb (dilution: 1/100, UCHT1, BD Bioscience), APC-labeled anti-CD4 mAb (dilution: 1/100,

RPA-T4, Thermo Fisher Scientific), PE-labeled anti-CD19 mAb (HIB19, BioLegend), Live/Dead (Thermo Fisher Scientific). Live-$CD3^{+}CD4^{+}CD19^{-}$ cells were isolated using BD Biosciences FACS Aria II or BD Biosciences FACS Aria III. $CD4^{+}$ T cells and B cells were mixed in equal numbers in some samples.

The sorted cells were loaded to Chromium Next GEM Chip K (10x Genomics) on Chromium Controller (10x Genomics) for barcoding and cDNA synthesis. The library construction was performed using Chromium Next GEM Single Cell 5′ Kit v2 and Chromium Single Cell Human TCR Amplification Kit (10x Genomics) for 5′ according to the manufacturer's protocol. The libraries were sequenced on NovaSeq6000 (Illumina).

Preprocess of scRNA-seq data

Sequenced reads were processed using Cell Ranger (v4.0.0) with pre-built reference refdata-gex-GRCh38-2020-A and refdata-cell-ranger-vdj-GRCh38-alts-ensembl-4.0.0 downloaded at 10x GENOMICS' Website. Quantified expressions were preprocessed and visualized using Scanpy 1.8.1[100] and Python 3.8.0. For $CD4^{+}$ T cell and B cell mixed samples, we extracted only $CD4^{+}$ T cells as following procedures. Briefly, we normalized (sc.pp.normalize_total) gene expression, log-transformed it (sc.pp.log1p), extracted highly variable genes (HVGs) (sc.pp.highly_variable_genes with min_mean = 0.0125, max_mean = 3, min_disp = 0.5), computed PCA (sc.tl.pca) and neighbors (sc.pl.neighbors with n_neighbors = 10, n_pcs = 40), computed clusters using Leiden algorithm (sc.tl.leiden), and embedded using UMAP algorithm (sc.tl.umap). CD3E-positive and MS4A1-negative clusters were extracted as $CD4^{+}$ T cells and used for the analysis. Cells with mitochondrial genes were higher than 10%, detected genes less than 200, or annotated as multichain by scirpy were filtered out. Variable genes of TCR alpha and beta were removed for the clustering and embedding to remove the effect of clonal expansion. Gene expressions were preprocessed by sc.pp.normalize_per_cell with counts_per_cell_after = 1e4, sc.pp.log1pp, retained HVGs. The inference of the cell cycle was performed using the sc.tl.score_genes_cell_cycle function following the tutorial (https://nbviewer.jupyter.org/github/theislab/scanpy_usage/blob/master/180209_cell_cycle/cell_cycle.ipynb). Total counts of UMI, % mitochondrial genes, S score, G2M score were regressed out using sc.tl.regress_out and scaled using sc.tl.scale. Then, principal components were computed using sc.tl.pca. The batch effect of samples was removed by the Harmony algorithm.[13] Cells were embedded by UMAP using sc.tl.umap (spread = 1.5), and clustered using sc.tl.leiden (resolution = 1.2). Re-clustering and embedding were performed using sc.tl.umap (spread = 1.5), clustered using sc.tl.leiden (resolution = 1.7) after removing clusters containing doublets with B cells, monocyte lineages, etc. We defined cluster L1 as a large classification using the leiden clusters. Next, for some clusters, concatenation or re-clustering was performed with sc.tl.leiden (resolution 0.3–1) to divide clusters at the minimum resolution with distinct marker genes. We defined cluster L2 as a smaller classification. Marker genes were determined using sc.tl.rank_genes_groups with method = 't-test_overestim_var'. The silhouette scores were calculated using the silhouette_samples function from scikit-learn (v0.24.2) with the "euclidean" metric.

Integration with bulk RNA-seq dataset

Fastq files were processed using an RNA-seq integrative pipeline, ikra (v2.0.1),[102] composed of Trim Galore! 0.6.7,[110] Salmon 1.4.0,[111] tximport 1.6.0[112] with the reference Gencode M26 for mice and 37 for humans. Datasets for which the TPM matrix was provided were downloaded and used directly for analyses. For the ImmuNexUT (E-GEAD-397) dataset, the downloaded count matrix was converted to TPM.

For the correlations between bulk RNA-seq and scRNA-seq datasets, TPM or scaledTPM expression matrix of bulk RNA-seq were normalized using sc.pp.normalize_per_cell (counts_per_cell_after = 1e4), sc.pp.log1p, sc.pp.scale (max_value = 10), concatenated to scRNA-seq object, and calculated the correlations using sc.tl.dendrogram with the default parameters.

Gene expression decomposition using NMF

To decompose cellular processes, we applied NMF implemented in scikit-learn (v0.24.2) to normalize gene expression of HVGs. Using the non-negative matrix factorization (NMF) method, the normalized gene expression matrix X, with elements represented as x_{ij}, was decomposed into a gene feature matrix W (with elements represented as w_{ic}) and a cell feature matrix H (with elements represented as h_{cj}). In this context, i refers to the gene index, j represents the cell index, and c denotes the component. The decomposition is given by:

$$X = W \cdot H$$

For our analysis, the number of components was determined to be 12 based on two criteria: i) it exceeded the elbow in the distribution of explained variance and ii) it was just before a sharp increase in the maximum inter-components Spearman's correlation. The explained variance for the given number of components c is denoted as $Evar_c$ and is derived using the residual sum of squares (RSS) for component c, represented as RSS_c :

$$RSS_c = \sum_{ij} (x_{ij} - w_{ic} h_{cj})^2$$

The explained variance $Evar_c$ is then:

$$Evar_c = 1 - \frac{RSS_c}{\sum_{ij} x_{ij}^2}$$

Additionally, $Evar_{all}$, which represents the overall explained variance across all components, is computed from the residuals of X and $W \cdot H$, denoted as RSS_{all}:

$$RSS_{all} = \sum_{ij} (x_{ij} - w_i.h._j)^2$$

$$Evar_{all} = 1 - \frac{RSS_{all}}{\sum_{ij} x_{ij}^2}$$

For the pathway enrichment analysis, we extracted the top 100 genes with the highest feature value for each component and converted gene symbols into Entrezid using the bitr function provided by clusterProfiler (3.16.1)[103] and computed enriched Reactome pathways using the compareCluster function of clusterProfiler with the enrichPathway function in ReactomePA (1.32.0). For the projection of gene features defined by NMF, we performed NMF with pre-computed W using scikit-learn. The matrix W was converted to mouse genes using a list of human and mouse homologs provided at http://www.informatics.jax.org/homology.shtml. For genes with multiple homologs, one of the genes was retained. For the NMF calculation, only overlapped genes were used. To examine whether the selected HVGs of fixed W can capture HVGs in a query dataset, we calculated the proportion of the number of HVGs included in fixed W against the number of HVGs in the query dataset as POH. In the CD4+ T dataset, we determined that if the POH is below 0.1, which is the conservative threshold from the distribution of the null hypothesis (Figure S3C), there is a variance that cannot be represented by the NMFproj. sc.pp.highly_variable_genes in scanpy with the following parameters; min_mean = 0.0125, max_mean = 3, min_disp = 0.1 was used for the calculation of HVGs of the query datasets and selected top 500 genes regarding normalized dispersion[113] with the exclusion of VDJ genes of TCR and IG. A framework for NMF projection is available at https://github.com/yyoshiaki/NMFprojection.

For the analysis of ImmuNexUT, the count data was downloaded at (https://humandbs.biosciencedbc.jp/en/hum0214-v3) and converted to TPM. We removed BD, AAV, AOSD, and SjS samples from the analysis because these samples are processed in a different procedure from other samples. TPM matrix was decomposed with the pre-computed gene feature matrix using NMFproj. Extracted NMF feature H was tested using a multiple linear regression provided as the formula.api.ols function by a python package statsmodels (0.12.0) with a model, NMF_i ~ Disease + Age + Gender +1.

TCR analysis

For TCR analysis, we used the standard pipeline of scirpy 0.10.1[105] according to the official tutorial (https://scverse.org/scirpy/latest/tutorials/tutorial_3k_tcr.html). Briefly, clones were defined by clonotypes using scirpy.tl.define_clonotypes with parameters; receptor_arms = "all", dual_ir = "primary_only". Repertoire similarities were measured using the function scirpy.tl.repatoire_overlap. TiRP score was calculated according to the instruction in the repository (https://github.com/immunogenomics/TiRP.git).

Heritability partitioning

To assess the contribution of each cell-type-specific gene expression and the NMF component, we applied S-LDSC with Roadmap ABC-immune enhancer-gene linking strategy implemented using an sc-linker pipeline[88] with slight modifications (https://github.com/yyoshiaki/sclinker-skg). We only used HVGs defined by the preprocessing section in the analysis. We used min-max scaled gene scores for the cell-type gene programs as the gene weights. For NMF components, we used the gene feature matrix W with the min-max scaling for the gene weights. Using the gene weights, LD scores for each category were calculated with European LD scores used in the article[88] and Roadmap_U_ABC for blood (https://storage.googleapis.com/broad-alkesgroup-public/LDSCORE/Dey_Enhancer_MasterReg/processed_data, https://storage.googleapis.com/broad-alkesgroup-public/LDSCORE/DeepLearning/Dey_DeepBoost_Imperio/data_extra/AllPredictions.AvgHiC.ABC0.015.minus150.withcolnames.ForABCPaper.txt.gz). In addition to the sumstats files provided in gs://broad-alkesgroup-public/LDSCORE/all_sumstats, we used several additional sumstats by processing using munge_sumstats.py in LDSC v1.0.1 (Table S10). S-LDSC was performed with the baseline-LD model v2.1 (https://storage.googleapis.com/broad-alkesgroup-public/LDSCORE/1000G_Phase3_baselineLD_v2.1_ldscores.tgz). The Enrichment score (*E*-score) was calculated as the difference between the enrichment for annotation in a particular program against an SNP annotation for all protein-coding genes with a predicted enhancer-gene link in the blood. We also used FDR calculated from the p value of Enrichment outputted by S-LDSC.

Comparison of our ref-mapping classification performance with other classification tools

To compare the performance of our ref-mapping with other tools, we applied various cell classifiers to the same dataset. For this purpose, we utilized the pre-annotated scCITE-seq data generated by Hao et al.[62] on PBMC and extracted circulating CD4+ T cells. The procedures for the reference mapping by Symphony using our reference were described in the subsequent meta-analysis section. For CellTypist,[36] following the tutorial (https://www.celltypist.org/tutorials), we normalized gene expression (total count = 10000) and log-transformed it and then applied CellTypist. For the CellTypist model, we used the model built on the human PBMC data (COVID-19_HumanChallenge_Blood). Regarding projecTILs,[16] the dataset was normalized gene expression count (total

count = 10000), log-transformed, and performed labal transfer following the tutorial (https://github.com/carmonalab/ProjecTILs). For the transfer, we used references built with human tumor-infiltrating CD4+ T cells (CD4T_human_ref_v1.rds).

Meta-analysis of CD4+ T cells from public datasets

We collected scRNA-seq data from PBMC generated by 10x platforms, Seq-Well or SPLiT-seq (Parse Biosciences WT Mega)[6,8,40–61] (Table S6). If the count matrix was available, we used the quantified matrix. Otherwise, we quantified the expression using Cell Ranger with pre-built reference refdata-gex-GRCh38-2020-A. As the sample QC, samples with XIST mean expression (count) > 0.05 were inferred as female. If the inferred gender and metadata differed, we removed the sample from the analysis. We extracted CD4+ T cells from published data of PBMCs using Azimuth 0.4.4.[62] We created Seurat Object using the CreateSeuratObject function implemented in Seurat 4.1.0[62] with parameters min.cells = 3, min.features = 200. We also filtered out the cells that express ≧10% mitochondrial genes in their total gene expression. We normalized the expression using SCTransform with parameters method = "glmGamPoi", ncells = 2000, n_genes = 2000, do.correct.umi = FALSE. In this procedure, we used Azimuth reference data v1.0.0 human_pbmc loaded from the website (https://seurat.nygenome.org/azimuth/references/v1.0.0/human_pbmc). We found anchors between query data and Azimuth reference data (FindTransferAnchors with parameters k.filter = NA, normalization.method = "SCT", dims = 1:50, n.trees = 20, mapping.score.k = 100), transferred cell type labels (TransferData with parameters dims = 1:50, n.trees = 20) and calculated the embeddings on the reference supervised PCA (IntegrateEmbeddings with the default options) and neighbors (FindNeighbors with parameter l2.norm = TRUE). We transformed an NN index (NNTransform with the default parameters) and projected the query data to the reference UMAP (RunUMAP with the default parameters). We visualized query data by DimPlot, DotPlot, and FeaturePlot.

Next, we mapped extracted cells on our reference using symphony 0.1.0[14] following the vignettes (https://github.com/immunogenomics/symphony/blob/main/vignettes/Seurat.ipynb). First, we created a symphony reference using our dataset. Our scanpy object saved as an h5ad file was converted to h5Seurat using SeuratDisk and loaded as a Seurat object. We used only HVGs for symphony reference to reduce batch effect strictly. Then, the object was preprocessed as follows; SCTransform (method = "glmGamPoi"), ScaleData, RunPCA, RunHarmony.Seurat (group.by = "sample"), FindNeighbors (dims = 1:30), RunUMAP2, and buildReferenceFromSeurat. For query mapping, extracted CD4+ T cells were normalized (SCTransform with parameter method = "glmGamPoi") and mapped (mapQuery with parameter do_normalize = FALSE, vars = "batch") with batch correction against each sample. The cluster L1 and L2 assignments were performed using the knnPredict.Seurat function. We visualized the mapping results by DimPlot and FeaturePlot as the quality control. We are providing the label transfer pipeline at https://github.com/yyoshiaki/screfmapping.

In the analysis, we performed a generalized linear model (GLM) independently for each subtype in cluster L2. For each cell population in a given donor, we define *n_cat* as the number of cells in that population, and *n_total* as the total number of cells in that donor. The response variable for the binomial regression is thus composed of *n_cat* and *n_total - n_cat*, representing the number of cells in the specific population and the number not in the population, respectively. The *Disease* variable was one-hot encoded where healthy was set as the baseline. The *Age* variable was scaled by dividing by 25 to match the scale of the other variables in the model. Therefore, the binomial regression was performed with the formula: (*n_cat*, *n_total - n_cat*) ~ *Disease* + *Age*/25 + *Gender* + *Project* using the R glm function. This model allows us to assess the effects of disease, age, and gender on the frequency of each cell subtype within a given donor. Similarly, for the analysis of enriched NMF components, we first calculated cell profiles using NMFproj with raw counts, and linear regression was performed for each NMF component in each cell type respectively with the formula; *NMF_i* ~ *Disease* + *Age*/25 + *Gender* + *Project* using the R glm function. The Chord diagrams were created using pyCirclize (0.1.3). For the PCA plot of individuals, principal components were calculated using R prcomp function (scale = TRUE) against cell frequencies of each sample. PC loadings for cell subsets were visualized using R biplot function.

Machine learning for the prediction of autoimmune states

To address potential study-specific effects, we ensured a clear division in our datasets: projects used as the training set, while independent projects, with no overlaps, were assigned as the test set. This guarantees the classifier's evaluation on genuinely unseen data from a separate study. Cell frequencies and/or NMFproj values in Tcm (Th0) and Tnaive were scaled using StandardScaler (scikit-learn 1.0.2). Note that Tnaive and Tcm (Th0), which have a high degree of nodes in the network, were selected for the NMFproj results to keep the number of parameters low. NMF values were imputed using SimpleImputer (scikit-learn 1.0.2) with parameter strategy = 'most_frequent' trained by training datasets. For the binary classification, we used the LogisticRegression in scikit-learn with the default parameters. For the multiclass classification, the label imbalance was corrected using SMOTE (imbalanced-learn 0.9.1) with parameter sampling_strategy = 'all'. Then, LightGBM 3.3.2[108] was used for the model with parameters, 'objective' = 'multiclass' and 'early_stopping_rounds' = 10.

QUANTIFICATION AND STATISTICAL ANALYSIS

All statistical analyses were performed in R (4.0.3 or 4.1.2) and Python (3.8.0). FDR was obtained by the Benjamini-Hochberg procedure implemented by a Python package statsmodels (0.12.0). All other statistical analyses are detailed in the respective sections of the article.

Cell Genomics, Volume *4*

Supplemental information

Single-cell transcriptome landscape of circulating CD4$^+$ T cell populations in autoimmune diseases

Yoshiaki Yasumizu, Daiki Takeuchi, Reo Morimoto, Yusuke Takeshima, Tatsusada Okuno, Makoto Kinoshita, Takayoshi Morita, Yasuhiro Kato, Min Wang, Daisuke Motooka, Daisuke Okuzaki, Yamami Nakamura, Norihisa Mikami, Masaya Arai, Xuan Zhang, Atsushi Kumanogoh, Hideki Mochizuki, Naganari Ohkura, and Shimon Sakaguchi

Figure S1

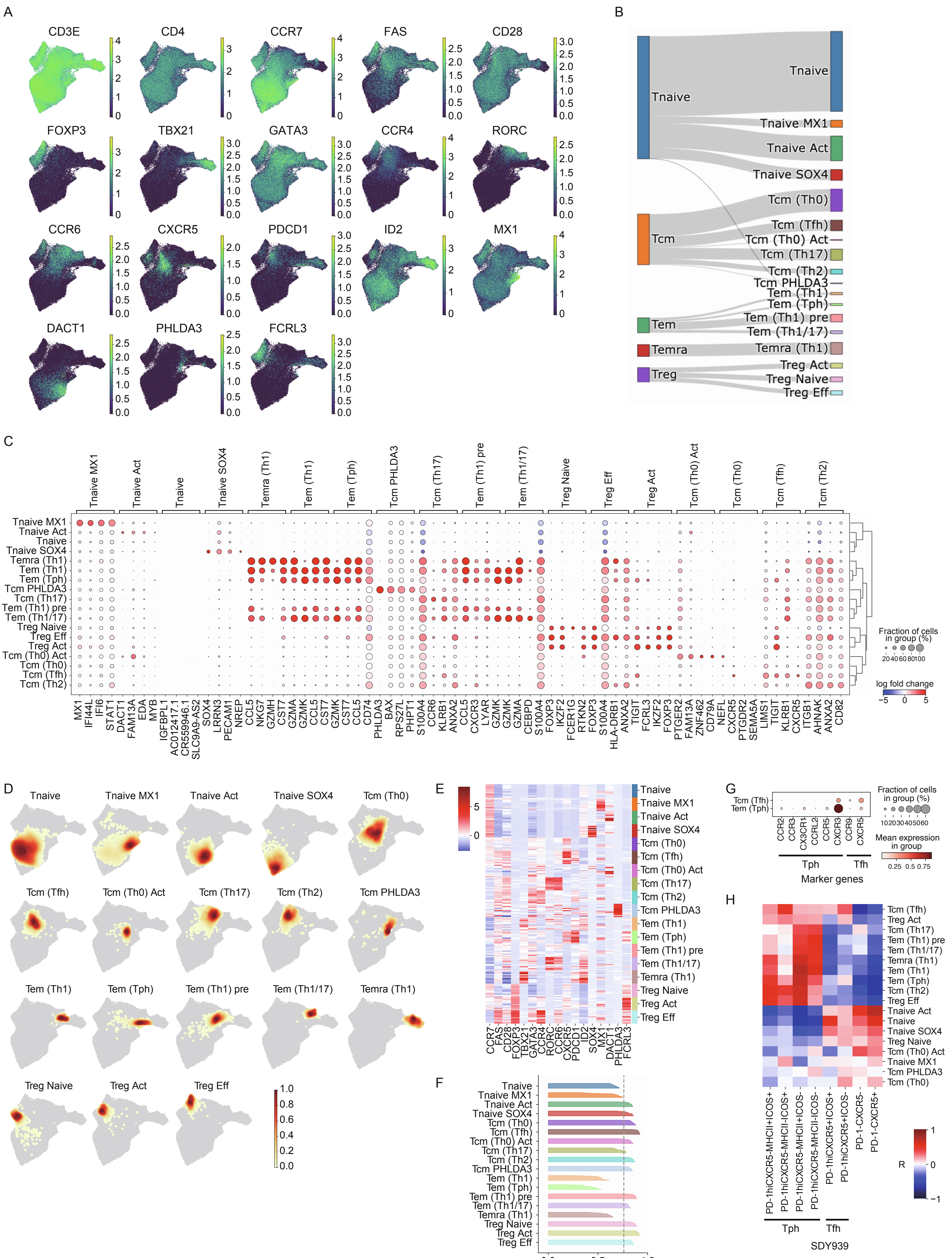

A
CD3E
CD4
CCR7
FAS
CD28
FOXP3
TBX21
GATA3
CCR4
RORC
CCR6
CXCR5
PDCD1
ID2
MX1
DACT1
PHLDA3
FCRL3
B
Tnaive
Tcm
Tem
Temra
Treg
Tnaive MX1
Tnaive Act
Tnaive SOX4
Tcm (Th0)
Tcm (Tfh)
Tcm (Th0) Act
Tcm (Th17)
Tcm (Th2)
Tcm PHLDA3
Tem (Th1)
Tem (Tph)
Tem (Th1) pre
Tem (Th1/17)
Temra (Th1)
Treg Act
Treg Naive
Treg Eff
C
Fraction of cells in group (%)
log fold change
MX1 IFI44L IFI6 STAT1 DACT1 FAM13A EDA MYB IGFBPL1 AC012417.1 CR559946.1 SLC9A9-AS2 SOX4 LRRN3 PECAM1 NREP CCL5 NKG7 GZMH CST7 GZMA GZMK CCL5 CST7 GZMK CST7 CCL5 CD74 PHLDA3 BAX RPS27L PHPT1 S100A4 CCR6 KLRB1 ANXA2 CCL5 CXCR3 LYAR GZMK GZMK GZMA CEBPD S100A4 FOXP3 IKZF2 FCER1G RTKN2 FOXP3 S100A4 HLA-DRB1 ANXA2 TIGIT FCRL3 IKZF2 FOXP3 PTGER2 FAM13A ZNF462 CD79A NEFL CXCR5 PTGDR2 SEMA5A LIMS1 TIGIT KLRB1 CXCR5 ITGB1 AHNAK ANXA2 CD82
D
E
CCR7 FAS CD28 FOXP3 TBX21 GATA3 CCR4 RORC CCR6 CXCR5 PDCD1 SOX4 ID2 MX1 DACT1 PHLDA3 FCRL3
F
The silhouette coefficient
G
CCR2 CCR3 CX3CR1 CCRL2 CCR5 CXCR3 CCR9 CXCR5
Tph
Tfh
Marker genes
Mean expression in group
H
PD-1hiCXCR5-MHCII+ICOS+
PD-1hiCXCR5-MHCII-ICOS+
PD-1hiCXCR5-MHCII+ICOS-
PD-1hiCXCR5-MHCII-ICOS-
PD-1hiCXCR5+ICOS+
PD-1hiCXCR5+ICOS-
PD-1-CXCR5-
PD-1-CXCR5+
R
SDY939

Figure S1. Global characterization of $CD4^+$ T cells, related to Figure 1

(A) UMAP plots depicting gene expressions of marker genes. (B) Sankey diagrams showing cluster assignment of cells in clusters L1 and L2. (C) Dot plot depicting signature genes' mean expression levels and percentage of cells expressing them across clusters. Marker genes for the plot were calculated by pairwise comparison with a group and the other groups iteratively using scanpy.tl.rank_genes_groups function. (D) Density plot of cell distributions for cluster L2 populations. (E) Heatmaps of marker genes' mean expression for each subtype in each sample. (F) Silhouette scores for each cluster for each sample based on Harmonized PCA. This demonstrates the distinctiveness of each cluster in every sample. (G) Dot plot depicting T peripheral helper (Tph) and T follicular helper (Tfh) marker genes' mean expression levels and percentage of cells expressing them in Tcm (Tfh) and Tem (Tph). (H) Pearson's correlation of transcriptome profiles between sorted T cell fractions, including Tph (SDY939) and our scRNA-seq (cluster L2).

Figure S2

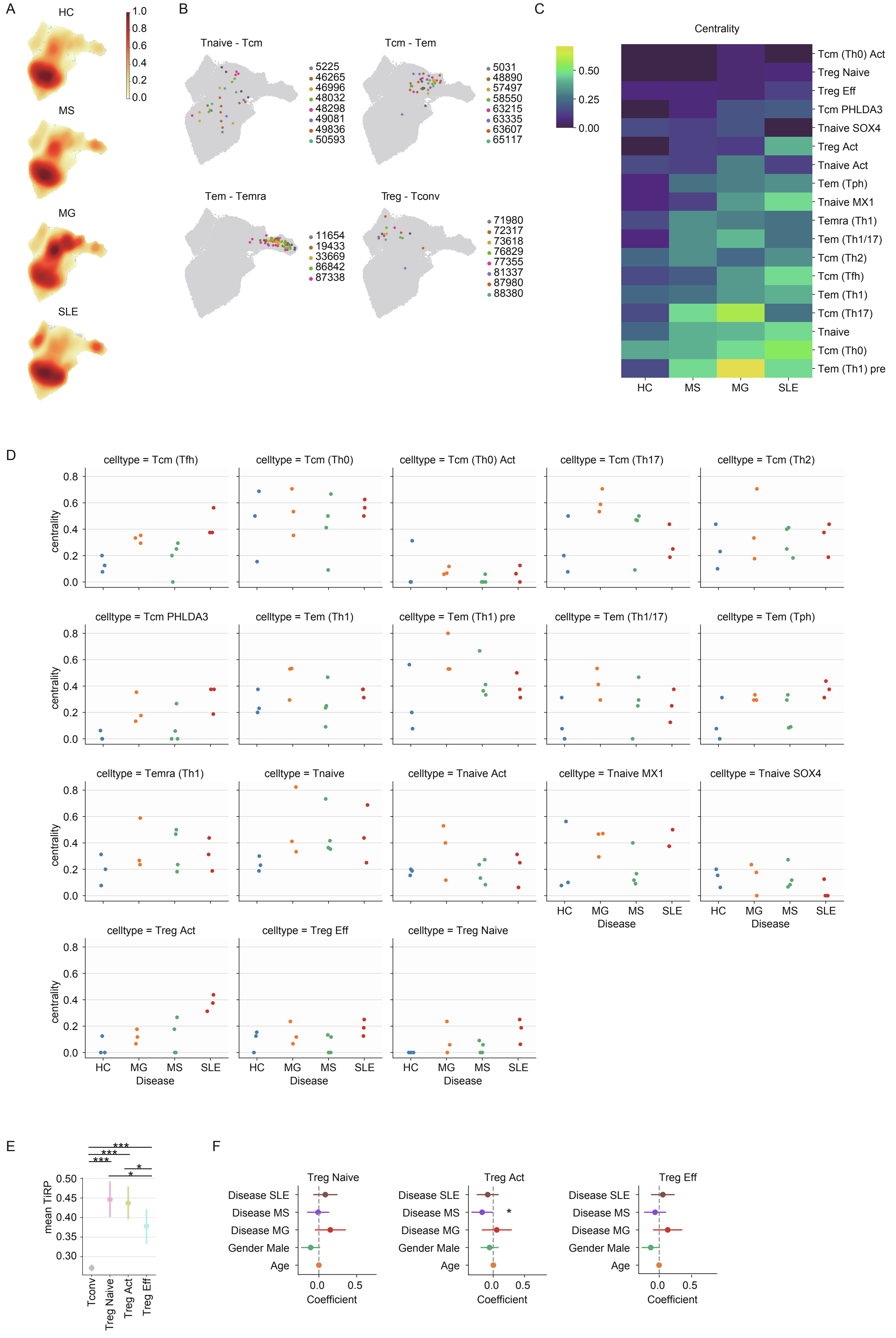

A
HC
MS
MG
SLE
1.0
0.8
0.6
0.4
0.2
0.0
B
Tnaive - Tcm
5225
46265
46996
48032
48298
49081
49836
50593
Tcm - Tem
5031
48890
57497
58550
63215
63335
63607
65117
Tem - Temra
11654
19433
33669
86842
87338
Treg - Tconv
71980
72317
73618
76829
77355
81337
87980
88380
C
Centrality
0.50
0.25
0.00
Tcm (Th0) Act
Treg Naive
Treg Eff
Tcm PHLDA3
Tnaive SOX4
Treg Act
Tnaive Act
Tem (Tph)
Tnaive MX1
Temra (Th1)
Tem (Th1/17)
Tcm (Th2)
Tcm (Tfh)
Tem (Th1)
Tcm (Th17)
Tnaive
Tcm (Th0)
Tem (Th1) pre
HC
MS
MG
SLE
D
celltype = Tcm (Tfh)
celltype = Tcm (Th0)
celltype = Tcm (Th0) Act
celltype = Tcm (Th17)
celltype = Tcm (Th2)
celltype = Tcm PHLDA3
celltype = Tem (Th1)
celltype = Tem (Th1) pre
celltype = Tem (Th1/17)
celltype = Tem (Tph)
celltype = Temra (Th1)
celltype = Tnaive
celltype = Tnaive Act
celltype = Tnaive MX1
celltype = Tnaive SOX4
celltype = Treg Act
celltype = Treg Eff
celltype = Treg Naive
centrality
0.8
0.6
0.4
0.2
0.0
HC
MG
MS
SLE
Disease
E
mean TiRP
0.50
0.45
0.40
0.35
0.30
***
***
***
*
*
Tconv
Treg Naive
Treg Act
Treg Eff
F
Treg Naive
Treg Act
Treg Eff
Disease SLE
Disease MS
Disease MG
Gender Male
Age
*
0.0
0.5
Coefficient

Figure S2. Centralities of TCR networks vary depending on the diseases, related to Figure 1

(A) Density plot of cell distributions for each disease. (B) Representative clones spanning across clusters. (C) Degree centrality of TCR networks for cluster L2. The average of each disease was shown. (D) Individual value of degree centrality of TCR networks. (E) Distribution of mean TiRP scores across Treg clusters. Pairwise Tukey-HSD posthoc tests. The multiple test correction was performed using a two-stage FDR strategy. *: $p_{adj} < 0.05$, **: $p_{adj} < 0.01$, ***: $p_{adj} < 0.001$. (F) Changes in TiRP scores in Treg clusters associated with disease states, age, and sex. The estimated coefficients and the 95 percentiles by multiple linear regression were plotted.

Figure S3

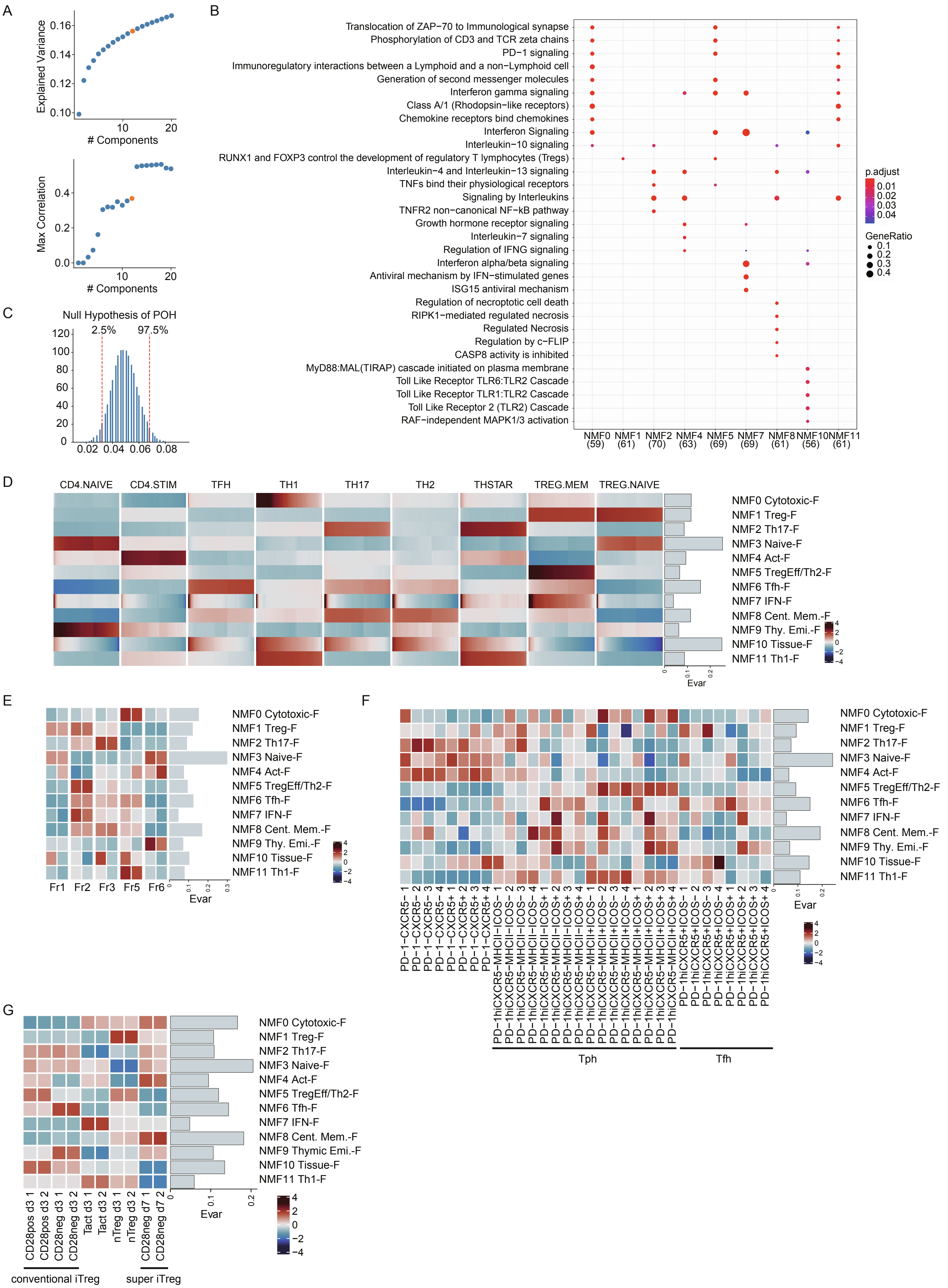
A
Explained Variance
Components
Max Correlation
Components
B
Translocation of ZAP−70 to Immunological synapse
Phosphorylation of CD3 and TCR zeta chains
PD−1 signaling
Immunoregulatory interactions between a Lymphoid and a non−Lymphoid cell
Generation of second messenger molecules
Interferon gamma signaling
Class A/1 (Rhodopsin−like receptors)
Chemokine receptors bind chemokines
Interferon Signaling
Interleukin−10 signaling
RUNX1 and FOXP3 control the development of regulatory T lymphocytes (Tregs)
Interleukin−4 and Interleukin−13 signaling
TNFs bind their physiological receptors
Signaling by Interleukins
TNFR2 non−canonical NF−kB pathway
Growth hormone receptor signaling
Interleukin−7 signaling
Regulation of IFNG signaling
Interferon alpha/beta signaling
Antiviral mechanism by IFN−stimulated genes
ISG15 antiviral mechanism
Regulation of necroptotic cell death
RIPK1−mediated regulated necrosis
Regulated Necrosis
Regulation by c−FLIP
CASP8 activity is inhibited
MyD88:MAL(TIRAP) cascade initiated on plasma membrane
Toll Like Receptor TLR6:TLR2 Cascade
Toll Like Receptor TLR1:TLR2 Cascade
Toll Like Receptor 2 (TLR2) Cascade
RAF−independent MAPK1/3 activation
NMF0 (59) NMF1 (61) NMF2 (70) NMF4 (63) NMF5 (69) NMF7 (69) NMF8 (61) NMF10 (56) NMF11 (61)
p.adjust
GeneRatio
C
Null Hypothesis of POH
2.5%
97.5%
D
CD4.NAIVE CD4.STIM TFH TH1 TH17 TH2 THSTAR TREG.MEM TREG.NAIVE
NMF0 Cytotoxic-F
NMF1 Treg-F
NMF2 Th17-F
NMF3 Naive-F
NMF4 Act-F
NMF5 TregEff/Th2-F
NMF6 Tfh-F
NMF7 IFN-F
NMF8 Cent. Mem.-F
NMF9 Thy. Emi.-F
NMF10 Tissue-F
NMF11 Th1-F
Evar
E
Fr1 Fr2 Fr3 Fr5 Fr6
Evar
F
PD−1−CXCR5− PD−1−CXCR5+
PD−1hiCXCR5−MHCII−ICOS− PD−1hiCXCR5−MHCII−ICOS+ PD−1hiCXCR5−MHCII+ICOS− PD−1hiCXCR5−MHCII+ICOS+
PD−1hiCXCR5+ICOS− PD−1hiCXCR5+ICOS+
Tph
Tfh
Evar
G
NMF9 Thymic Emi.-F
CD28pos d3 1 CD28pos d3 2 CD28neg d3 1 CD28neg d3 2 Tact d3 1 Tact d3 2 nTreg d3 1 nTreg d3 2 CD28neg d7 1 CD28neg d7 2
conventional iTreg
super iTreg
Evar

Figure S3. NMF and NMF projection, related to Figure 2

(A) The statistics for the determination of the number of components. The Y-axis shows explained variance (upper) and maximum correlation of the inter-component (lower). The X-axis shows the number of components. Spearman's correlation between components of gene features was calculated. (B) Reactome pathways enriched in each gene feature. The dot size indicates the gene ratio or the fraction of genes found in the gene set, and the color indicates p_{adj}. (Table S5) (C) Histogram of the proportion of overlapped highly variable genes (POH) under the null hypothesis for this study setting. We randomly sampled 5000 POH in the null hypothesis calculated from the overlap between random 500 (number of HVGs for the calculation of POH) genes and 1271 (number of highly variable genes of $CD4^+$ T cell) genes. Red dashed lines show 2.5 and 97.5 percentiles. (D) Heatmap showing NMF values of DICE bulk RNA-seq datasets of sorted $CD4^+$ T cell fractions. Explained variance (Evar) was also shown on the right side. (E) Heatmap showing NMF values of sorted $CD4^+$ T cell fractions by Miyara's classification (JGAD000214). Explained variance (Evar) was also shown on the right side. (F) Heatmap showing NMF values of sorted Tph fractions (SDY939). Explained variance (Evar) was also shown on the right side. (G) Heatmap showing NMF values of induced Treg (iTreg) cells cultured in different conditions (DRA008294). Explained variance (Evar) was also shown on the right side.

Figure S4

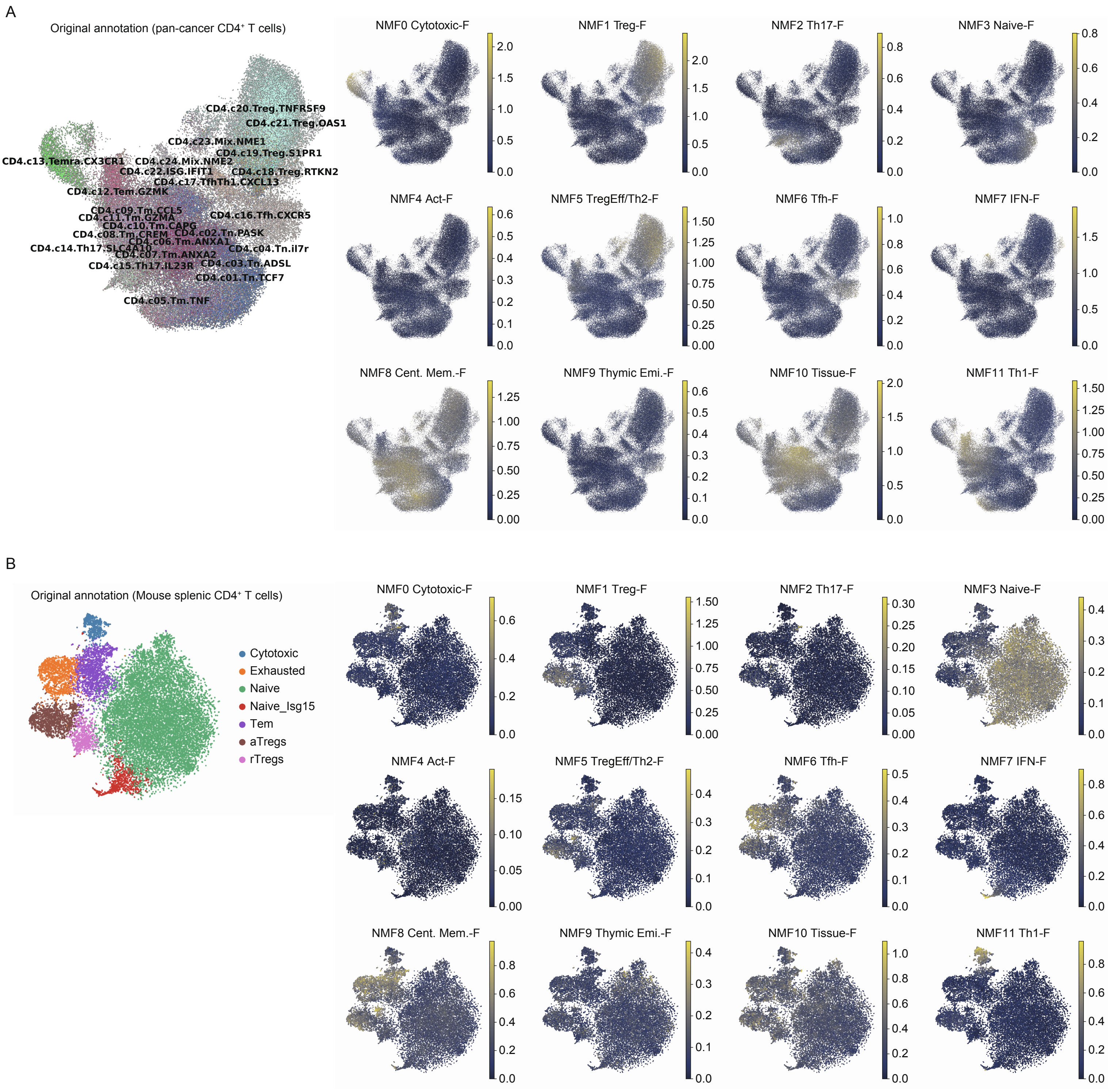
A
Original annotation (pan-cancer CD4+ T cells)
CD4.c20.Treg.TNFRSF9
CD4.c21.Treg.OAS1
CD4.c23.Mix.NME1
CD4.c19.Treg.S1PR1
CD4.c13.Temra.CX3CR1
CD4.c24.Mix.NME2
CD4.c22.ISG.IFIT1
CD4.c18.Treg.RTKN2
CD4.c17.TfhTh1.CXCL13
CD4.c12.Tem.GZMK
CD4.c09.Tm.CCL5
CD4.c11.Tm.GZMA
CD4.c16.Tfh.CXCR5
CD4.c10.Tm.CAPG
CD4.c08.Tm.CREM
CD4.c02.Tn.PASK
CD4.c06.Tm.ANXA1
CD4.c14.Th17.SLC4A10
CD4.c04.Tn.il7r
CD4.c07.Tm.ANXA2
CD4.c15.Th17.IL23R
CD4.c03.Tn.ADSL
CD4.c01.Tn.TCF7
CD4.c05.Tm.TNF
NMF0 Cytotoxic-F
NMF1 Treg-F
NMF2 Th17-F
NMF3 Naive-F
NMF4 Act-F
NMF5 TregEff/Th2-F
NMF6 Tfh-F
NMF7 IFN-F
NMF8 Cent. Mem.-F
NMF9 Thymic Emi.-F
NMF10 Tissue-F
NMF11 Th1-F
B
Original annotation (Mouse splenic CD4+ T cells)
Cytotoxic
Exhausted
Naive
Naive_Isg15
Tem
aTregs
rTregs
NMF0 Cytotoxic-F
NMF1 Treg-F
NMF2 Th17-F
NMF3 Naive-F
NMF4 Act-F
NMF5 TregEff/Th2-F
NMF6 Tfh-F
NMF7 IFN-F
NMF8 Cent. Mem.-F
NMF9 Thymic Emi.-F
NMF10 Tissue-F
NMF11 Th1-F

Figure S4. NMFproj applications in tumor-infiltrating T cells and mouse splenocytes, related to Figure 2

(A and B) UMAP plots showing original cell types (left) and projected NMF cell feature values (right) in pan-cancer tumor-infiltrating T cells scRNA-seq data (GSE156728) (A) and mouse splenic $CD4^+$ T cells (SCP490) (B).

Figure S5

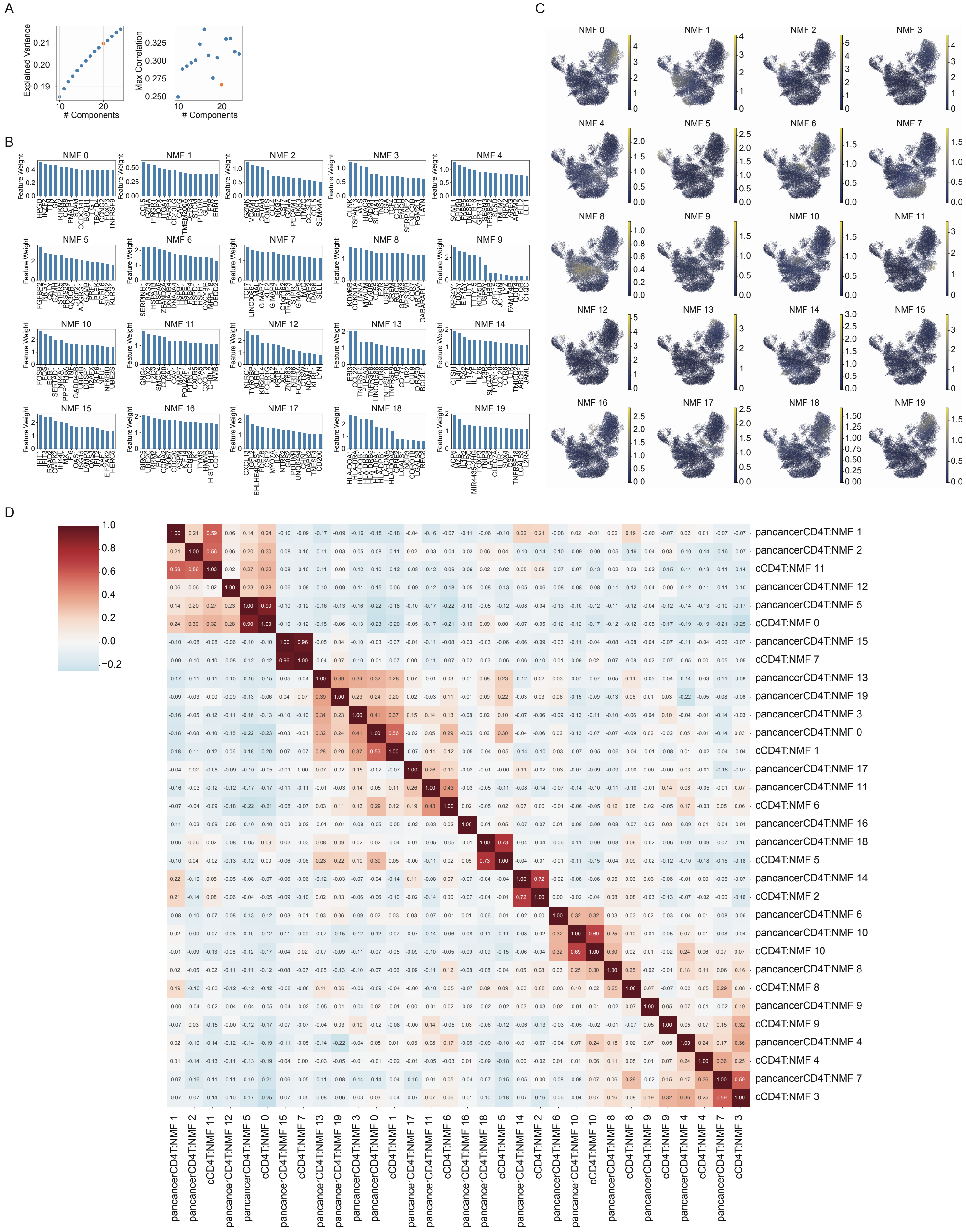
A
Explained Variance
Components
Max Correlation
Components
B
NMF 0
NMF 1
NMF 2
NMF 3
NMF 4
NMF 5
NMF 6
NMF 7
NMF 8
NMF 9
NMF 10
NMF 11
NMF 12
NMF 13
NMF 14
NMF 15
NMF 16
NMF 17
NMF 18
NMF 19
Feature Weight
C
NMF 0
NMF 1
NMF 2
NMF 3
NMF 4
NMF 5
NMF 6
NMF 7
NMF 8
NMF 9
NMF 10
NMF 11
NMF 12
NMF 13
NMF 14
NMF 15
NMF 16
NMF 17
NMF 18
NMF 19
D
pancancerCD4T:NMF 1
pancancerCD4T:NMF 2
cCD4T:NMF 11
pancancerCD4T:NMF 12
pancancerCD4T:NMF 5
cCD4T:NMF 0
pancancerCD4T:NMF 15
cCD4T:NMF 7
pancancerCD4T:NMF 13
pancancerCD4T:NMF 19
pancancerCD4T:NMF 3
pancancerCD4T:NMF 0
cCD4T:NMF 1
pancancerCD4T:NMF 17
pancancerCD4T:NMF 11
cCD4T:NMF 6
pancancerCD4T:NMF 16
pancancerCD4T:NMF 18
cCD4T:NMF 5
pancancerCD4T:NMF 14
cCD4T:NMF 2
pancancerCD4T:NMF 6
pancancerCD4T:NMF 10
cCD4T:NMF 10
pancancerCD4T:NMF 8
cCD4T:NMF 8
pancancerCD4T:NMF 9
cCD4T:NMF 9
pancancerCD4T:NMF 4
cCD4T:NMF 4
pancancerCD4T:NMF 7
cCD4T:NMF 3

Figure S5. Comparison of NMF gene features defined in circulating $CD4^+$ T cells and pan-cancer $CD4^+$ T cells, related to Figure 2

(A) The statistics for the determination of the number of components for pan-cancer $CD4^+$ T cell dataset. The Y-axis shows explained variance (left) and maximum correlation of the inter-component (right). The X-axis shows the number of components. Spearman's correlation between components of gene features was calculated. 20 was selected for the number of components. (B) Bar plots showing gene feature weight for top 15 genes. (C) Cell feature value is shown on the UMAP plots. (D) Heatmap showing the correlation of gene features between the NMF gene feature value defined in peripheral blood $CD4^+$ T cells and the newly calculated gene feature value in a pan-cancer $CD4^+$ T dataset. For the two weight matrices, only common genes were selected, and the Pearson correlation coefficients were calculated.

Figure S6

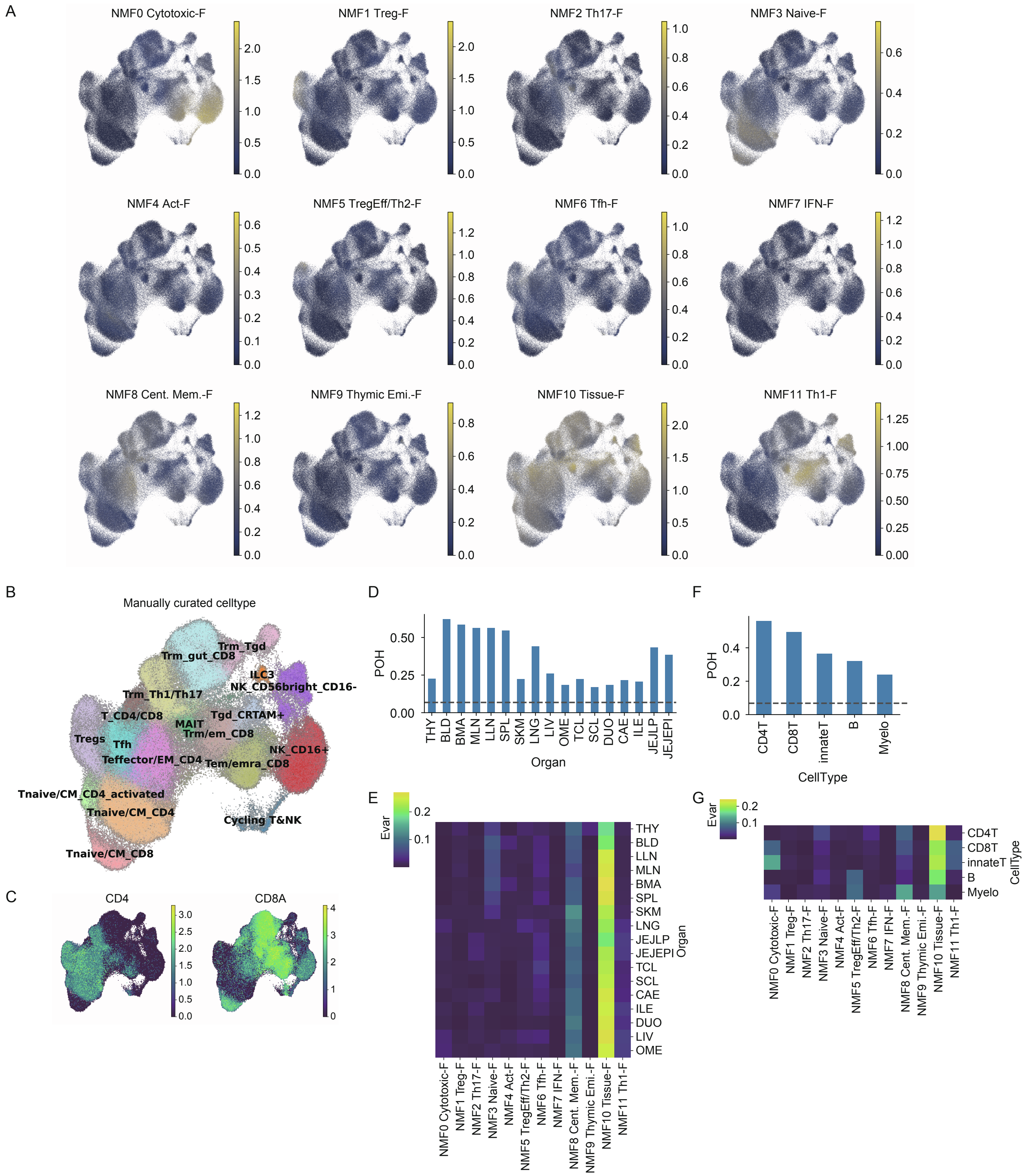
A
NMF0 Cytotoxic-F
NMF1 Treg-F
NMF2 Th17-F
NMF3 Naive-F
NMF4 Act-F
NMF5 TregEff/Th2-F
NMF6 Tfh-F
NMF7 IFN-F
NMF8 Cent. Mem.-F
NMF9 Thymic Emi.-F
NMF10 Tissue-F
NMF11 Th1-F
B
Manually curated celltype
Trm_Tgd
Trm_gut_CD8
ILC3
NK_CD56bright_CD16-
Trm_Th1/Th17
T_CD4/CD8
MAIT
Tgd_CRTAM+
Trm/em_CD8
Tregs
Tfh
NK_CD16+
Teffector/EM_CD4
Tem/emra_CD8
Tnaive/CM_CD4_activated
Tnaive/CM_CD4
Cycling T&NK
Tnaive/CM_CD8
C
CD4
CD8A
D
POH
Organ
THY BLD BMA MLN LLN SPL SKM LNG LIV OME TCL SCL DUO CAE ILE JEJLP JEJEPI
E
Evar
THY BLD LLN MLN BMA SPL SKM LNG JEJLP JEJEPI TCL SCL CAE ILE DUO LIV OME
F
POH
CellType
CD4T CD8T innateT B Myelo
G
Evar
CD4T CD8T innateT B Myelo

Figure S6. NMFproj contributes to interpreting cross-tissue T cells, related to Figure 2

(A) Projected NMF cell feature value of cross-tissue T cells scRNA-seq datasets on the UMAP plots. The T & innate lymphoid cells dataset was used for the analysis (https://www.tissueimmunecellatlas.org/). (B and C) Original cell types (B) and the expression of CD4 and CD8A (C) were shown on the UMAP plots. (D and E) Distribution of POH (D) and Evar (E) in each tissue. THY: Thymus, BLD: Blood, BMA: Bone marrow, MLN: Mesenchymal lymph nodes, LLN: Lung-draining lymph nodes, SPL: Spleen, SKM: Skeletal muscle, LNG: Lung, LIV: Liver, OME: Omentum, TCL: Transverse colon, SCL: Sigmoid colon, DUO: Duodenum, CAE: Caecum, ILE: Ileum, JEJLP: Jejunum lamina propria, JEJEPI: Jejunum epithelial. The dashed line indicates the 97.5 percentile of simulated null distribution (Fig. S3C). (F and G) Distribution of POH (F) and Evar (G) in each cell type. B and Myeloid cells were also added to the analysis. The dashed line indicates the 97.5 percentile of simulated null distribution (Fig. S3C).

Figure S7

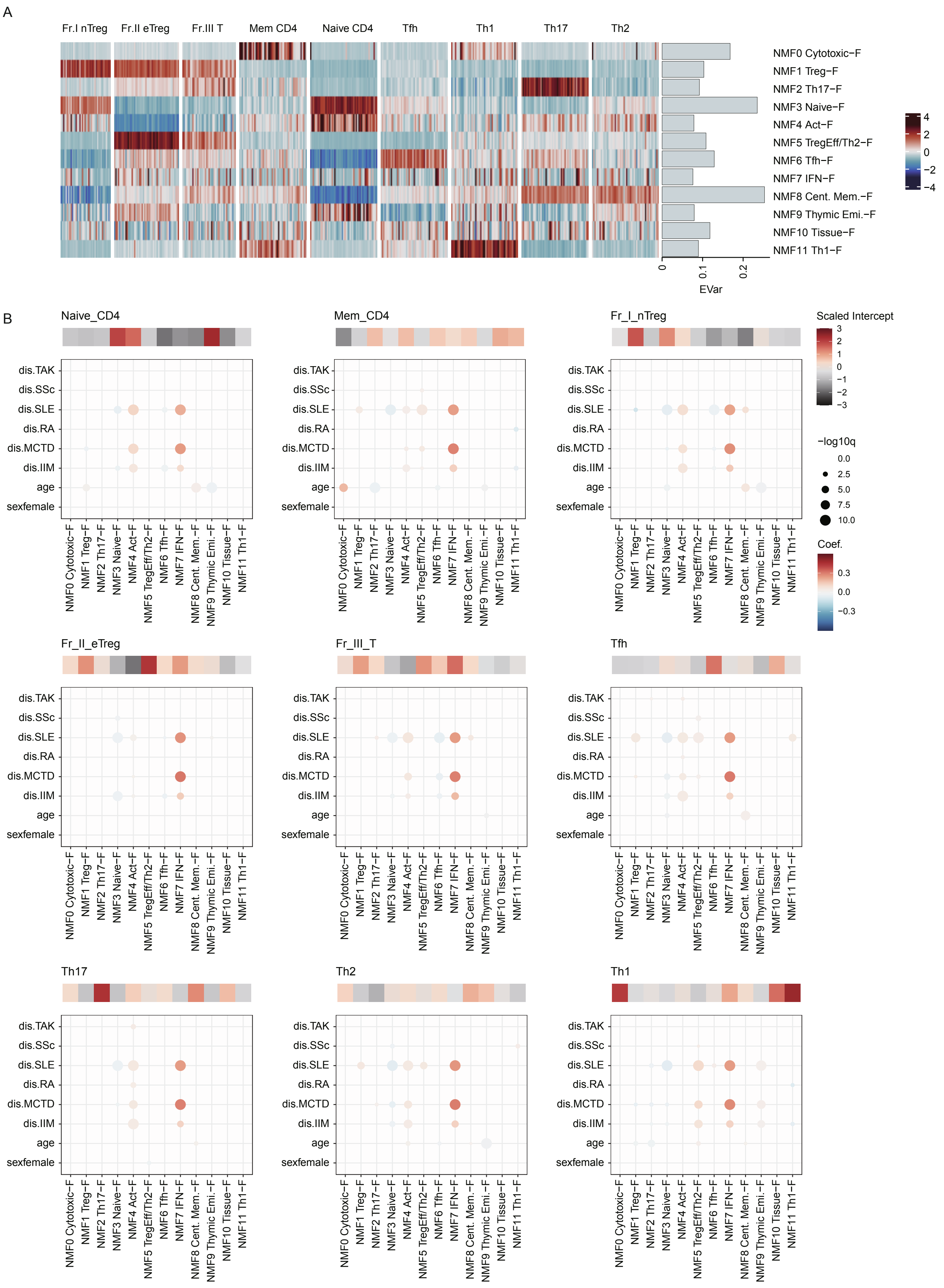
A
Fr.I nTreg
Fr.II eTreg
Fr.III T
Mem CD4
Naive CD4
Tfh
Th1
Th17
Th2
NMF0 Cytotoxic−F
NMF1 Treg−F
NMF2 Th17−F
NMF3 Naive−F
NMF4 Act−F
NMF5 TregEff/Th2−F
NMF6 Tfh−F
NMF7 IFN−F
NMF8 Cent. Mem.−F
NMF9 Thymic Emi.−F
NMF10 Tissue−F
NMF11 Th1−F
EVar
B
Naive_CD4
Mem_CD4
Fr_I_nTreg
Fr_II_eTreg
Fr_III_T
Tfh
Th17
Th2
Th1
dis.TAK
dis.SSc
dis.SLE
dis.RA
dis.MCTD
dis.IIM
age
sexfemale
Scaled Intercept
−log10q
Coef.

Figure S7. NMFproj reveals disease-specific qualitative changes, related to Figure 2

(A) Heatmap showing NMF values of sorted $CD4^+$ T cell fractions collected from autoimmune patients (E-GEAD-397). Explained variance (Evar) was also shown on the right side. (B) Dot plot depicting NMF cell feature changes in each cell type in E-GEAD-397. Dot colors show coefficients, and sizes show the significance of GLM. GLM was performed with a model, cell frequency, or NMF cell feature ~ disease + age + gender. The heatmaps at the top of each plot display the standardized values of the GLM intercept for each feature, representing the baseline activity of each feature in each cell. IIM: idiopathic inflammatory myopathy, MCTD: mixed connective tissue disease, RA: rheumatoid arthritis, SLE: systemic lupus erythematosus, SSc: systemic sclerosis, TAK: Takayasu arteritis.

Figure S8

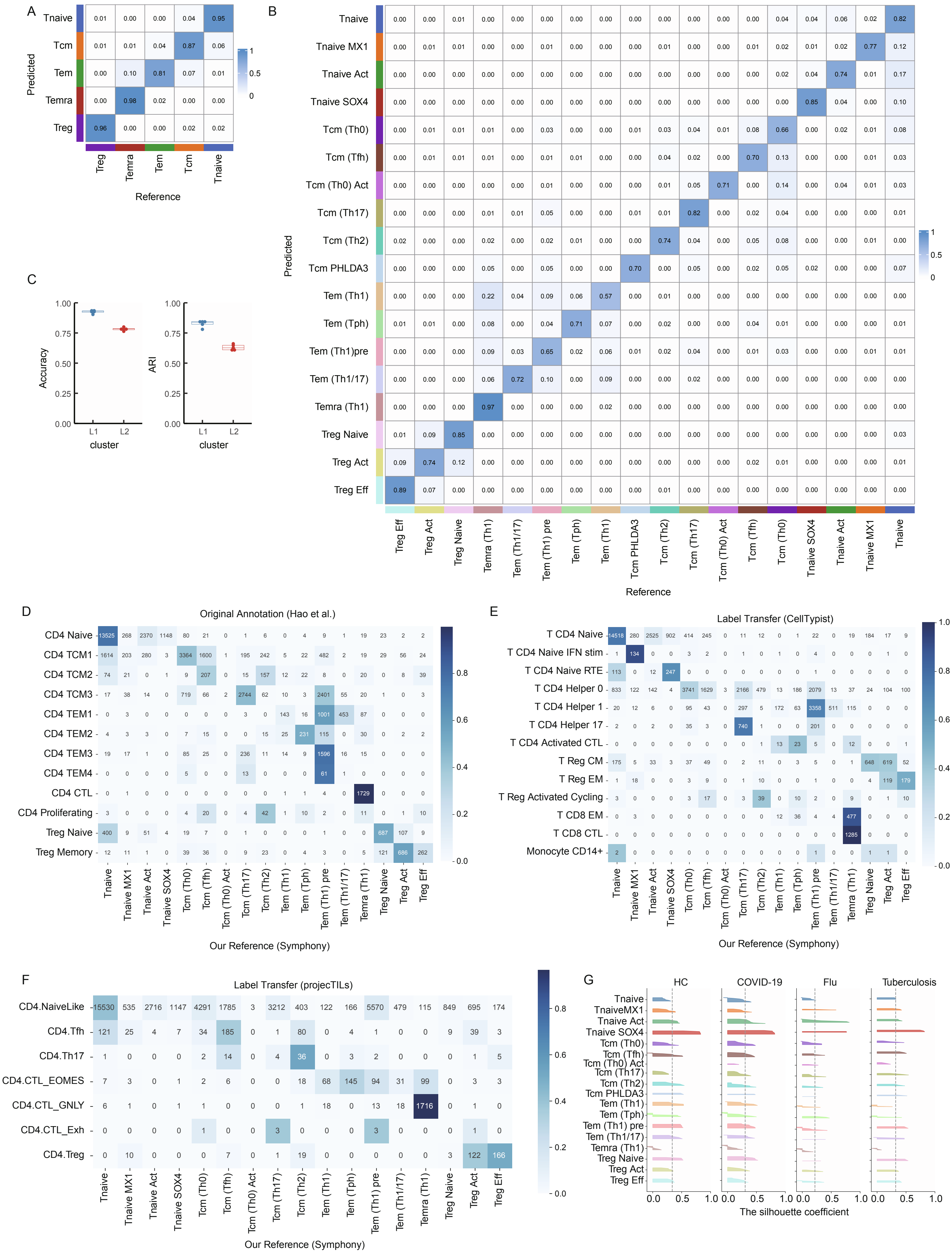

A
Predicted
Reference
B
Predicted
Reference
C
Accuracy
ARI
cluster
D
Original Annotation (Hao et al.)
Our Reference (Symphony)
E
Label Transfer (CellTypist)
Our Reference (Symphony)
F
Label Transfer (projecTILs)
Our Reference (Symphony)
G
HC
COVID-19
Flu
Tuberculosis
The silhouette coefficient

Figure S8. Quality check of reference mapping using Symphony, related to Figure 3

(A-C) Heatmap depicting the precision of reference mapping at L1 (A) and L2 (B) levels, along with plots showing accuracy and Adjusted Rand Index (ARI) (C). We divided our dataset (n=13) into five non-overlapping groups and performed five-fold cross-validation (CV). In this CV, we calculated corrected PCs with Harmony using four groups, constructed a Symphony reference, and mapped the remaining independent sample. These procedures were repeated five times for each fold. We compared the predicted labels with the labels we had pre-annotated to evaluate accuracy. As evaluation metrics, we present the accuracy and ARI at cluster L1 and L2 levels per CV. The lower and upper hinges represent the first quartile and the third quartile. Whiskers show the smallest or largest values that are within 1.5 times the IQR from the hinges. (D) Transferred labels using our pipeline were compared to the original annotations for human PBMC cellular indexing of transcriptomes and epitopes by sequencing (CITE-seq) data from Hao *et al.* The numbers represent the absolute cell count for each paired annotation, while colors denote the fraction relative to the total count in the original study's annotation. (E and F) Transferred labels using our pipeline were compared to labels assigned by CellTypist with the model, COVID19_HumanChallenge_Blood (E) and ProjecTILs with human $CD4^+$ TILs (F) for human PBMC CITE-seq data from Hao et al. The numbers represent the absolute cell count for each paired annotation, while colors denote the fraction relative to the total count in the original study's annotation. (G) Silhouette scores for each sample based on the corrected PCA by Symphony and the cluster L2 assigned by Symphony. Each panel represents a different condition. The number of cells per disease is as follows: Healthy Controls (HC) 7,671 cells, COVID-19 3,475 cells, Flu 421 cells, and Tuberculosis 8,767 cells.

Figure S9

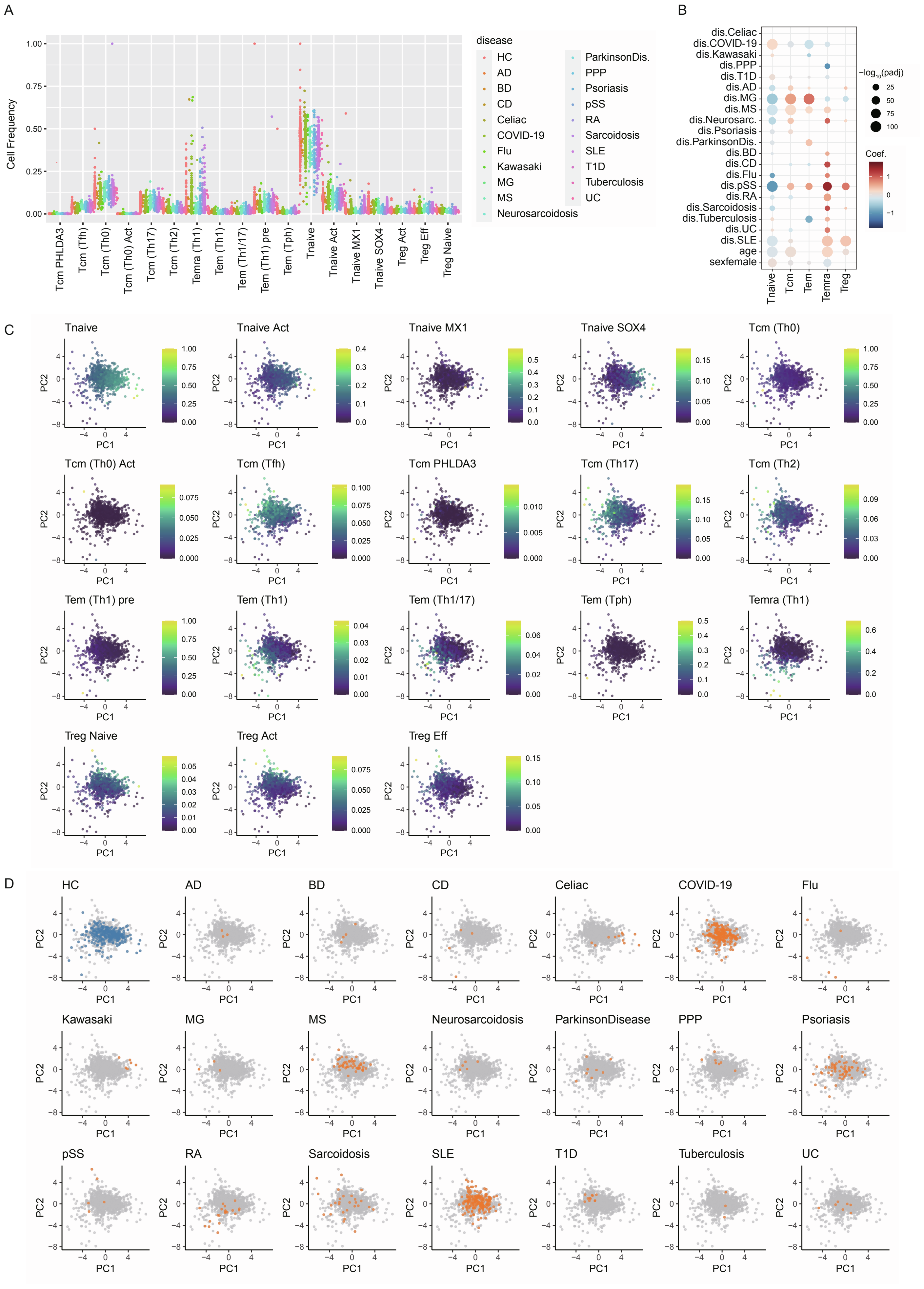
A
B
C
D
Cell Frequency
disease
HC
AD
BD
CD
Celiac
COVID-19
Flu
Kawasaki
MG
MS
Neurosarcoidosis
ParkinsonDis.
PPP
Psoriasis
pSS
RA
Sarcoidosis
SLE
T1D
Tuberculosis
UC
Tcm PHLDA3
Tcm (Tfh)
Tcm (Th0)
Tcm (Th0) Act
Tcm (Th17)
Tcm (Th2)
Temra (Th1)
Tem (Th1)
Tem (Th1/17)
Tem (Th1) pre
Tem (Tph)
Tnaive
Tnaive Act
Tnaive MX1
Tnaive SOX4
Treg Act
Treg Eff
Treg Naive
dis.Celiac
dis.COVID-19
dis.Kawasaki
dis.PPP
dis.T1D
dis.AD
dis.MG
dis.MS
dis.Neurosarc.
dis.Psoriasis
dis.ParkinsonDis.
dis.BD
dis.CD
dis.Flu
dis.pSS
dis.RA
dis.Sarcoidosis
dis.Tuberculosis
dis.UC
dis.SLE
age
sexfemale
Tnaive
Tcm
Tem
Temra
Treg
−log10(padj)
Coef.
PC1
PC2
Treg Naive
Treg Act
Treg Eff
ParkinsonDisease

Figure S9. Quantitative alterations revealed by meta-analysis , related to Figure 3

(A) Swarm plot showing frequencies of cell types in each sample. (B) Dot plot showing changes in cell frequency at cluster L1 resolution. Dot colors show coefficients, and sizes show the significance of the Generalized Linear Model (Methods). Detailed statistics can be found in Table S7. Only significant dots ($p_{adj} < 0.05$) are shown. (C) Cell frequencies of each population are shown on the PCA plots. PCA was performed using cell frequencies of each cluster in each individual. (D) Distribution of samples for each disease on the PCA plot.

Figure S10

A

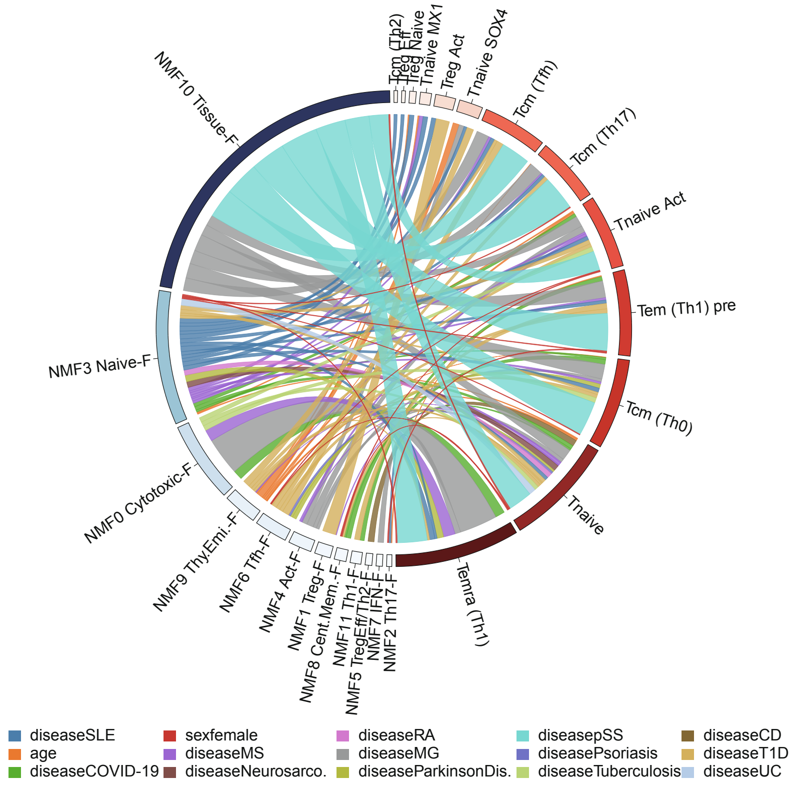

B

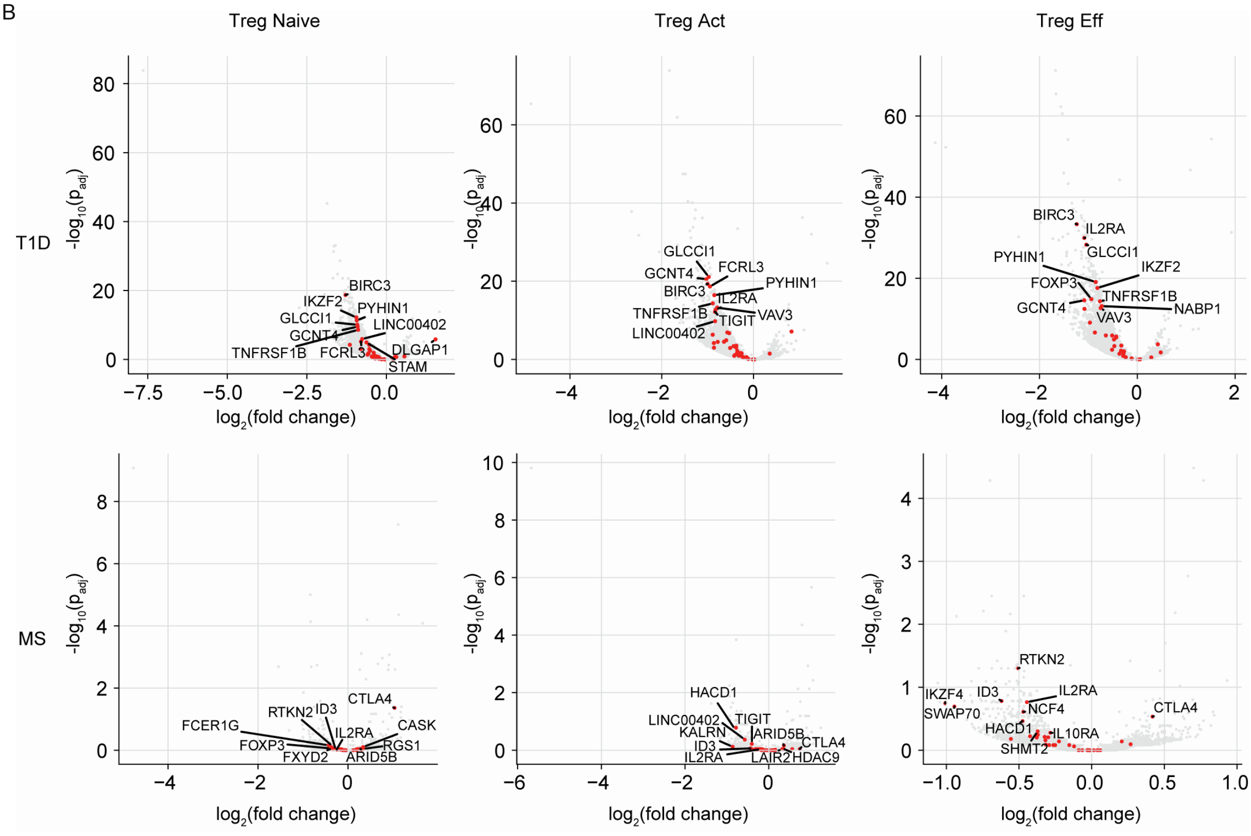

Figure S10. Alterations in $CD4^+$ T cells revealed by meta-analysis, related to Figure 3

(A) Chord diagram showing the top 100 significant associations with negative coefficients between NMF features and cells in each condition, calculated by GLM (Methods). Detailed statistics are shown in Table S9. The thickness of edges indicates the absolute value of the coefficient of GLM, and colors indicate conditions such as diseases, gender, and age.
(B) Volcano plots showing differentially expressed genes in Treg clusters in T1D and MS. Variation in gene expression between diseases and healthy controls in three Treg clusters was calculated using sc.tl.rank_genes_groups (method='t-test_overestim_var') for each disease. To avoid the influence of batch effects between projects, only the GSE144744 dataset was used for MS. Genes with a mean normalized expression value of 0.1 or higher are shown in the volcano plot. Genes that are in the top 50 for NMF1 feature values are marked in red.

Figure S11

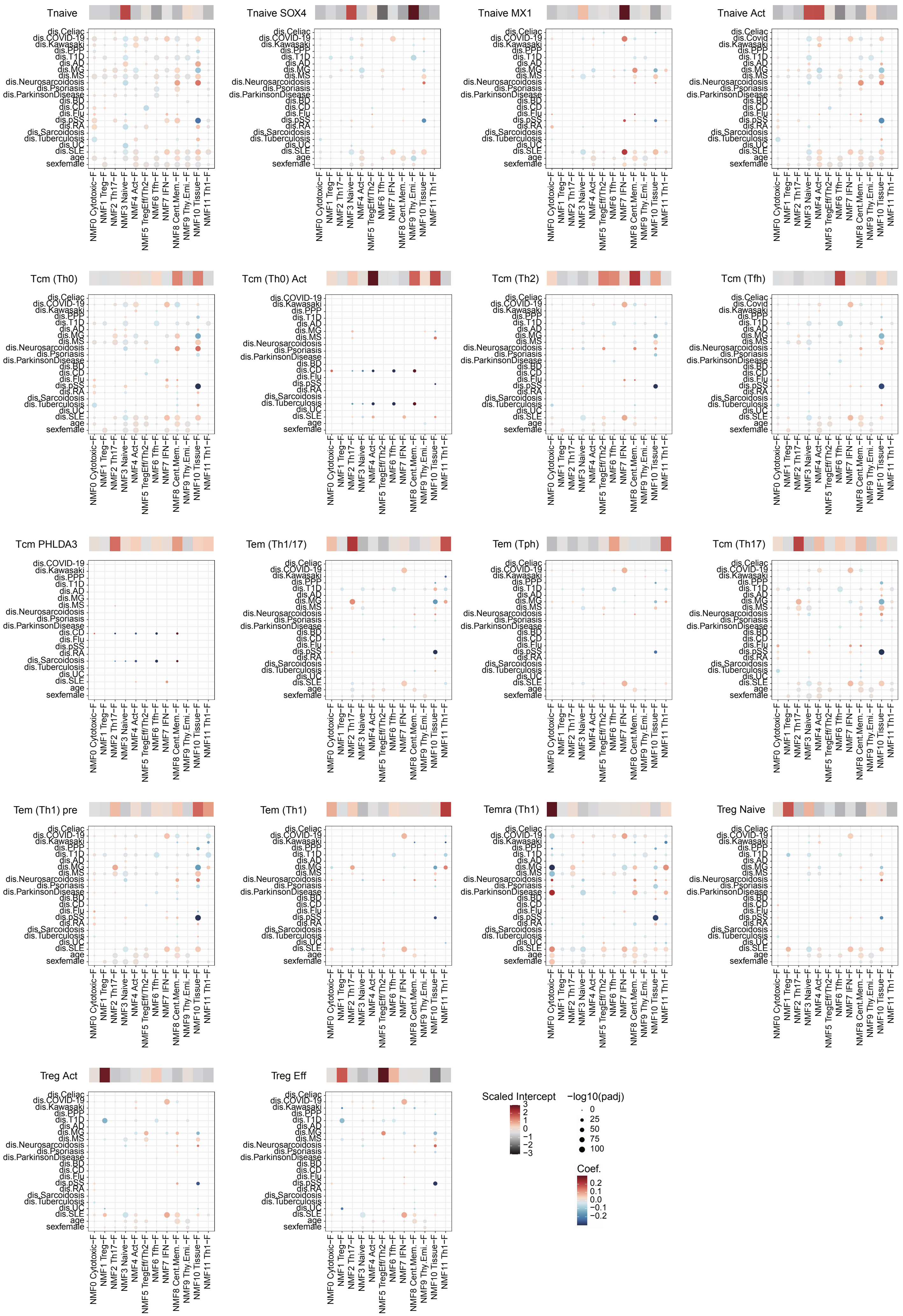
Tnaive
Tnaive SOX4
Tnaive MX1
Tnaive Act
Tcm (Th0)
Tcm (Th0) Act
Tcm (Th2)
Tcm (Tfh)
Tcm PHLDA3
Tem (Th1/17)
Tem (Tph)
Tcm (Th17)
Tem (Th1) pre
Tem (Th1)
Temra (Th1)
Treg Naive
Treg Act
Treg Eff
dis.Celiac
dis.COVID-19
dis.Kawasaki
dis.PPP
dis.T1D
dis.AD
dis.MG
dis.MS
dis.Neurosarcoidosis
dis.Psoriasis
dis.ParkinsonDisease
dis.BD
dis.CD
dis.Flu
dis.pSS
dis.RA
dis.Sarcoidosis
dis.Tuberculosis
dis.UC
dis.SLE
age
sexfemale
NMF0 Cytotoxic−F
NMF1 Treg−F
NMF2 Th17−F
NMF3 Naive−F
NMF4 Act−F
NMF5 TregEff/Th2−F
NMF6 Tfh−F
NMF7 IFN−F
NMF8 Cent.Mem.−F
NMF9 Thy.Emi.−F
NMF10 Tissue−F
NMF11 Th1−F
Scaled Intercept
3
2
1
0
−1
−2
−3
−log10(padj)
0
25
50
75
100
Coef.
0.2
0.1
0.0
−0.1
−0.2

Figure S11. NMF cell feature changes depending on diseases, related to Figure 3

Dot plots depicting NMF cell feature changes in each cell type. Dot colors show coefficients, and sizes show the significance of GLM. GLM was performed with a model, NMF cell feature ~ disease + age + gender + project. Only significant dots ($p_{adj} < 0.05$) are shown. The heatmaps at the top of each plot display the standardized values of the GLM intercept for each feature, representing the baseline activity of each feature in each cell.

Figure S12

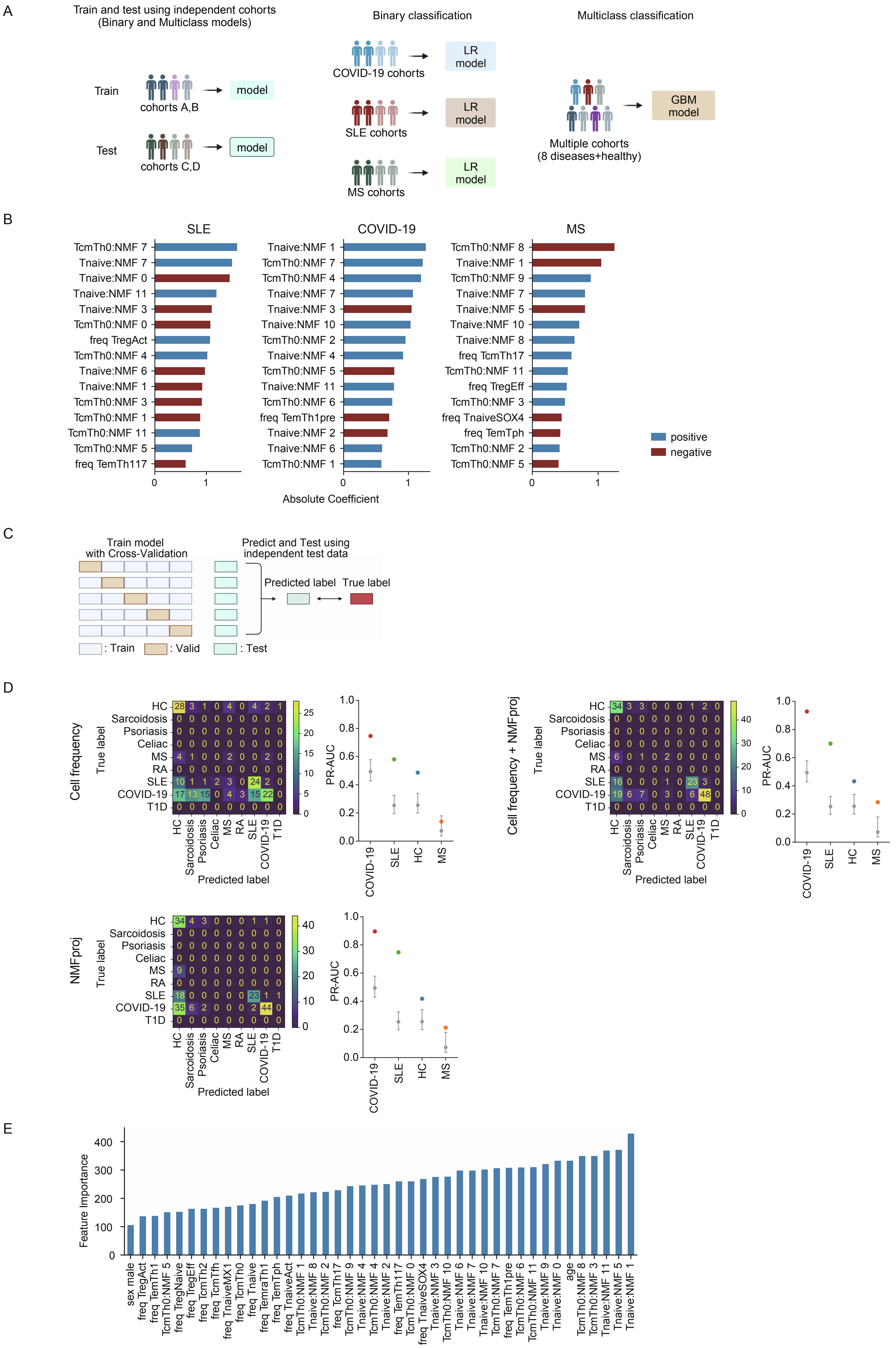
A
Train and test using independent cohorts
(Binary and Multiclass models)
Train
cohorts A,B
model
Test
cohorts C,D
model
Binary classification
COVID-19 cohorts
LR model
SLE cohorts
LR model
MS cohorts
LR model
Multiclass classification
Multiple cohorts
(8 diseases+healthy)
GBM model
B
SLE
TcmTh0:NMF 7
Tnaive:NMF 7
Tnaive:NMF 0
Tnaive:NMF 11
Tnaive:NMF 3
TcmTh0:NMF 0
freq TregAct
TcmTh0:NMF 4
Tnaive:NMF 6
Tnaive:NMF 1
TcmTh0:NMF 3
TcmTh0:NMF 1
TcmTh0:NMF 11
TcmTh0:NMF 5
freq TemTh117
COVID-19
Tnaive:NMF 1
TcmTh0:NMF 7
TcmTh0:NMF 4
Tnaive:NMF 7
Tnaive:NMF 3
Tnaive:NMF 10
TcmTh0:NMF 2
Tnaive:NMF 4
TcmTh0:NMF 5
Tnaive:NMF 11
TcmTh0:NMF 6
freq TemTh1pre
Tnaive:NMF 2
Tnaive:NMF 6
TcmTh0:NMF 1
MS
TcmTh0:NMF 8
Tnaive:NMF 1
TcmTh0:NMF 9
Tnaive:NMF 7
Tnaive:NMF 5
Tnaive:NMF 10
Tnaive:NMF 8
freq TcmTh17
TcmTh0:NMF 11
freq TregEff
TcmTh0:NMF 3
freq TnaiveSOX4
freq TemTph
TcmTh0:NMF 2
TcmTh0:NMF 5
Absolute Coefficient
positive
negative
C
Train model
with Cross-Validation
Predict and Test using
independent test data
Predicted label
True label
: Train
: Valid
: Test
D
Cell frequency
Cell frequency + NMFproj
NMFproj
True label
Predicted label
HC
Sarcoidosis
Psoriasis
Celiac
MS
RA
SLE
COVID-19
T1D
PR-AUC
COVID-19
SLE
HC
MS
E
Feature Importance
sex male
freq TregAct
freq TemTh1
TcmTh0:NMF 5
freq TregNaive
freq TregEff
freq TcmTh2
freq TcmTfh
freq TnaiveMX1
freq TcmTh0
freq Tnaive
freq TemraTh1
freq TemTph
freq TnaiveAct
TcmTh0:NMF 1
Tnaive:NMF 8
TcmTh0:NMF 2
freq TcmTh17
TcmTh0:NMF 9
Tnaive:NMF 4
TcmTh0:NMF 4
Tnaive:NMF 2
freq TemTh117
TcmTh0:NMF 0
freq TnaiveSOX4
Tnaive:NMF 3
TcmTh0:NMF 10
Tnaive:NMF 6
Tnaive:NMF 7
Tnaive:NMF 10
TcmTh0:NMF 7
freq TemTh1pre
TcmTh0:NMF 6
TcmTh0:NMF 11
Tnaive:NMF 9
Tnaive:NMF 0
age
TcmTh0:NMF 8
TcmTh0:NMF 3
Tnaive:NMF 11
Tnaive:NMF 5
Tnaive:NMF 1

Figure S12. Predicting Autoimmune Diseases Based on $CD4^+$ T Cell Profiles Using Machine Learning, related to Figure 3

(A) Overview of autoimmune disease prediction using machine learning. LR: Logistic Regression, GBM: Gradient Boosting Machine. (B) Bar plots showing absolute coefficient for logistic regression models. The top 15 variables are shown. Colors indicate whether the coefficient is positive or negative. (C) Strategy for multiclass classification by machine learning. The training was performed with cross-validation. The evaluation was performed using the independent dataset of training datasets. (D) Evaluations of models trained by cell frequencies (upper left panel), by NMFproj values in Tnaive and Tcm (Th0) (lower left panel), and by both cell frequencies and NMFproj values (right panel). The confusion matrix (left) and PR-AUC (right) are shown. The dashed lines in the PR-AUC plot show the expected PR-AUC scores in random models. The number of samples used for the training is 263, 27, 62, 11, 43, 20, 156, 116, 11 subjects for HC, sarcoidosis, psoriasis, celiac disease, MS, RA, SLE, COVID-19, and T1D, and evaluated on 89, 43, 43, and 9 subjects from independent data sets. Error bars indicate a 95% confidence interval for the random model calculated by 500 times permutations of true labels. The accuracy for each model is cell frequency model: 0.413, NMF model: 0.484, and cell frequency + NMF model: 0.527, respectively. (E) Bar plot showing feature importance of variables in the cell frequency + NMF GBM model.

Figure S13

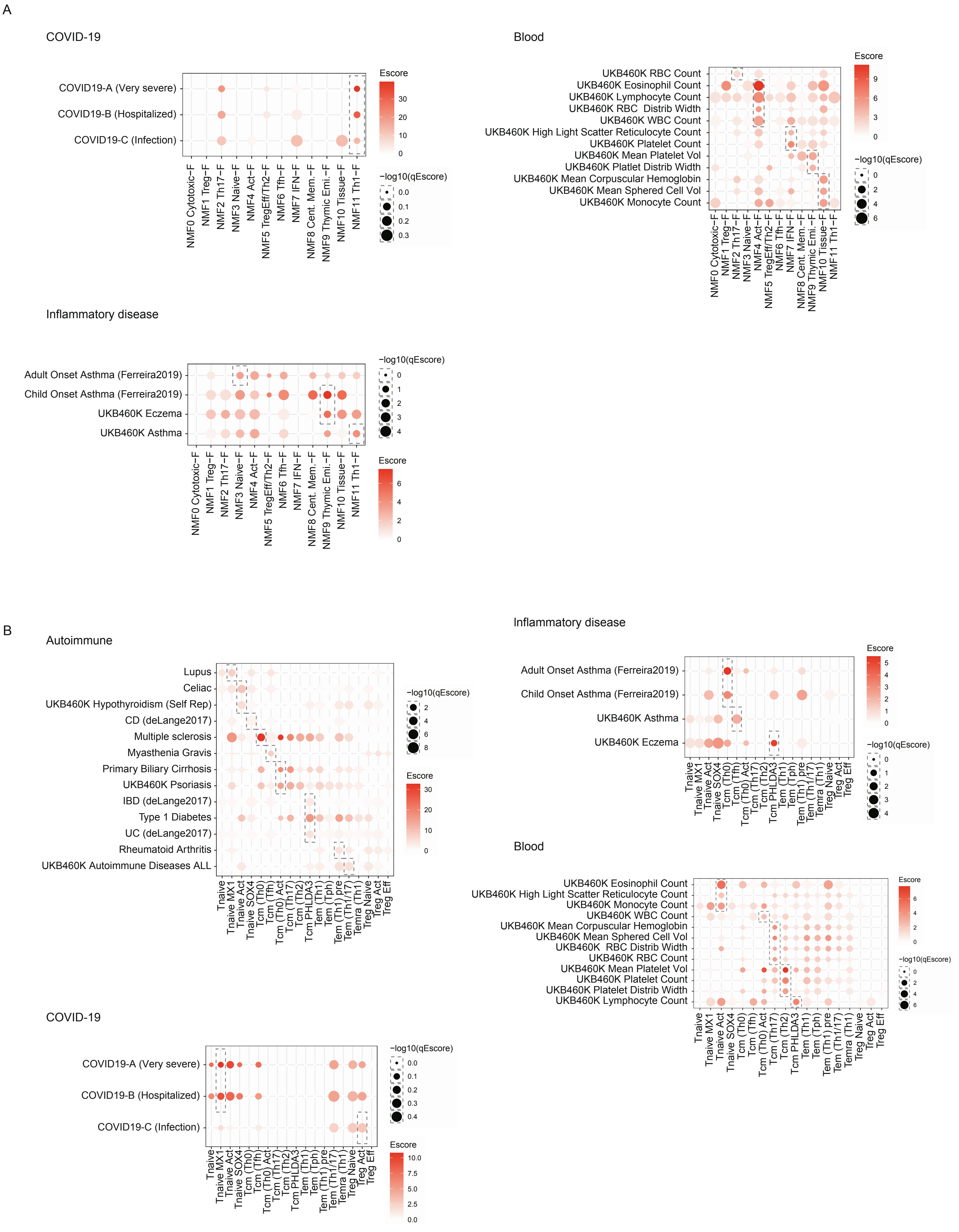

A
COVID-19
COVID19-A (Very severe)
COVID19-B (Hospitalized)
COVID19-C (Infection)
NMF0 Cytotoxic−F
NMF1 Treg−F
NMF2 Th17−F
NMF3 Naive−F
NMF4 Act−F
NMF5 TregEff/Th2−F
NMF6 Tfh−F
NMF7 IFN−F
NMF8 Cent. Mem.−F
NMF9 Thymic Emi.−F
NMF10 Tissue−F
NMF11 Th1−F
Escore
−log10(qEscore)
Blood
UKB460K RBC Count
UKB460K Eosinophil Count
UKB460K Lymphocyte Count
UKB460K RBC Distrib Width
UKB460K WBC Count
UKB460K High Light Scatter Reticulocyte Count
UKB460K Platelet Count
UKB460K Mean Platelet Vol
UKB460K Platlet Distrib Width
UKB460K Mean Corpuscular Hemoglobin
UKB460K Mean Sphered Cell Vol
UKB460K Monocyte Count
Inflammatory disease
Adult Onset Asthma (Ferreira2019)
Child Onset Asthma (Ferreira2019)
UKB460K Eczema
UKB460K Asthma
B
Autoimmune
Lupus
Celiac
UKB460K Hypothyroidism (Self Rep)
CD (deLange2017)
Multiple sclerosis
Myasthenia Gravis
Primary Biliary Cirrhosis
UKB460K Psoriasis
IBD (deLange2017)
Type 1 Diabetes
UC (deLange2017)
Rheumatoid Arthritis
UKB460K Autoimmune Diseases ALL
Tnaive
Tnaive MX1
Tnaive Act
Tnaive SOX4
Tcm (Th0)
Tcm (Tfh)
Tcm (Th0) Act
Tcm (Th17)
Tcm (Th2)
Tcm PHLDA3
Tem (Th1)
Tem (Tph)
Tem (Th1) pre
Tem (Th1/17)
Temra (Th1)
Treg Naive
Treg Act
Treg Eff
Inflammatory disease
Adult Onset Asthma (Ferreira2019)
Child Onset Asthma (Ferreira2019)
UKB460K Asthma
UKB460K Eczema
Blood
UKB460K Eosinophil Count
UKB460K High Light Scatter Reticulocyte Count
UKB460K Monocyte Count
UKB460K WBC Count
UKB460K Mean Corpuscular Hemoglobin
UKB460K Mean Sphered Cell Vol
UKB460K RBC Distrib Width
UKB460K RBC Count
UKB460K Mean Platelet Vol
UKB460K Platelet Count
UKB460K Platelet Distrib Width
UKB460K Lymphocyte Count
COVID-19
COVID19-A (Very severe)
COVID19-B (Hospitalized)
COVID19-C (Infection)

Figure S13. Partitioned heritability, related to Figure 4

(A and B) Dot plots showing partitioned heritability of diseases across NMF gene features (A) or cell types (B). Duplicated traits were removed for the visualization. Full statistics are shown in Tables S11 and 12.

ID	Gender	Age	Disease
**STR4**	Female	31	HC
**STR5**	Male	50	HC
**STR21**	Male	27	HC
**MS01**	Male	36	MS
**MS02**	Female	23	MS
**MS03**	Female	39	MS
**MS04**	Male	24	MS
**MG01**	Male	72	MG
**MG02**	Male	51	MG
**MG03**	Male	46	MG
**SL02**	Female	58	SLE
**SL03**	Female	62	SLE
**SL04**	Male	44	SLE

Table S1. Patient information, related to Figure 1

The information of patients used in scRNA-seq experiments.
